# Setting method of exit advance guide signs in mountainous expressway tunnel based on information quantization theory

**DOI:** 10.1371/journal.pone.0281842

**Published:** 2023-02-16

**Authors:** Ting Shang, Yifei Wu, Peng Wu, Hucheng He, Bao You

**Affiliations:** 1 School of Traffic & Transportation, Chongqing Jiaotong University, Chongqing, P. R. China; 2 Department of Engineering Management, Chong Qing Feng Jian Expressway Co., Ltd., Chongqing, P. R. China; Qatar University, QATAR

## Abstract

Driving behavior in expressway tunnels is more complicated than in common roadbed sections because of differences in illuminance, visual range, speed perception, and reaction time. To explore the setting method of exit advance guide signs in expressway tunnels and improve the effectiveness of drivers’ recognition of them, we propose 12 layout forms based on information quantification theory. In experiments, UC-win/Road was used to build a simulation scene, and the recognition reaction time of 12 element combinations of exit advance guide signs of different subjects was collected through an E-Prime simulation experiment. The loading effectiveness of the signs was analyzed based on the subjective workload and comprehensive evaluation scores of different subjects. The results are the following. The width of the exit advance guide sign layout in the tunnel is negatively correlated with the height of Chinese characters and the distance between the characters and the edge of the sign. The larger the height of Chinese characters and the distance between them and the edge of the sign, the smaller the maximum layout width of the sign. Considering the driver’s reaction time, subjective workload, sign recognition, amount of sign information, sign accuracy, and sign safety of 12 different information combinations, we suggest that exit advance guide signs in tunnels should be laid out as Chinese/English place name + distance + guide arrow.

## 1. Introduction

The tunnel plays an important role in overcoming natural traffic obstructions and decreasing driving distances. However, due to the influence of terrain, geology, land use restrictions, and an increase in road network density due to the construction of new roads, the distance between the tunnel and the exit of the main line interchange in front is often too small. For example, in the section from the Huangcaoling tunnel to the Wulong West interchange in the direction of the G65 Baomao Expressway in Wulong district, Chongqing, the distance from the tunnel exit to the start of the transition section of the deceleration lane of the West Wulong Interchange is 310 m, so the 500-m, 1-km, and 2-km exit advance guide signs must be set in the tunnel. After driving out of a tunnel with limited space, low visibility, and a monotonous environment, in addition to being affected by the “white hole” effect, drivers face traffic interweaving problems such as lane changes and merges in a short distance, and this additional information increases the load on drivers [1–3] and elevates the driving risk.

In a highway tunnel exit to an interchange with a small spacing section, the interchange exit advance guide sign provides important information to guide drivers to leave the main line safely and smoothly. The two design specifications, namely *Road traffic signs and markings Part 2*: *road traffic signs* (GB5768 2–2009) and *Road traffic signs and markings Part 2*: *road traffic signs* (GB5768), which will be implemented in October 2022, stipulate that exit advance guide signs and exit action point signs shall be set 2 km, 1 km, and 500 m away from the reference point of expressway interchanges, and exit signs have attached exit number signs [4]. However, due to the limitations of tunnel construction, exit advance guide signs set in tunnels cannot be set according to the requirements. The layout size and information design of exit signs are limited, and the specifications lack clear guidance for the setting of exit advance guide signs in tunnels, which creates challenges for effectively setting them. Therefore, exploring the correct setting method of interchange exit advance guide signs in tunnels is conducive to the rapid and accurate acquisition of key information for drivers, improving recognition of such signs, and preparing for an exit.

To analyze the setting method of exit advance guide signs in mountainous expressway tunnels—after considering the influence of tunnel clearance on the layout size and information setting of exit advance guide signs—12 types of exit advance guide signs with different information combinations are proposed based on information quantification theory, and signs with different combinations of information are evaluated by an E-Prime simulation experiment and subjective load technology, laying a theoretical foundation for their setting.

## 2. Literature review

### 2.1 Relationship between visual load and reaction time in tunnels

The contribution rate of the driver’s vision to driving behavior is about 80% [3], and the visual load is closely related to the level of driving safety. Most studies have used eye movement indexes such as pupil area, fixation time, blinks, and scanning speed to reflect a driver’s load change during driving. Du [5,6] pioneered a driving visual load evaluation method based on the pupil area’s change rate and the maximum transient velocity value of the pupil area (MTPA). Researchers [7–9] have used this index to study the influence of visual load changes on driving safety in long highway tunnels, subsea tunnels, extra-long tunnels, and high-tunnel-ratio sections.

In long tunnels, tunnel lighting influences not only visual performance but reaction time. Liu [10] analyzed the relationship between the LED spectrum, correlated color temperature (CCT), and reaction time. Liang [11] measured reaction times under different values of luminance, correlated color temperature (CCT), eccentricity, and contrast. The results showed that visual performance can be improved by increasing the CCT of light sources in tunnels. Dong [12] analyzed the impact of LED color rendering on the reaction time of human eyes in tunnel interior zones.

Drivers rarely focus exclusively on driving; they are distracted by passengers, navigation systems, smartphones, and driver assistance systems. To create intelligent human-vehicle interfaces and reduce visual load during secondary tasks, Tian [13] studied display positions for a haptic rotary device-based integrated in-vehicle infotainment interface. Lamble [14] investigated drivers’ cognitive loads and detection thresholds while performing mobile phone-related tasks. Van Winsum [15] studied the independent effects of cognitive and visual loads on visual detection response task (VDRT) reaction times in a driving simulator and unraveled the attentional processes underlying the effects. Fan [16] established a comprehensive evaluation model for mental workload and found that with the increase in task difficulty. Kim [17] found that a combined visual and auditory alarm was most effective in terms of drivers’ cognitive loads.

Most studies have focused on the relationship between visual load and reaction time. However, an ordinary highway and a tunnel have different geometric designs, speed limits, driving environments, and lighting. Therefore, it is important to analyze the method of setting exit advance guide signs in tunnels.

### 2.2 Setting methods of exit advance guide signs in highway

Setting methods of exit advance guide signs has been the subject of much research. Upchurch [18] evaluated four exit advance guide sign schemes on one- and two-lane expressways through a comprehensive weighting method. Qiao [19] studied the layout form and setting method of American interchange regional guide signs by comparative experiment. Fitzpatrick [20] analyzed the reaction time of drivers’ recognition of guide signs in six layout forms of the expressway through simulation experiments and evaluated their setting effect. Song [3] calculated the setting distance of traffic exit advance guide signs according to an algorithm considering the deviation rate of the average speed curve. Yao [21] evaluated the effectiveness of traffic guide signs at intersections. Zwahlen [22] investigated the effectiveness of setting ground-mounted diagrammatic advance guide signs in urban multi-lanes leading to freeways.

As bottleneck sections of road networks, tunnels see frequent traffic accidents [23,24]. In conditions of complex geometry and restricted warning times, alternative freeway guide signs can improve driver safety. This finding verifies Arup’s conclusion that alternative road signs can induce drivers to enter the exit lane earlier, thereby reducing the probability of changing lanes and missing the exit [25]. Huang [26] conducted a driving simulation experiment consisting of five design alternatives of advance guide signs and two exit ramp spacing variations. Wang [27] studied highway tunnel traffic signs for feature extraction and selection based on environmental factors and used a decision tree classification algorithm to simplify this complex problem. Wu [28] proposed the location of signs in the straight, horizontal, and vertical curve sections of a tunnel based on the driver’s visual continuity. Yan [29] proposed a model for setting traffic signs in adjacent tunnel groups for safety based on ergonomics and traffic theory. Song [30] obtained eye movement and motion data for a 380-m tunnel where three layouts of signs and markings were installed. Shang [31,32] proposed setting separate exit advance guide signs in a tunnel and evaluated the sign setting effect based on a Markov model.

Only a few publications on the settings of exit advance guide signs are set in a tunnel environment, and some are urban tunnels and tunnel groups. However, highway tunnels and urban tunnels are quite different in terms of alignment design, traffic volume, traffic composition, operation speed, and illumination, as shown in Table 1.

**Table 1:** Statistical difference between highway tunnel and urban tunnel.

Speed (km/h)	Urban tunnel	Trunk road	60、50、40						
		Secondary road	50、40、30						
		Branch road	40、30、20						
	Highway tunnel		60、80、100						
Traffic composition	Urban tunnel	Passenger cars, Motorcycles, Electric vehicles, pickup truck							
	Highway tunnel	Passenger cars, Freight cars							
Traffic status difference	Urban tunnel	The traffic volume of urban tunnels is obviously unbalanced. The commuting time is generally seriously congested, the average running speed is about 20 km/h, and the traffic capacity is seriously reduced. The traffic volume distribution in other daytime periods is relatively average, while in night time, the traffic volume is less. The average running speed is generally close to the design speed value, and basically shows strong directional characteristics.							
	Highway tunnel	The probability of congestion or slow-moving in the highway tunnel is low, the traffic volume is relatively uniform in each period of the day, and the running speed is close to the design speed value. The traffic volume of highway tunnel at night is smaller at night and less at night.							
Tunnel lighting	Urban tunnel	Urban traffic flow is large and the traffic environment is complex. In order to ensure traffic safety, urban tunnels should be equipped with lighting no matter how long they are.							
	Highway tunnel	When the length of a highway tunnel is more than 200m, lighting should be provided. Lighting is not required for a short tunnel							
Linear design	Urban road	Design speed	100	80	60	50	40	30	20
		Minimum radius of circular curve	1600	1000	600	400	300	150	70
		Minimum length of circular curve	85	70	50	40	35	25	20
	Highway	Design speed	120	100	80	60	40	30	20
		Minimum radius of circular curve	1000	700	400	200	100	65	30
		Minimum length of cyclotron line	100	85	70	50	35	25	20

### 2.3 Application of information quantization theory

Most countries provide specifications and standards for the design of traffic signs, such as the *Manual of Uniform Traffic Control Devices* (MUTCD) of the Federal Highway Administration of the United States, the *European Convention on road signs and signals*, the *Manual of traffic signs and markings* of Canada, and *Road traffic signs and markings Part 2*: *road traffic signs* (GB 5768.2–2022) of China, These standards and norms play an important guiding role in the scientific setting of traffic signs.

The traffic signs should convey an appropriate amount of information to meet needs, to be effective. The amount of information can represent the degree to which they reasonably meet demand, which determines their reception degree [33]. However, there is no unified standard for the quantification and threshold calculation of traffic sign information. Sign information can be quantified by either direct or indirect measurement. Direct measurement methods, such as the information unit method [34] and the road name method, are used to count the amount of intuitive and easy-to-calculate information. Indirect measurement methods are used to calculate the probability of understanding, or they directly ask about subjects’ feelings, such as through the National Highway Cooperative Research Project (NCHRP) subjective evaluation [35] and Shannon formula [36].

Yu [37] adopted the strict definition of information from Shannon’s information theory and provided procedures for quantifying the effective provision of traveler information. Guo [38] established the traffic sign information model under the condition of an expressway network by using information theory and found that the threshold of traffic sign information at the exit of an expressway is 194 bits, and the sign should be divided into 7 sub-modules at most. Shinar [39] concluded that as long as traffic signs comply with the principles of standardization, appropriate content, familiarity, and readability, drivers’ recognition ability of them can be improved. Liu [40] analyzed the relationship between the information quantity of urban roadside traffic signs and drivers’ visibility based on information transmission. Guo [41] proposed a fast recognition algorithm based on speeded-up robust features (SURF) for static traffic sign information of highways. Lyu [42] revealed that the workload is highly related to the amount of information on traffic signs, and reaction time increases with the information grade while driving experience and gender are not significant. Babic [43] analyzed market-ready traffic sign recognition systems in cars.

Visual workload contributes to human errors in road accidents and potentially adverse incidents. Traffic sign information is indispensable to road traffic. Information quantification theory is widely used in the field of transportation. Therefore, it can be applied to the design of tunnel exit advance guide signs.

### 2.4 Research summary

To sum up, researchers have mainly conducted a large number of research on the relationship between visual load and reaction time, the design method of signs and the quantification theory of information. Among them, the relationship between visual load and reaction time and the quantification theory of information consider the impact of sign design on the driver’s recognition from the perspective of ergonomics, providing theoretical support for the design of traffic signs. It also provides reference for designers engaged in road traffic safety. However, the existing research mainly focuses on the design of guide signs for ordinary road sections that are not limited by the boundary space, including urban road intersections and diversion sections of expressways. However, the research on guide signs in tunnels that are limited by the boundary is less, and the theoretical guidance is relatively weak. Setting guide signs in tunnels is of great significance for drivers to make path decisions after driving out of tunnels. For this reason, based on the method of information quantification theory, we have carried out relevant research on the setting of signs in the tunnel scene, mainly focusing on the text content and size of the indicator signs, hoping to standardize the setting of signs in this scene and provide theoretical reference in practical work.

## 3. Methods

### 3.1 Overview of research methods

**Fig 1:**
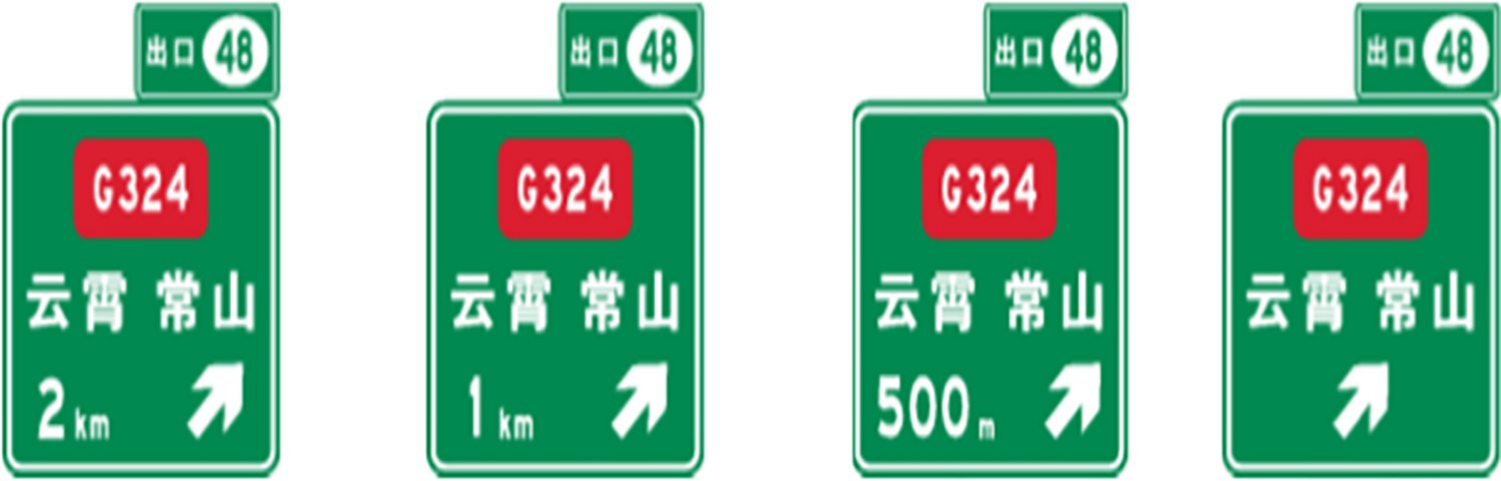
Expressway interchange exit advance guide signs.

**Fig 2:**
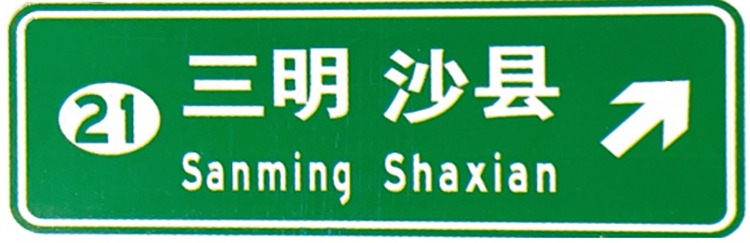
Example of exit advance guide sign in tunnels.

In the current design method, the layout design of expressway exit advance guide sign is shown in Fig 1. As the tunnel is a relatively narrow confined space, the exit advance guide signs set in the tunnel cannot be designed according to Fig 1 due to the influence of the building limits. Therefore, the Code for the Setting of Highway Traffic Signs and Markings (JTG D82-2009) stipulates that the exit advance guide sign set in the tunnel should not affect the ventilation and monitoring facilities, and its layout content can be adjusted appropriately, as shown in Fig 2, The characters and patterns in the exit notice signs can be reduced as a whole according to the requirements of building limits. The current specifications do not make specific provisions for the setting of indication signs in the tunnel.

Therefore, this paper designs the guide signs in the tunnel based on the method of information quantification theory. Firstly, the maximum size of the sign is determined according to the building limits in the tunnel, and then quantify the different types of information in the sign based on the information quantification theory to obtain the total amount of information in different sign pages, and then obtain 12 different types of guide signs according to the combination form of different elements, and then use simulation experiments to model the tunnel scene and sign boards, The E-Prime simulation platform was used to determine the average recognition reaction time of drivers to different signs. Finally, the best combination form of guide signs is comprehensively determined according to the subjective load of drivers, the recognition time of signs, the amount of sign information and the accuracy of recognition signs.

### 3.2 Contribution of research methods

Based on the method of information quantification theory, this paper designs the tunnel exit warning signs, which has two main contributions. Firstly, it provides an effective scheme. Due to the influence of clearance, the exit advance guide signs in the tunnel cannot be set up like those set in ordinary subgrade sections. Therefore, this paper proposes a method suitable for setting exit advance guide signs in tunnel sections, and evaluates its effectiveness, which can provide a theoretical basis for the design of signs in the tunnel exit scene later, and also provide some reference for designers engaged in road traffic safety. The other is to simplify the layout capacity of traffic signs under the condition of satisfying the driver’s visibility, and correspondingly reduce the layout area, so the overall investment will also be reduced accordingly. On the premise of ensuring safety, the resource investment will be reduced, and to a certain extent, which can meet the benefit ratio of investment to a certain extent.

### 3.3 Layout size settings

**Fig 3:**
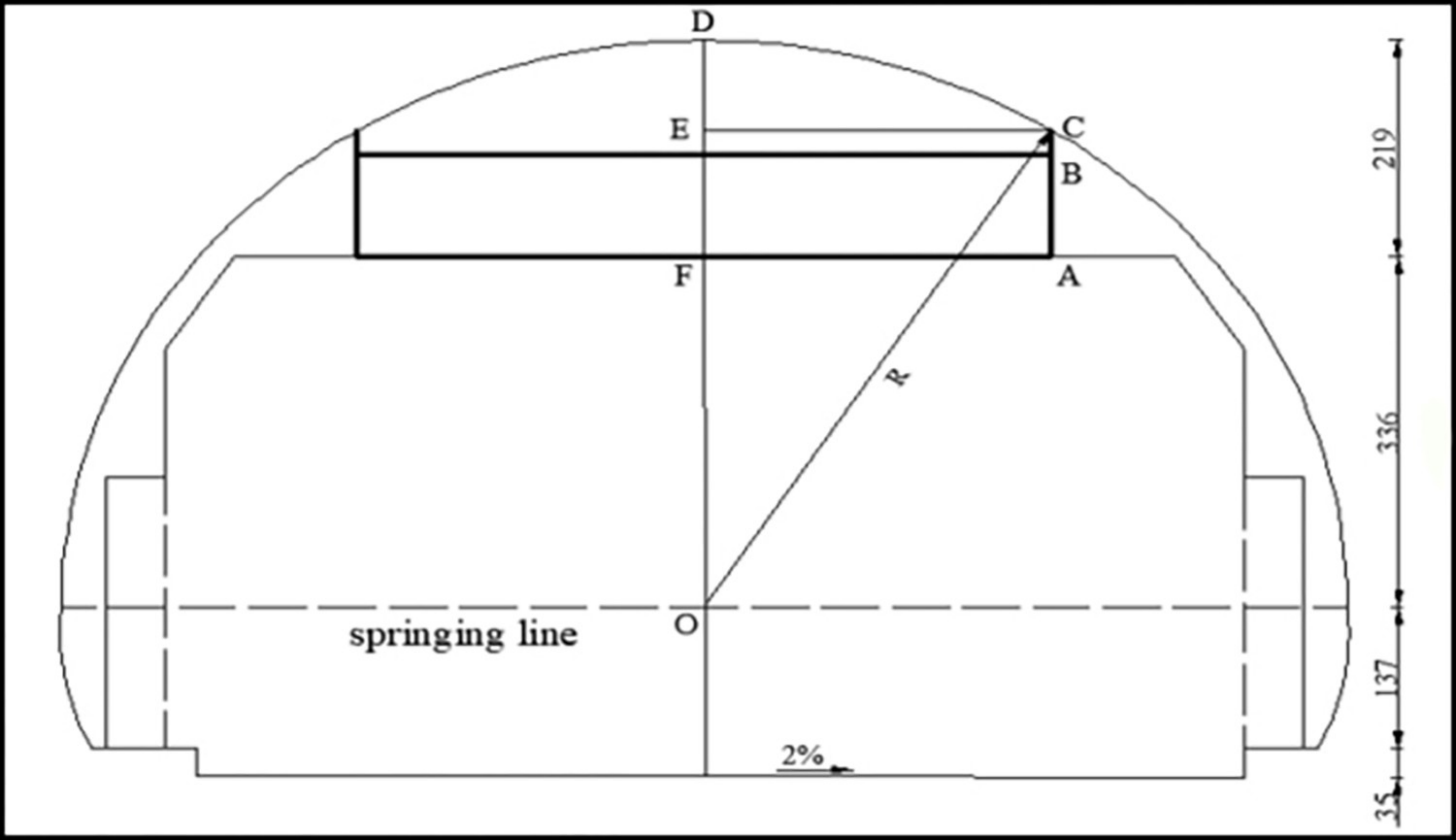
Inner contour drawing of one-way, two-lane highway tunnel.

The tunnel is a relatively closed space. The size and content of exit advance guide signs in tunnels cannot be designed according to the *Specification for Layout of Highway Traffic Signs and Markings* (TG D82-2009). Exit advance guide signs in tunnels cannot influence ventilation and monitoring facilities. The layout content can be adjusted appropriately, and the text and patterns can be reduced according to building requirements [21]. Under the condition that the sign does not intrude into the limits of the building, the schematic diagram of the setting of an exit advance guide sign with the maximum layout size in a tunnel is shown in Fig 3.

Assuming that the installation height of the exit advance guide signs that can be set in the tunnel is BC, the maximum width L of the sign layout is 2AF, and the maximum height H is AB, so according to the geometric calculation, we obtain

$$R^{2}={\mathrm{AF}}^{2}+{\left(\mathrm{BC}+\mathrm{AB}+\mathrm{OF}\right)}^{2}$$ (1)

According to *Road Traffic Signs and Markings* GB5768-2009, on highways where the speed is set at 80 km/h, the height of Chinese characters on road signs is 50~60 cm, pinyin letters are $\frac{1}{3}h$~$\frac{1}{2}h$ (“h” refers to the height of Chinese characters), the text interval is more than $\frac{1}{10}h$, the line spacing is $\frac{1}{5}h$~$\frac{1}{3}h$, and the minimum distance from the edge of the sign is $\frac{2}{5}h$. The installation height of signs in tunnels is generally 5~10 cm. The contour radius *R* of the two-lane highway tunnel is 555 cm; that is, OC = OD = 555 cm, and the distance between the top of the tunnel and the building limit is 219 cm; that is, DF = 219 cm, so OF = OD − DF = 555 − 219 = 336 cm. In this paper, we take BC = 5 cm. Therefore, the maximum layout length of an exit advance guide sign that can be set in a tunnel is

$$L=2\sqrt{555^{2}-{\left(\mathrm{AB}+341\right)}^{2}}$$ (2)

**Fig 4:**
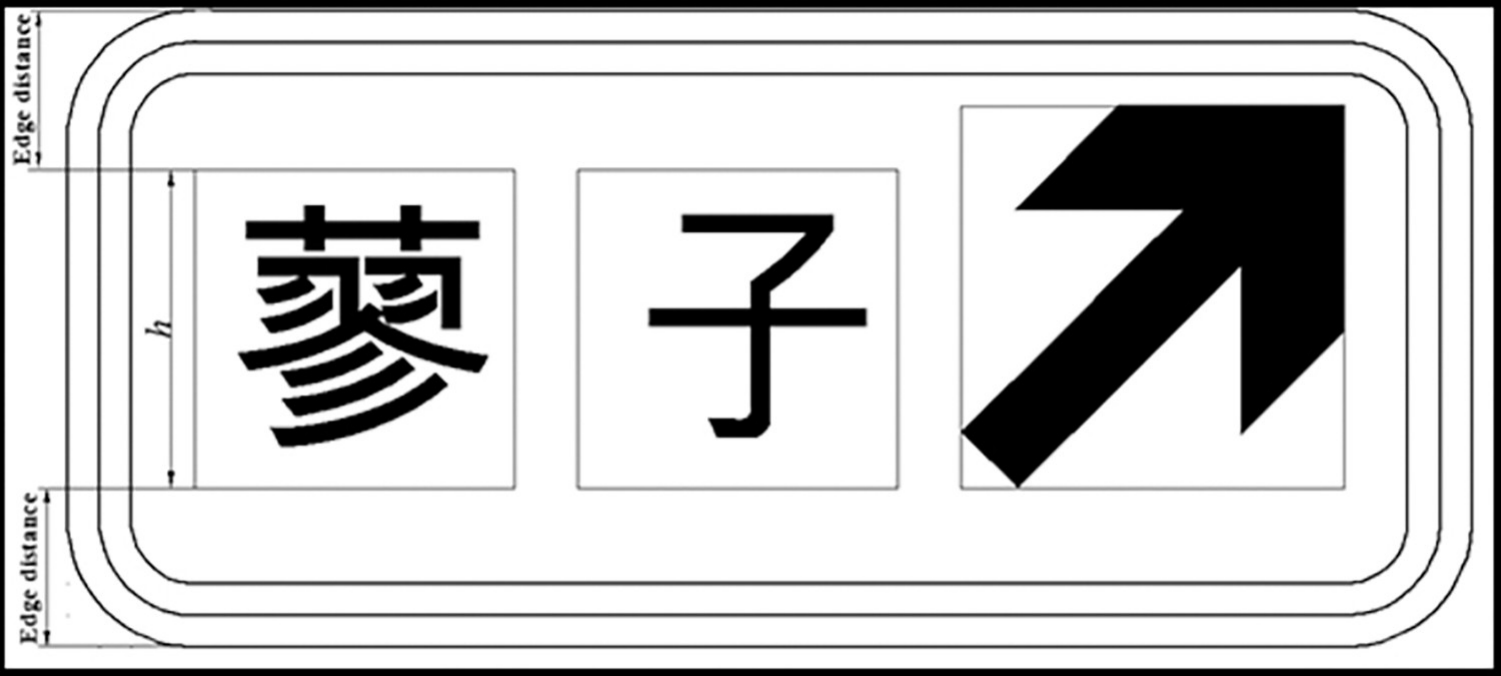
Layout of single-row text type exit advance guide sign.

**Fig 5:**
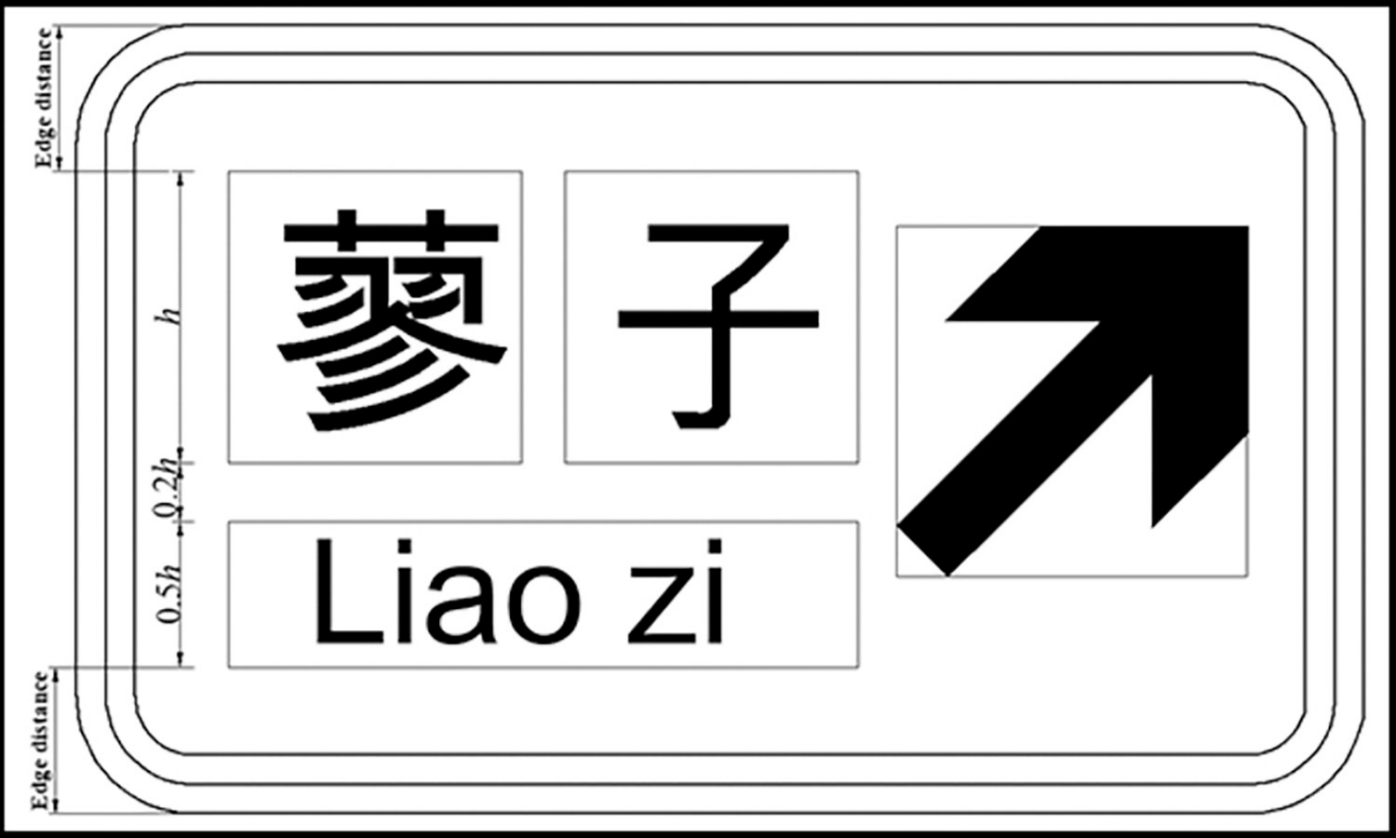
Layout of single row text with English place name type exit advance guide sign.

In this paper, “single-line text” and “single line text with English letter type” exit advance guide signs are used to explore their maximum width at different heights. Figs 4 and 5 show the layout of exit advance guide signs, where the height of Chinese characters is h and pinyin letters are taken as 0.5 h, with line spacing of 0.2 h. According to Formula 2, the maximum layout width L of an exit advance guide sign that can be set in a tunnel is shown in Table 2.

**Table 2:** Maximum layout width of exit warning signs under different constraints.

height from edge Character height	"Single row text" type			"Single line text with English place name" type		
	50	55	60	50	55	60
$\frac{2}{5}h$	699.33	676.54	652.45	602.89	562.36	517.47
$\frac{1}{2}h$	673.93	646.91	618.07	570.79	523.12	469.11
$\frac{3}{5}h$	646.91	615.08	580.68	536.02	479.69	413.76
$\frac{3}{4}h$	602.89	562.36	517.47	477.59	403.66	309.43

### 3.4 Calculation method of layout information of exit advance guide signs

Shannon [39], the founder of information theory, defined information as “an uncertain description of the state of motion or existence of things,” and mathematically defined the amount of information as the logarithm of the occurrence probability of random events with negative values,

$$H_{z}(x_{i})=-\log_{a}^{p(x_{i})}$$ (3)

where *x*_*i*_ is the i-th event; *a* is the base of the logarithm, generally taken as 2; *p*(*x*_*i*_) is the probability of the i-th event acting on people, 0≤*p*(*x*_*i*_)≤1, where $\sum_{i=1}^{n}p(x_{i})=1$; and *H*_*z*_(*x*_*i*_) is the amount of information of x_*i*_.

According to information theory, Hu [16] defined the information transmitted by road traffic facilities as “the description of the content of the attributes of the elements of road traffic facilities or the content expressed by their existence mode”. The information provided to highway users can be expressed as a sum,

$$H(X)=-\sum_{i=1}^{m}P(X_{i}{)\log}_{2}P(X_{i})$$ (4)

where *H*(*X*) is the amount of information of traffic engineering facility X, bits; m is the total number of possible events; *X*_*i*_ represents the state of element X; and *P*(*X*_*i*_) is the probability of occurrence of the i-th state.

Assuming that each state will occur, and each has the same probability, i.e., *P*(*X*_*i*_) = 1/m, the formula of the information amount of traffic engineering facilities can be simplified to

$$H_{i}(X)=\log_{2}m$$ (5)

where *H*_*i*_(*X*) is the amount of information of element i of traffic engineering facilities, in bits.

The layout of a tunnel exit notice sign consists mainly of colors, Chinese characters, pinyin letters, guide arrows, numbers, and geometric shapes. The three main colors are green, white, and red; there are about 3500 Chinese characters and 26 English letters (assuming that the uppercase and lowercase letters convey the same information); there are two kinds of guiding arrows; there are 10 numbers and two shapes. Table 3 shows the amount of information contained by each element.

**Table 3:** Information contained by each element of highway exit advance guide signs.

Element	Color	Chinese character	English letter	Guide arrow	Number	Geometric shape
Amount of information (bits)	1.58	11.77	4.75	1.00	3.32	1.00

Each element plays a different role in transmitting information to the driver. Therefore, in the process of quantifying the information amount of the exit advance guide sign, a weight should be assigned to each element. In this paper, the group synthetic construction method of a judgment matrix [44] is adopted to assign weights to elements. Thirteen traffic engineering practitioners were selected to compare the six elements in Table 2, in pairs, according to the scoring rules, and assign a_*ij*_ to form judgment matrix A_*k*_. The weights of the six components were calculated by geometric averaging for the judgment matrix, a consistency test was carried out on the results, and unqualified data were eliminated. Then the judgment weight P_k_ of each expert was calculated according to the assignment. The multi-expert comprehensive judgment matrix was used to calculate the weight of each element and carry out the consistency test.

Assuming that the maximum eigenvalue corresponding to the judgment matrix given by the k-th expert is $\lambda_{\max}^{\left(k\right)}$, we calculate the consistency index of the judgment matrix A_k_ by the method of the consistency test,

$$\mu_{k}=\frac{\lambda_{\max}^{\left(k\right)}-n}{n-1}\;(k=1,2,\cdots ,13)$$ (6)

where n is the order of the judgment matrix.

The weight of the expert’s judgment is

$$P_{k}=\frac{e^{-10(m-1)\mu_{k}}}{\sum_{i=1}^{13}e^{-10(m-1)\mu_{k}}}$$ (7)

where $\sum_{i=1}^{13}P_{k}=1$.

Therefore, after normalizing the judgment weights, the comprehensive judgment matrix A* is shown in Table 1, and it consists of elements

$$a_{ij}^{*}=\sum_{i=1}^{13}P_{k}a_{ij}^{\left(k\right)}$$ (8)

Where $a_{ij}^{*}$ is the element of comprehensive judgment matrix A*.

The comprehensive judgment matrix calculated according to the formula is shown in Table 4.

**Table 4:** Judgment matrix.

	color	Chinese character	letter	guide arrow	number	geometric shape
color	1.000	0.199	0.961	0.321	0.243	1.009
Chinese character	5.023	1.000	4.111	2.019	2.019	8.768
letter	1.041	0.243	1.000	0.335	0.351	2.897
guide arrow	3.115	0.495	2.982	1.000	1.053	3.010
number	4.120	0.495	2.849	0.950	1.000	2.959
geometric shape	0.991	0.114	0.345	0.332	0.338	1.000

We use the geometric average method to get the maximum eigenvalue of the matrix, $\lambda_{\max}^{\left(*\right)}=6.135$, *Coincidence Indicator* (*CI*) = 0.027, *Random Consistency Index* (*RI*) = 1.26 of the sixth-order matrix. Thus, the consistency test coefficient, *CR* = *CI*/*RI* = 0.021<0.1, satisfies the consistency test, and the weights of the constituent elements in the exit advance guide sign are shown in Table 5.

**Table 5:** Weight of each element of highway exit advance guide sign.

Element	color	Chinese character	letter	guide arrow	number	geometric shape
Weight	0.064	0.389	0.086	0.202	0.206	0.052

After considering the weight of each component in the exit advance guide sign, the effective amount of information of an exit advance guide sign in a highway tunnel, in bits, is

$$H(S)=n_{1}\varepsilon_{1}H_{1}(X)+\cdots +n_{6}\varepsilon_{6}H_{6}(X)$$ (9)

where *n*_*i*_ refers to the number of certain elements such as color, Chinese characters and letters in the exit advance guide sign; *ε*_*i*_ is the weight of element i; and *H*_*i*_(*X*) is the amount of information of element i of traffic engineering facilities, bits.

### 3.5 Layout setting of exit notice sign in tunnel

The layout width of an exit advance guide sign is negatively correlated with the height of the Chinese character and the distance between the text and the edge of the sign. According to the specification, the layout includes a place name, English place name, distance, guide arrow, highway number, “exit” text, and exit number. It is impossible for advance guide signs in tunnels to contain all these elements, so they need to be chosen.

We used the group comprehensive construction method of a judgment matrix to select 13 relevant traffic engineering experts to weigh the importance of the elements in exit advance guide signs, and formed a comprehensive judgment matrix, A*, as shown in Table 6.

**Table 6:** Judgment matrix.

	place name	English place name	distance	guide arrow	highway number	"exit"	exit number
place name	1.000	4.987	2.101	2.529	4.885	5.743	4.266
English place name	0.201	1.000	0.142	0.223	2.120	2.138	1.599
distance	0.476	7.053	1.000	1.204	3.852	5.109	3.540
guide arrow	0.395	4.493	0.831	1.000	2.643	3.085	2.613
highway number	0.205	0.472	0.260	0.378	1.000	0.861	0.270
"exit"	0.174	0.468	0.196	0.324	1.161	1.000	0.281
exit number	0.234	0.625	0.282	0.383	3.704	3.559	1.000

We used the geometric average method to get the maximum eigenvalue of the matrix, $\lambda_{\max}^{\left(*\right)}=9.301$, *CI* = 0.081, and a matrix of order 7, *RI* = 1.36, so the consistency test coefficient *CR* = *CI*/*RI* = 0.059<0.1 meets the consistency test. The weight of each element is shown in Table 7.

**Table 7:** Weight of each element of highway exit advance guide sign.

Element	place name	English place name	distance	guide arrow	highway number	“exit”	exit number
Weight	0.340	0.069	0.241	0.176	0.045	0.043	0.086

According to Table 7, it can be obtained that the weights of components such as highway number and exit are relatively low, and place name has the largest weight, which means it is indispensable. Therefore, we constructed 12 types of exit advance guide signs, with different amounts of information, combined with the place name, distance, guide arrow, English place name, and exit number. At the same time, considering that Chinese place names consist mainly of two or three characters, while setting up the experiment, in addition to the amount of information, the number of place names was used as a variable to explore the influence rule of exit advance guide signs with different words on the total time of drivers’ observation, reading, and reaction. According to the requirements of *Road Traffic Signs and Markings* (GB5768-2009) and *The Technical Guide for Adjustment of Traffic Signs in National Highway Network* (Ministry of Transport, 2017), 12 categories of advance guide signs were drawn. The height h of Chinese characters was 55 cm, the height of characters from the edge of the sign was 1/2 h, and the word interval was 1/10 h. Each type of exit advance guide sign had six-, two-, and three-character place names, for a total of 144 signs. The place names were all from the mountainous areas in China and contained no uncommon characters. To maintain the same amount of information in different sign combinations, place names with the same number of letters were selected (e.g., “Shui Jiang” and “Yong chuan” are both composed of two Chinese characters and nine letters). For signs with an exit distance, 500 m was chosen. Exit numbers on signs were randomly selected from the national highway network. The layout form and information content of signs with different combinations of elements are shown in S1 File. (Since the content of S1 File is larger than one A4 paper, it is placed in Annex 1)

### 3.6 Simulation experiment

Studies have shown that drivers’ recognition time of road signs is an important indicator to evaluate the threshold of traffic sign information [45]. We constructed exit advance guide signs with different combinations of elements and built a simulation scene with UC-win/Road. The E-prime simulation platform was used to explore the influence rules of drivers’ total time in the process of observing, reading, and responding to exit advance guide signs with different amounts of information, as well as their effect on drivers’ psychological loads.

**Fig 6:**
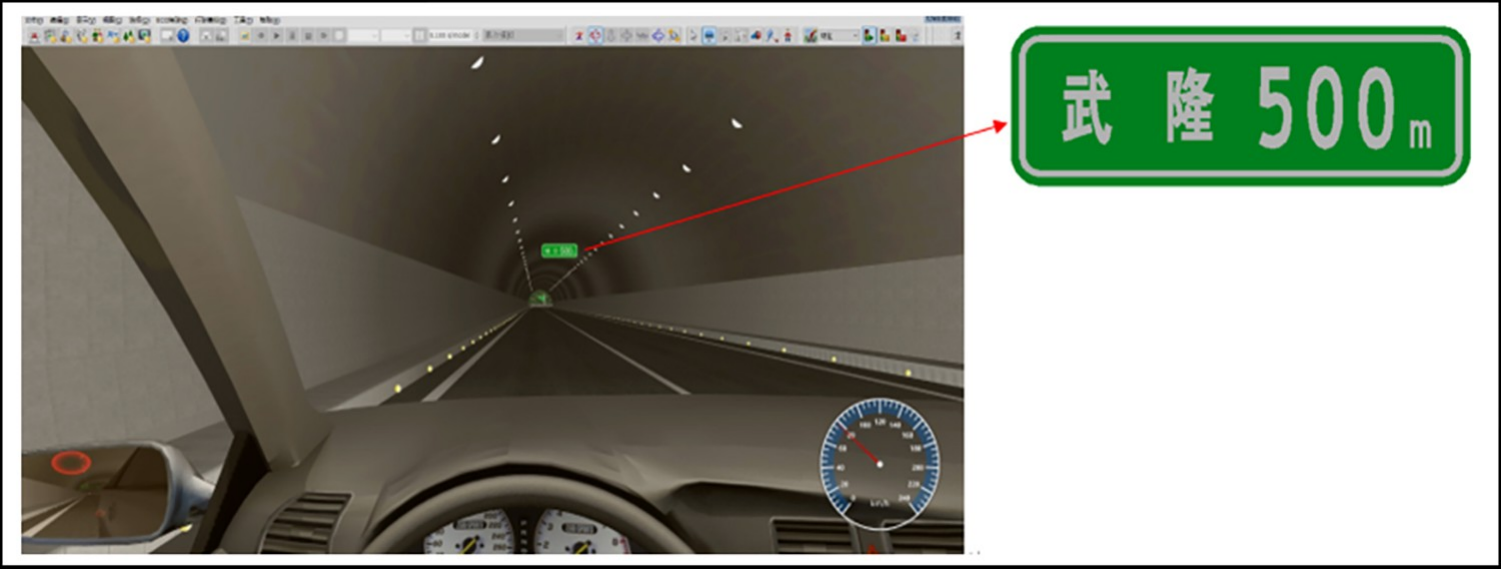
Driving simulation scenario.

A one-way, two-lane highway tunnel and an interchange model with a design speed of 80 km/h were built. The tunnel was 1532 m long, and the distance from the tunnel exit to the starting point of the gradual reduction lane of the interchange was 300 m. An exit advance guide sign was added to the model library and installed 200 m from the tunnel exit. The setting is shown in Fig 6.

**Fig 7:**
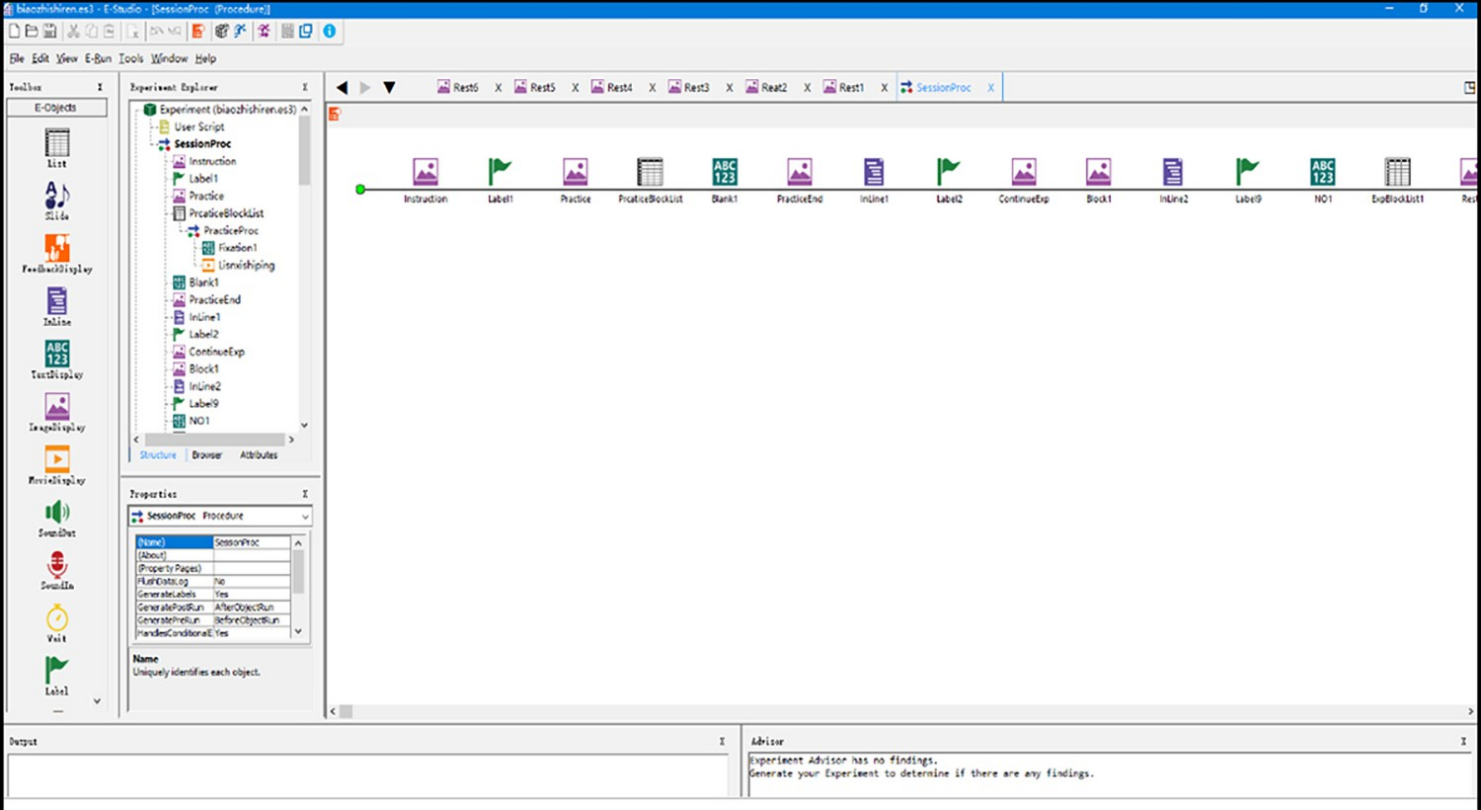
E-Prime simulation software interface and experimental flowchart.

We used E-Prime 3.0, an integrated experimental software system for psychological behavior research, to conduct experiments on the influence of exit advance guide signs on drivers’ driving load. We used Camtasia 9 software to convert the simulation model to a video with 1920 × 1080 resolution and a frame rate of 15 frames/s and imported 138 export advance guide signs with different combinations of elements into UC-win/Road. Research has shown that, at a running speed of 80 km/h, the maximum recognition distance of a traffic sign with a reading height of 55 cm is 123.24 m, and the recognition time is 5546 ms. Therefore, when making the video, the vehicle drove at a constant speed of 80 km/s from 123.34 m in front of the exit advance guide sign in the left or right lanes and ended after passing the sign. The length of each video after editing was 5546 ms. Experimental process was written in E-Prime 3.0 through the Procedure process timeline, as shown in Fig 7.

As shown in Table 7, the layout width of the three-character place name in combination 12 is greater than the maximum width of the specified layout, which does not meet the building boundary, and the rest of the exit advance guide signs meet the clearance requirements. Therefore, there were 138 stimulation videos in 12 combinations in this experiment. To avoid the effect of familiarity, it was divided into seven modules. The first module was a practice experiment, in which subjects practiced and mastered the relevant operations. Due to a large number of experimental stimulation videos, the non-randomness of the sequence of the same combination type of stimulus videos was avoided to affect the experimental results. Modules 2–7 were formal experiments, and each contained 1–8 and 10–12 combinations of two-character place names and three-character place name exit advance guide signs, respectively. The stimulation video and the combination 9 is a two-character exit trailer sign stimulus video, and each module contains a total of 23 stimulus videos and is presented randomly.

A total of 38 subjects were recruited to participate in the experiment, consisting of 65.8% males and 34.2% females, 57.9% with driving experience, and 42.1% with no driving experience. We obtained the written consent of 38 experimenters and used their answers for scientific research. Subjects were presented with a fixation point in the center of the screen for 1000 ms, followed by a random 5546-ms stimulus video, during which they were required to recognize all the information in the traffic signs as soon as possible. After reading all the information on the traffic signs, they quickly clicked the corresponding button on the keyboard. After responding, the screen presented the fixation point again for 1000 ms, and the next stimulus video appeared. If the subject did not respond within 5546 ms, the video would disappear and the next video would be presented at random. After completing a module, subjects were required to rest for 120 s, and they were required to complete all six modules. After completing the experiment, an assistant asked each subject to complete a subjective workload assessment technique (SWAT) questionnaire and an evaluation questionnaire.

### 3.7 Experimental data processing

Since it takes 211 ms for the human body to receive external stimuli to make a relevant response [46], 211 ms was subtracted to obtain the subject’s recognition and reaction times. The Kolmogorov-Smirnov (KS) test was used to test the hypothesis of the reaction time. The results showed that the experimental data obeyed a normal distribution. The absolute value of the residual error of the experimental data was more than three times the standard deviation, which meets the application conditions of the Pauta criterion.

### 3.8 Evaluation of the effect of setting exit advance guide signs

To analyze the psychological driving load of drivers when they recognize exit advance guide signs with different information combinations, as well as the intuitive feeling during the loading process after the drivers completed the E-prime simulation experiment, they filled out the SWAT scale and the exit advance guide signs comprehensive evaluation questionnaire. Evaluate the effectiveness of export notice sign layout loading with different information combinations. SWAT is a scale used for the evaluation of subjective psychological loads [47]. We comprehensively evaluated exit advance guide signs from the dimensions of recognition, amount of information, accuracy, and safety. The evaluation was carried out using the Likert five-point scale.

## 4. Results and discussion

### 4.1 Analysis of drivers’ reaction time to signs with different information combinations

The recognition reaction time is an important indicator to reflect the driver’s load level. The cognitive load and recognition characteristics of different information quantity exit beacons in the tunnel are mainly reflected through the recognition reaction time. Relevant research showed that [48,49], the recognition time of drivers for traffic signs is an important indicator to evaluate the threshold value of traffic sign information, so recognition reaction time is selected to evaluate the level of drivers’ visual load.

To explore whether the driver’s gender, driving experience, driving in different lanes, and different amounts of information have a significant impact on a driver’s recognition reaction time, a significance analysis was carried out using a one-way variance. The statistical results of two and three-character names are shown in Tables 8 and 9, respectively.

**Table 8:** Recognition reaction time of exit advance guide signs.

Type	Source	Elements	Average reaction time	Root-mean-square error
two-character place names	Gender	Males	4254.83	518.491
		Female	4193.11	518.082
	Driving experience	Posses	4181.56	470.725
		Do not posses	4274.04	555.244
	Driving lane	Left lane	4209.25	511.54
		Right lane	4251.36	525.814
three-character place names	Gender	Males	4157.96	481.849
		Female	4151.9	488.622
	Driving experience	Posses	4064.7	510.947
		Do not posses	4206.68	461.503
	Driving lane	Left lane	4158.33	468.959
		Right lane	4152.37	500.07

**Table 9:** Single-factor analysis of variance results of drivers’ recognition reaction time of exit warning signs.

Type	Source	*d/f*	*F*	*p*
two-character place names	Gender	1	4.875	0.027
	Driving experience	1	11.465	0.001
	Driving lane	1	2.37	0.124
three-character place names	Gender	1	4.875	0.027
	Driving experience	1	11.465	0.001
	Driving lane	1	2.37	0.124

**Fig 8:**
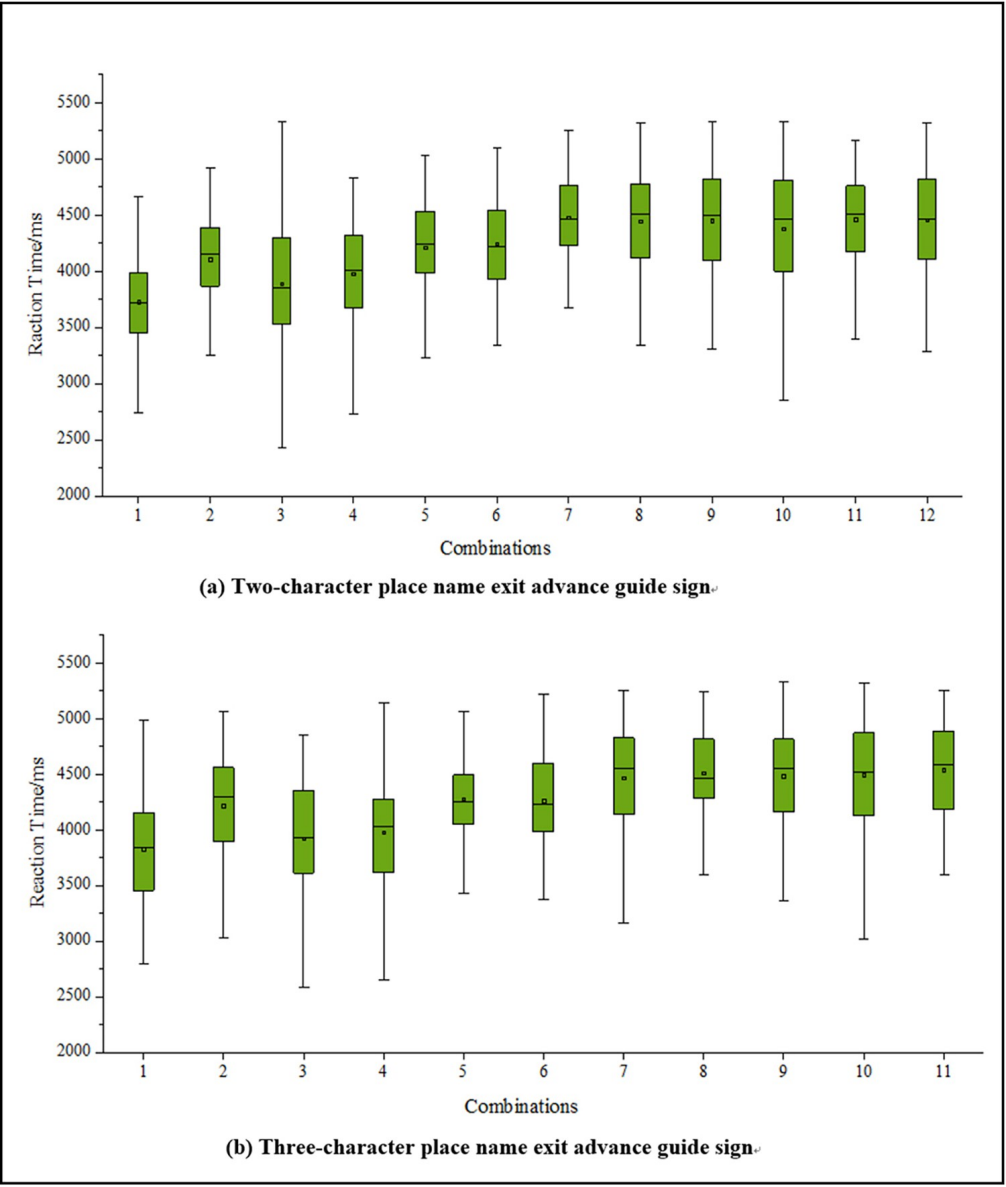
The average reaction time of drivers’ recognition of exit advance guide signs under different information combinations.

According to the statistical results in Tables 8 and 9, the driver’s gender and driving lane have no significant influence on recognition reaction times, and driving experience has a significant influence. Those with driving experience have shorter recognition reaction times. After excluding the above irrelevant factors, the single factor analysis of variance shows that exit advance guide signs with different amounts of information have significant differences in the recognition reaction time of drivers. The average reaction time of drivers’ recognition of exit advance guide signs under different information combinations is shown in Table 10, and the distribution of recognition reaction times is shown in Fig 8.

**Table 10:** Average recognition reaction time of drivers under different information combinations.

Combination	"Two character" exit advance guide sign			"Three character" exit advance guide sign		
	Information Quantity /bits	Recognition reaction time /ms	Root-mean-square error	Information Quantity /bits	Recognition reaction time /ms	Root-mean-square error
1	9.613	3731.88	501.233	14.192	3840.22	478.910
2	11.033	4118.40	350.628	15.612	4057.81	448.358
3	11.872	3887.13	520.734	16.450	3825.57	504.392
4	12.074	3983.85	460.210	16.652	3869.93	460.193
5	13.290	4435.33	416.858	17.870	4193.02	340.135
6	13.291	4213.99	393.589	18.072	4118.35	377.094
7	13.493	4243.78	407.139	18.685	4329.09	446.579
8	14.710	4432.12	471.506	20.105	4317.94	452.860
9	15.548	4451.71	484.110	20.944	4347.18	448.497
10	15.750	4378.62	586.566	21.146	4304.07	463.208
11	16.968	4448.71	424.801	22.363	4412.13	403.419
12	17.170	4441.99	489.410	-	-	-

From Fig 8 and Table 10, it can be seen that the recognition time of a driver of an exit advance guide sign does not increase with the amount of information, and is related to the layout content setting. The recognition reaction time of signs containing English geographic names is greater than that of those without them, indicating a larger driving load. Among signs without English place names, the average recognition reaction time of drivers to recognize signs with two-character place names and three-character place names, from small to large, were ranked by combination as 1 < 3 < 4 < 2 < 6 < 5. Among signs with English place names, the average reaction time to recognize signs with two-character place names was ranked by combination as 10 < 8 < 9 < 12 < 11 < 7, and for three-character place names, as 8< 7 < 10 < 9 < 11.

### 4.2 Effectiveness analysis of layout loading for exit advance guide signs with different information combinations

To ensure the reliability of the SWAT scale and driver comprehensive evaluation questionnaire, the reliability and validity of the scale were tested. Cronbach’s coefficient was used to test the consistency of the questionnaire. A value greater than 0.6 shows that the reliability of the questionnaire meets requirements; a validity test, KMO test, and Bartlett spherical test were used to determine whether the four dimensions of observation variables had structural validity. The reliability test showed that the overall reliability of SWAT was 0.626, and the Cronbach coefficients of the time, psychological effort, and psychological tension loads were greater than 0.6. The overall reliability of the questionnaire for the comprehensive evaluation of drivers was 0.841, and the Cronbach coefficients of sign recognition, information, accuracy, and safety were greater than 0.79, indicating that the reliability of the survey results met the basic requirements. The validity test results showed that the KMO test result of the SWAT scale was 0.622, and the KMO test result of the comprehensive evaluation questionnaire was 0.792, which was greater than 0.5; moreover, the Bartlett spherical test result was less than 0.001, validating the SWAT scale and comprehensive evaluation questionnaire.

**Fig 9:**
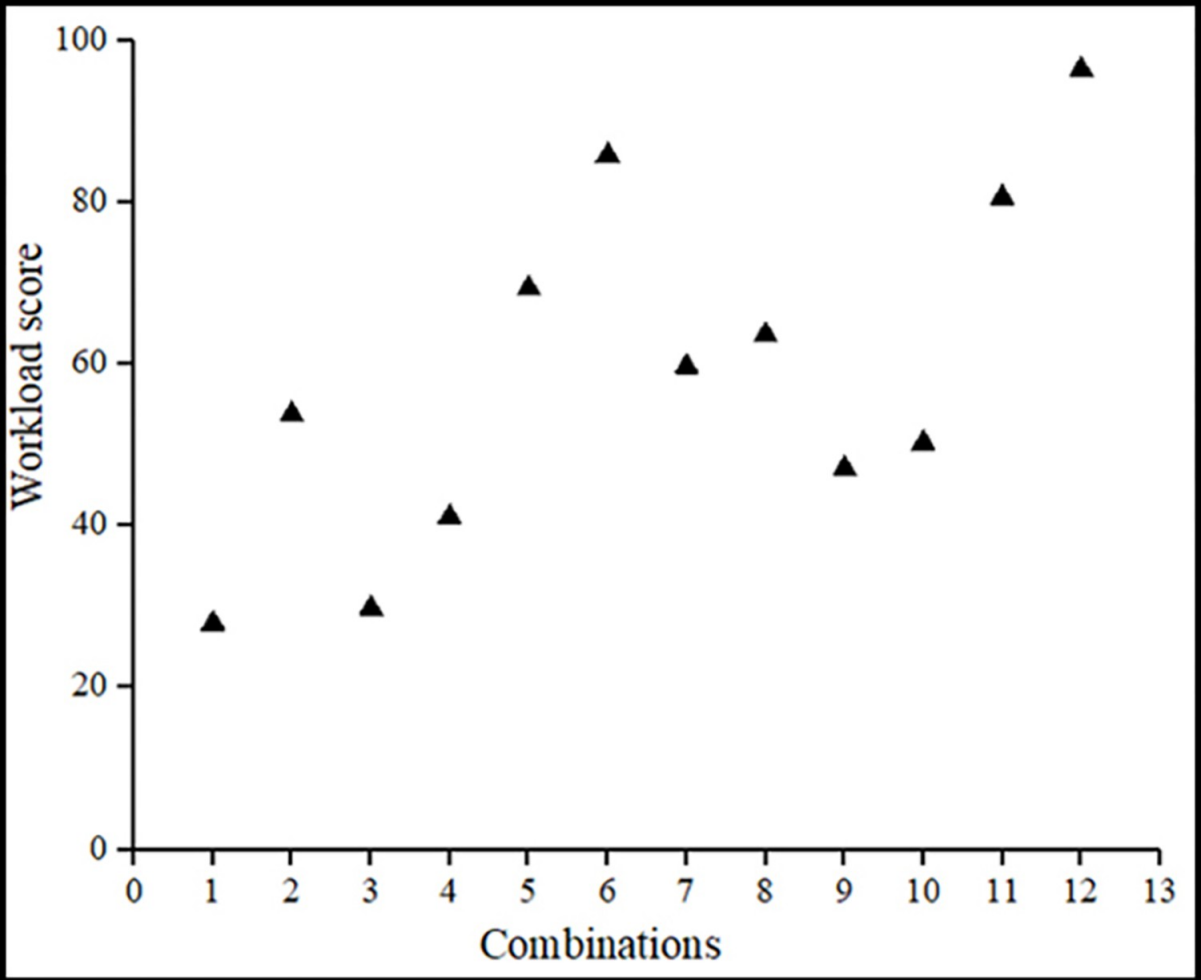
Driver’s subjective workload score under exit advance guide signs with different information combinations.

According to the total score, the subjective psychological load of 38 test drivers on 12 information combinations of exit advance guide signs was converted to a scale of 0–100 points, and the average value was taken, as shown in Fig 9.

According to Fig 9, the driver’s subjective workload score was higher for signs without English place names than for those with English place names. For those without English names, the workload scores were ranked by combination as 1 < 3 < 4 < 2 < 5 < 6. Those with English names, from small to large, were ranked by combination as 9 < 10 < 7 < 8 < 11 < 12.

The average score of drivers’ subjective evaluation of 12 different information combinations of exit advance guide signs is shown in Table 11.

**Table 11:** Comprehensive evaluation score of exit advance guide signs in different information combinations.

组合	标志识认性	标志信息量	标志准确性	标志安全性
1	1.79	2.29	2.89	2.47
2	2.58	2.71	2.81	2.63
3	2.16	2.66	2.45	2.34
4	4.18	4.37	4.45	4.47
5	3.24	3.18	3.08	2.76
6	4.05	3.97	3.82	3.81
7	2.66	2.92	2.89	2.97
8	2.55	2.79	3.05	2.92
9	2.95	2.87	2.53	2.39
10	4.08	4.37	4.47	4.32
11	3.02	3.21	3.31	3.13
12	4.05	3.82	3.92	3.76

The average scores of drivers’ subjective evaluations of exit advance guide signs with 12 different information combinations are shown in Table 11, according to which, in the dimension of sign recognition evaluation, the comprehensive evaluation scores of 12 kinds of exit advance guide signs are mainly distributed between 1.79 and 4.18. The recognition scores of signs without English names were ranked by combination as 4 > 6 > 5 > 2 > 3 > 1, and with English geographic names as 10 > 12 > 11 > 9 > 7 > 8.

In the dimension of information content evaluation, the comprehensive evaluation scores of 12 exit advance guide signs were distributed between 2.29 and 4.37. The recognition scores of signs without English geographic names were ranked by combination as 4 > 6 > 5 > 2 > 3 > 1, and those with English geographic names as 10 > 12 > 11 > 7 > 9 > 8.

In the accuracy evaluation dimension of sign information, the evaluation scores of 12 exit advance guide signs were between 2.25 and 4.47. The recognition scores of signs without English names were ranked by combination as 4 > 6 > 5 > 1> 2 > 3, and those with English names as 10 > 12 > 11 > 8 > 7 > 9.

In the dimension of sign safety evaluation, the comprehensive evaluation scores of 12 exit advance guide signs are mainly distributed between 2.34 and 4.47; The recognition scores of signs without English place names were ranked by combination as 4 > 6 > 5> 2> 1> 3, and those with English names as 10> 12>combination 11> 7> 8> 9.

**Fig 10:**
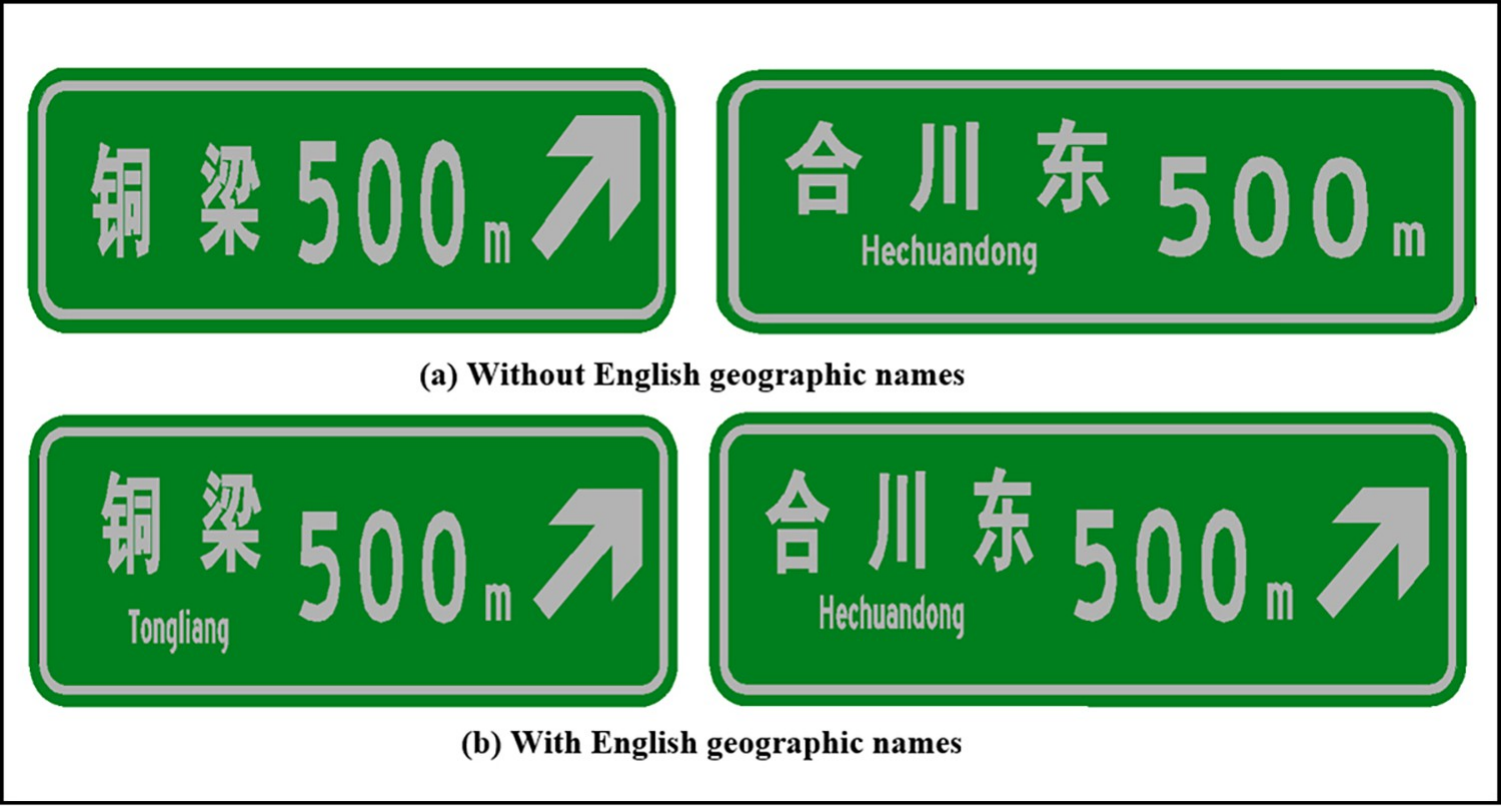
Sample layout of exit advance guide signs in tunnel.

From the aspects of driver’s subjective workload, sign recognition, amount of sign information, sign accuracy, and sign safety, it can be seen that combinations 4 and 10 had good results in the loading experiment. The recognition time of these two kinds of exit advance guide signs was relatively small compared with that of other combinations, as shown in Fig 10. Therefore, it is suggested that exit advance guide signs in tunnels should have the form of “place name + distance + guide arrow” (combination 4) as shown in Fig 10(A). If travelers in the project area have a high demand for English place names, signs can be set as shown in Fig 10(B), i.e., “place name + English place name + distance + guide arrow” (combination 10), where English place names adopt pinyin, the English initial letter is capitalized, and the rest are lowercase.

## 5. Conclusion

The main observations of this experiment are revealed as follows. The group comprehensive construction method of a judgment matrix was used to weight the components of expressway exit advance guide signs. The results showed that the weights of place name, distance, guide arrow, English place name, and exit number were relatively high. The width of the exit advance guide sign is negatively correlated with the height of Chinese characters and the distance between the characters and the edge of the sign. The greater the height of Chinese characters and the greater the distance between characters and the edges of signs, the smaller the maximum layout width of exit advance guide signs. Based on information quantification theory, 12 kinds of exit advance guide signs with different information combinations were proposed, and the recognition response times of test drivers to the 12 combinations were collected in an E-Prime simulation experiment. Based on the subjective workload and comprehensive evaluation scores of different drivers, we proposed that the layout of exit advance guide signs in tunnels should be set in the form of Chinese/English place names + distance + guide arrows. The layout design method of exit advance guide sign proposed in this paper is also applicable to similar tunnels. In order to ensure traffic safety, the plane alignment of long tunnels, medium tunnels and short tunnels on the highway mostly adopts straight lines or curves with larger radius, so the road alignment in the tunnel area is better. The tunnel road alignment contains straight lines and horizontal curves. In this study, in order to control the number of variables and reduce the impact of variables on the driver’s sign recognition process, only straight sections were selected as the research object. Due to the limited length of the paper, the impact of different horizontal and vertical alignment combinations on driver recognition signs can be considered in the subsequent research.

## Supporting information

S1 Table Summary of data of two-character place names test. (PDF)

S2 Table Summary of data of three-character place names test. (PDF)

S3 Table Reaction time of two-character place names. (PDF)

S4 Table Reaction time of three-character place names. (PDF)

S5 Table Informed Consent (Chinese). (PDF)

S6 Table Informed Consent (English). (PDF)

S1 File Layout form and information content of exit warning signs with different combinations of elements. (DOCX)
