# Supplementary material for: Setting method of exit advance guide signs in mountainous expressway tunnel based on information quantization theory
Source: PLoS One. 2023 Feb 16;18(2):e0281842. doi: 10.1371/journal.pone.0281842 (PMC9934451; doi:10.1371/journal.pone.0281842)
Supplement: S1 Table — (PDF) [file pone.0281842.s001.pdf]

Summary of statistical data of two-character place names test

number	Age	Gender	experience	Lane number	combination	amount of information	reaction time
1	18-30	male	Yes	Left lane	3	11.872	2716
2	18-30	male	Yes	Left lane	4	12.074	3017
3	18-30	male	Yes	Right lane	10	15.75	3056
4	18-30	male	Yes	Left lane	3	11.872	3106
5	18-30	male	Yes	Right lane	3	11.872	3157
6	18-30	male	Yes	Left lane	1	9.613	3161
7	18-30	male	Yes	Left lane	4	12.074	3183
8	18-30	male	Yes	Left lane	1	9.613	3305
9	18-30	male	Yes	Left lane	8	14.71	3407
10	18-30	male	Yes	Right lane	4	12.074	3412
11	18-30	male	Yes	Left lane	5	13.291	3473
12	18-30	male	Yes	Right lane	2	11.033	3528
13	18-30	male	Yes	Left lane	2	11.033	3597
14	18-30	male	Yes	Right lane	10	15.75	3614
15	18-30	male	Yes	Right lane	4	12.074	3614
16	18-30	male	Yes	Right lane	1	9.613	3632
17	18-30	male	Yes	Left lane	10	15.75	3650
18	18-30	male	Yes	Right lane	5	13.291	3736
19	18-30	male	Yes	Left lane	7	13.29	3738
20	18-30	male	Yes	Left lane	9	15.548	3759
21	18-30	male	Yes	Right lane	6	13.493	3760
22	18-30	male	Yes	Left lane	4	12.074	3787
23	18-30	male	Yes	Right lane	12	17.17	3877
24	18-30	male	Yes	Right lane	1	9.613	3881
25	18-30	male	Yes	Right lane	2	11.033	3907
26	18-30	male	Yes	Right lane	1	9.613	3907
27	18-30	male	Yes	Left lane	6	13.493	3912
28	18-30	male	Yes	Right lane	7	13.29	3984
29	18-30	male	Yes	Left lane	5	13.291	4000
30	18-30	male	Yes	Right lane	6	13.493	4024
31	18-30	male	Yes	Left lane	2	11.033	4035
32	18-30	male	Yes	Left lane	1	9.613	4058
33	18-30	male	Yes	Right lane	5	13.291	4067
34	18-30	male	Yes	Right lane	6	13.493	4069
35	18-30	male	Yes	Left lane	2	11.033	4164
36	18-30	male	Yes	Right lane	10	15.75	4182
37	18-30	male	Yes	Left lane	11	16.968	4192
38	18-30	male	Yes	Right lane	9	15.548	4194
39	18-30	male	Yes	Right lane	5	13.291	4195
40	18-30	male	Yes	Left lane	3	11.872	4225
41	18-30	male	Yes	Left lane	12	17.17	4273
42	18-30	male	Yes	Left lane	8	14.71	4277
43	18-30	male	Yes	Right lane	2	11.033	4306
44	18-30	male	Yes	Right lane	7	13.29	4358
45	18-30	male	Yes	Left lane	3	11.872	4374
46	18-30	male	Yes	Right lane	8	14.71	4393
47	18-30	male	Yes	Left lane	12	17.17	4424
48	18-30	male	Yes	Left lane	9	15.548	4448
49	18-30	male	Yes	Left lane	4	12.074	4462
50	18-30	male	Yes	Right lane	12	17.17	4464
51	18-30	male	Yes	Right lane	3	11.872	4465

52	18-30	male	Yes	Left lane	10	15.75	4467
53	18-30	male	Yes	Left lane	8	14.71	4479
54	18-30	male	Yes	Left lane	12	17.17	4493
55	18-30	male	Yes	Right lane	4	12.074	4565
56	18-30	male	Yes	Right lane	8	14.71	4612
57	18-30	male	Yes	Right lane	10	15.75	4621
58	18-30	male	Yes	Right lane	2	11.033	4637
59	18-30	male	Yes	Right lane	7	13.29	4660
60	18-30	male	Yes	Right lane	11	16.968	4660
61	18-30	male	Yes	Right lane	11	16.968	4681
62	18-30	male	Yes	Left lane	6	13.493	4694
63	18-30	male	Yes	Right lane	7	13.29	4705
64	18-30	male	Yes	Left lane	11	16.968	4713
65	18-30	male	Yes	Right lane	9	15.548	4772
66	18-30	male	Yes	Right lane	9	15.548	4783
67	18-30	male	Yes	Left lane	7	13.29	4802
68	18-30	male	Yes	Right lane	11	16.968	4805
69	18-30	male	Yes	Right lane	9	15.548	4809
70	18-30	male	Yes	Left lane	11	16.968	4812
71	18-30	male	Yes	Left lane	7	13.29	4819
72	18-30	male	Yes	Right lane	5	13.291	4839
73	18-30	male	Yes	Left lane	5	13.291	4882
74	18-30	male	Yes	Right lane	12	17.17	4883
75	18-30	male	Yes	Left lane	9	15.548	4983
76	18-30	male	Yes	Right lane	8	14.71	4987
77	18-30	male	Yes	Right lane	8	14.71	5024
78	18-30	male	Yes	Left lane	10	15.75	5047
79	18-30	male	Yes	Left lane	6	13.493	5079
80	18-30	male	Yes	Left lane	12	17.17	5191
81	18-30	male	Yes	Right lane	3	11.872	5329
82	18-30	female	No	Right lane	10	15.75	2846
83	18-30	female	No	Right lane	7	13.29	3320
84	18-30	female	No	Left lane	10	15.75	3347
85	18-30	female	No	Left lane	4	12.074	3397
86	18-30	female	No	Left lane	11	16.968	3400
87	18-30	female	No	Left lane	5	13.291	3424
88	18-30	female	No	Left lane	3	11.872	3454
89	18-30	female	No	Right lane	4	12.074	3525
90	18-30	female	No	Right lane	2	11.033	3562
91	18-30	female	No	Left lane	3	11.872	3582
92	18-30	female	No	Right lane	1	9.613	3585
93	18-30	female	No	Right lane	2	11.033	3591
94	18-30	female	No	Right lane	10	15.75	3601
95	18-30	female	No	Right lane	4	12.074	3612
96	18-30	female	No	Left lane	2	11.033	3647
97	18-30	female	No	Right lane	3	11.872	3659
98	18-30	female	No	Right lane	5	13.291	3695
99	18-30	female	No	Left lane	1	9.613	3704
100	18-30	female	No	Right lane	3	11.872	3714
101	18-30	female	No	Left lane	1	9.613	3717
102	18-30	female	No	Right lane	6	13.493	3718
103	18-30	female	No	Left lane	10	15.75	3725
104	18-30	female	No	Right lane	4	12.074	3726
105	18-30	female	No	Right lane	1	9.613	3727

106	18-30	female	No	Left lane	4	12.074	3729
107	18-30	female	No	Left lane	12	17.17	3753
108	18-30	female	No	Left lane	8	14.71	3762
109	18-30	female	No	Right lane	10	15.75	3783
110	18-30	female	No	Left lane	6	13.493	3786
111	18-30	female	No	Left lane	1	9.613	3791
112	18-30	female	No	Right lane	5	13.291	3792
113	18-30	female	No	Left lane	9	15.548	3793
114	18-30	female	No	Right lane	1	9.613	3813
115	18-30	female	No	Right lane	8	14.71	3821
116	18-30	female	No	Right lane	6	13.493	3829
117	18-30	female	No	Left lane	2	11.033	3860
118	18-30	female	No	Left lane	10	15.75	3871
119	18-30	female	No	Right lane	9	15.548	3871
120	18-30	female	No	Right lane	2	11.033	3925
121	18-30	female	No	Right lane	12	17.17	3929
122	18-30	female	No	Left lane	2	11.033	3935
123	18-30	female	No	Left lane	12	17.17	3950
124	18-30	female	No	Right lane	11	16.968	3983
125	18-30	female	No	Left lane	6	13.493	3988
126	18-30	female	No	Right lane	8	14.71	4003
127	18-30	female	No	Right lane	7	13.29	4029
128	18-30	female	No	Right lane	3	11.872	4032
129	18-30	female	No	Left lane	6	13.493	4055
130	18-30	female	No	Left lane	8	14.71	4070
131	18-30	female	No	Left lane	7	13.29	4072
132	18-30	female	No	Right lane	12	17.17	4112
133	18-30	female	No	Right lane	9	15.548	4125
134	18-30	female	No	Left lane	8	14.71	4155
135	18-30	female	No	Right lane	11	16.968	4181
136	18-30	female	No	Right lane	5	13.291	4184
137	18-30	female	No	Right lane	6	13.493	4226
138	18-30	female	No	Left lane	5	13.291	4226
139	18-30	female	No	Left lane	11	16.968	4246
140	18-30	female	No	Left lane	9	15.548	4255
141	18-30	female	No	Left lane	7	13.29	4257
142	18-30	female	No	Left lane	12	17.17	4287
143	18-30	female	No	Right lane	12	17.17	4346
144	18-30	female	No	Right lane	7	13.29	4351
145	18-30	female	No	Left lane	7	13.29	4355
146	18-30	female	No	Left lane	5	13.291	4391
147	18-30	female	No	Right lane	11	16.968	4449
148	18-30	female	No	Left lane	4	12.074	4497
149	18-30	female	No	Left lane	3	11.872	4660
150	18-30	female	No	Right lane	8	14.71	4692
151	18-30	female	No	Left lane	11	16.968	4694
152	18-30	female	No	Left lane	9	15.548	4780
153	18-30	female	No	Right lane	9	15.548	4859
154	18-30	male	No	Right lane	12	17.17	3283
155	18-30	male	No	Right lane	9	15.548	3320
156	18-30	male	No	Right lane	4	12.074	3427
157	18-30	male	No	Right lane	1	9.613	3557
158	18-30	male	No	Right lane	3	11.872	3579
159	18-30	male	No	Left lane	3	11.872	3585

160	18-30	male	No	Left lane	2	11.033	3613
161	18-30	male	No	Left lane	1	9.613	3639
162	18-30	male	No	Left lane	2	11.033	3740
163	18-30	male	No	Left lane	7	13.29	3831
164	18-30	male	No	Right lane	1	9.613	3848
165	18-30	male	No	Left lane	2	11.033	3892
166	18-30	male	No	Left lane	5	13.291	3914
167	18-30	male	No	Left lane	10	15.75	3916
168	18-30	male	No	Left lane	9	15.548	3922
169	18-30	male	No	Right lane	12	17.17	3924
170	18-30	male	No	Left lane	12	17.17	3928
171	18-30	male	No	Right lane	11	16.968	3929
172	18-30	male	No	Right lane	10	15.75	3929
173	18-30	male	No	Right lane	2	11.033	3939
174	18-30	male	No	Left lane	9	15.548	3954
175	18-30	male	No	Right lane	6	13.493	3960
176	18-30	male	No	Left lane	5	13.291	3987
177	18-30	male	No	Right lane	6	13.493	4017
178	18-30	male	No	Left lane	4	12.074	4046
179	18-30	male	No	Right lane	4	12.074	4064
180	18-30	male	No	Right lane	11	16.968	4072
181	18-30	male	No	Right lane	8	14.71	4088
182	18-30	male	No	Left lane	6	13.493	4114
183	18-30	male	No	Right lane	6	13.493	4124
184	18-30	male	No	Left lane	5	13.291	4125
185	18-30	male	No	Left lane	3	11.872	4145
186	18-30	male	No	Right lane	2	11.033	4171
187	18-30	male	No	Right lane	5	13.291	4185
188	18-30	male	No	Right lane	10	15.75	4196
189	18-30	male	No	Left lane	11	16.968	4220
190	18-30	male	No	Left lane	8	14.71	4222
191	18-30	male	No	Left lane	1	9.613	4266
192	18-30	male	No	Left lane	9	15.548	4282
193	18-30	male	No	Right lane	7	13.29	4314
194	18-30	male	No	Right lane	10	15.75	4329
195	18-30	male	No	Left lane	6	13.493	4333
196	18-30	male	No	Right lane	3	11.872	4350
197	18-30	male	No	Right lane	2	11.033	4368
198	18-30	male	No	Right lane	9	15.548	4379
199	18-30	male	No	Left lane	12	17.17	4394
200	18-30	male	No	Left lane	7	13.29	4397
201	18-30	male	No	Right lane	4	12.074	4417
202	18-30	male	No	Right lane	3	11.872	4421
203	18-30	male	No	Left lane	4	12.074	4440
204	18-30	male	No	Right lane	12	17.17	4441
205	18-30	male	No	Left lane	11	16.968	4513
206	18-30	male	No	Left lane	3	11.872	4525
207	18-30	male	No	Left lane	4	12.074	4591
208	18-30	male	No	Right lane	7	13.29	4616
209	18-30	male	No	Right lane	5	13.291	4622
210	18-30	male	No	Right lane	8	14.71	4635
211	18-30	male	No	Right lane	11	16.968	4648
212	18-30	male	No	Right lane	5	13.291	4648
213	18-30	male	No	Left lane	10	15.75	4667

214	18-30	male	No	Left lane	8	14.71	4669
215	18-30	male	No	Right lane	8	14.71	4746
216	18-30	male	No	Left lane	11	16.968	4759
217	18-30	male	No	Left lane	8	14.71	4767
218	18-30	male	No	Left lane	6	13.493	4785
219	18-30	male	No	Right lane	9	15.548	4814
220	18-30	male	No	Left lane	1	9.613	4828
221	18-30	male	No	Left lane	12	17.17	4871
222	18-30	male	No	Right lane	1	9.613	4884
223	18-30	male	No	Right lane	7	13.29	4885
224	18-30	male	No	Left lane	7	13.29	4905
225	18-30	male	No	Right lane	10	15.75	3292
226	18-30	male	No	Right lane	1	9.613	3685
227	18-30	male	No	Left lane	3	11.872	3692
228	18-30	male	No	Right lane	10	15.75	3751
229	18-30	male	No	Left lane	3	11.872	3784
230	18-30	male	No	Left lane	8	14.71	3791
231	18-30	male	No	Right lane	5	13.291	3806
232	18-30	male	No	Left lane	1	9.613	3812
233	18-30	male	No	Left lane	2	11.033	3829
234	18-30	male	No	Right lane	12	17.17	3839
235	18-30	male	No	Left lane	1	9.613	3847
236	18-30	male	No	Left lane	3	11.872	3854
237	18-30	male	No	Right lane	3	11.872	3857
238	18-30	male	No	Left lane	4	12.074	3895
239	18-30	male	No	Right lane	9	15.548	3948
240	18-30	male	No	Right lane	5	13.291	3960
241	18-30	male	No	Right lane	1	9.613	3961
242	18-30	male	No	Left lane	4	12.074	3965
243	18-30	male	No	Right lane	2	11.033	3986
244	18-30	male	No	Left lane	11	16.968	3986
245	18-30	male	No	Right lane	1	9.613	4010
246	18-30	male	No	Left lane	6	13.493	4022
247	18-30	male	No	Left lane	12	17.17	4038
248	18-30	male	No	Left lane	6	13.493	4055
249	18-30	male	No	Right lane	6	13.493	4087
250	18-30	male	No	Left lane	8	14.71	4104
251	18-30	male	No	Left lane	12	17.17	4125
252	18-30	male	No	Right lane	7	13.29	4131
253	18-30	male	No	Left lane	4	12.074	4147
254	18-30	male	No	Right lane	2	11.033	4153
255	18-30	male	No	Right lane	4	12.074	4185
256	18-30	male	No	Right lane	4	12.074	4198
257	18-30	male	No	Left lane	5	13.291	4204
258	18-30	male	No	Right lane	4	12.074	4232
259	18-30	male	No	Left lane	2	11.033	4236
260	18-30	male	No	Right lane	2	11.033	4246
261	18-30	male	No	Left lane	11	16.968	4247
262	18-30	male	No	Left lane	5	13.291	4258
263	18-30	male	No	Left lane	12	17.17	4258
264	18-30	male	No	Right lane	8	14.71	4262
265	18-30	male	No	Right lane	3	11.872	4272
266	18-30	male	No	Left lane	9	15.548	4332
267	18-30	male	No	Left lane	6	13.493	4339

268	18-30	male	No	Left lane	10	15.75	4361
269	18-30	male	No	Right lane	8	14.71	4364
270	18-30	male	No	Left lane	9	15.548	4403
271	18-30	male	No	Left lane	2	11.033	4405
272	18-30	male	No	Right lane	11	16.968	4406
273	18-30	male	No	Left lane	5	13.291	4439
274	18-30	male	No	Right lane	3	11.872	4459
275	18-30	male	No	Left lane	7	13.29	4460
276	18-30	male	No	Left lane	7	13.29	4479
277	18-30	male	No	Right lane	12	17.17	4494
278	18-30	male	No	Left lane	11	16.968	4502
279	18-30	male	No	Right lane	8	14.71	4510
280	18-30	male	No	Right lane	11	16.968	4510
281	18-30	male	No	Left lane	10	15.75	4535
282	18-30	male	No	Right lane	6	13.493	4544
283	18-30	male	No	Right lane	11	16.968	4570
284	18-30	male	No	Left lane	7	13.29	4577
285	18-30	male	No	Right lane	7	13.29	4606
286	18-30	male	No	Right lane	9	15.548	4606
287	18-30	male	No	Right lane	12	17.17	4635
288	18-30	male	No	Left lane	9	15.548	4649
289	18-30	male	No	Right lane	5	13.291	4655
290	18-30	male	No	Left lane	1	9.613	4660
291	18-30	male	No	Right lane	6	13.493	4699
292	18-30	male	No	Right lane	9	15.548	4715
293	18-30	male	No	Right lane	10	15.75	4716
294	18-30	male	No	Left lane	8	14.71	4791
295	18-30	male	No	Left lane	10	15.75	4827
296	18-30	male	No	Right lane	7	13.29	5054
297	18-30	female	Yes	Left lane	4	12.074	2796
298	18-30	female	Yes	Left lane	8	14.71	2847
299	18-30	female	Yes	Left lane	4	12.074	2958
300	18-30	female	Yes	Left lane	3	11.872	3152
301	18-30	female	Yes	Right lane	12	17.17	3468
302	18-30	female	Yes	Right lane	3	11.872	3482
303	18-30	female	Yes	Right lane	3	11.872	3482
304	18-30	female	Yes	Right lane	3	11.872	3516
305	18-30	female	Yes	Left lane	2	11.033	3581
306	18-30	female	Yes	Right lane	1	9.613	3588
307	18-30	female	Yes	Right lane	4	12.074	3650
308	18-30	female	Yes	Right lane	6	13.493	3652
309	18-30	female	Yes	Left lane	5	13.291	3680
310	18-30	female	Yes	Right lane	6	13.493	3698
311	18-30	female	Yes	Right lane	1	9.613	3780
312	18-30	female	Yes	Left lane	8	14.71	3793
313	18-30	female	Yes	Left lane	4	12.074	3795
314	18-30	female	Yes	Left lane	1	9.613	3865
315	18-30	female	Yes	Right lane	2	11.033	3869
316	18-30	female	Yes	Right lane	8	14.71	3871
317	18-30	female	Yes	Left lane	10	15.75	3874
318	18-30	female	Yes	Left lane	11	16.968	3879
319	18-30	female	Yes	Left lane	7	13.29	3902
320	18-30	female	Yes	Left lane	2	11.033	3914
321	18-30	female	Yes	Right lane	2	11.033	3928

322	18-30	female	Yes	Left lane	1	9.613	3959
323	18-30	female	Yes	Left lane	7	13.29	3995
324	18-30	female	Yes	Left lane	5	13.291	4000
325	18-30	female	Yes	Right lane	5	13.291	4001
326	18-30	female	Yes	Left lane	7	13.29	4003
327	18-30	female	Yes	Right lane	5	13.291	4015
328	18-30	female	Yes	Right lane	12	17.17	4024
329	18-30	female	Yes	Left lane	3	11.872	4036
330	18-30	female	Yes	Left lane	8	14.71	4057
331	18-30	female	Yes	Left lane	6	13.493	4061
332	18-30	female	Yes	Right lane	4	12.074	4082
333	18-30	female	Yes	Right lane	11	16.968	4113
334	18-30	female	Yes	Right lane	10	15.75	4126
335	18-30	female	Yes	Left lane	12	17.17	4229
336	18-30	female	Yes	Right lane	9	15.548	4248
337	18-30	female	Yes	Right lane	7	13.29	4313
338	18-30	female	Yes	Right lane	8	14.71	4313
339	18-30	female	Yes	Left lane	2	11.033	4339
340	18-30	female	Yes	Left lane	6	13.493	4350
341	18-30	female	Yes	Right lane	12	17.17	4358
342	18-30	female	Yes	Right lane	4	12.074	4394
343	18-30	female	Yes	Right lane	6	13.493	4416
344	18-30	female	Yes	Left lane	9	15.548	4417
345	18-30	female	Yes	Right lane	10	15.75	4428
346	18-30	female	Yes	Left lane	3	11.872	4452
347	18-30	female	Yes	Right lane	2	11.033	4462
348	18-30	female	Yes	Left lane	9	15.548	4492
349	18-30	female	Yes	Right lane	7	13.29	4500
350	18-30	female	Yes	Left lane	11	16.968	4514
351	18-30	female	Yes	Right lane	5	13.291	4547
352	18-30	female	Yes	Left lane	9	15.548	4552
353	18-30	female	Yes	Right lane	10	15.75	4584
354	18-30	female	Yes	Right lane	9	15.548	4650
355	18-30	female	Yes	Right lane	7	13.29	4704
356	18-30	female	Yes	Left lane	10	15.75	4705
357	18-30	female	Yes	Right lane	11	16.968	4720
358	18-30	female	Yes	Right lane	11	16.968	4731
359	18-30	female	Yes	Left lane	12	17.17	4753
360	18-30	female	Yes	Right lane	8	14.71	4758
361	18-30	female	Yes	Right lane	9	15.548	4800
362	18-30	female	Yes	Left lane	5	13.291	4806
363	18-30	female	Yes	Left lane	11	16.968	5126
364	18-30	female	Yes	Left lane	12	17.17	5168
365	18-30	female	Yes	Left lane	10	15.75	5192
366	18-30	male	No	Right lane	1	9.613	2294
367	18-30	male	No	Left lane	1	9.613	2865
368	18-30	male	No	Left lane	1	9.613	3049
369	18-30	male	No	Right lane	1	9.613	3326
370	18-30	male	No	Left lane	4	12.074	3368
371	18-30	male	No	Left lane	3	11.872	3464
372	18-30	male	No	Right lane	3	11.872	3532
373	18-30	male	No	Right lane	4	12.074	3560
374	18-30	male	No	Right lane	9	15.548	3588
375	18-30	male	No	Left lane	1	9.613	3661

376	18-30	male	No	Left lane	3	11.872	3665
377	18-30	male	No	Right lane	6	13.493	3679
378	18-30	male	No	Left lane	4	12.074	3693
379	18-30	male	No	Right lane	5	13.291	3720
380	18-30	male	No	Right lane	9	15.548	3726
381	18-30	male	No	Left lane	2	11.033	3728
382	18-30	male	No	Right lane	11	16.968	3736
383	18-30	male	No	Right lane	1	9.613	3753
384	18-30	male	No	Left lane	11	16.968	3754
385	18-30	male	No	Left lane	2	11.033	3758
386	18-30	male	No	Right lane	9	15.548	3771
387	18-30	male	No	Right lane	4	12.074	3773
388	18-30	male	No	Right lane	2	11.033	3780
389	18-30	male	No	Right lane	3	11.872	3780
390	18-30	male	No	Right lane	6	13.493	3828
391	18-30	male	No	Right lane	8	14.71	3833
392	18-30	male	No	Right lane	5	13.291	3839
393	18-30	male	No	Left lane	6	13.493	3873
394	18-30	male	No	Right lane	7	13.29	3882
395	18-30	male	No	Right lane	2	11.033	3887
396	18-30	male	No	Left lane	2	11.033	3914
397	18-30	male	No	Right lane	3	11.872	3926
398	18-30	male	No	Left lane	11	16.968	3938
399	18-30	male	No	Right lane	4	12.074	3964
400	18-30	male	No	Right lane	2	11.033	4003
401	18-30	male	No	Right lane	5	13.291	4008
402	18-30	male	No	Right lane	10	15.75	4018
403	18-30	male	No	Left lane	12	17.17	4120
404	18-30	male	No	Left lane	8	14.71	4133
405	18-30	male	No	Right lane	6	13.493	4216
406	18-30	male	No	Left lane	9	15.548	4217
407	18-30	male	No	Left lane	10	15.75	4259
408	18-30	male	No	Left lane	6	13.493	4269
409	18-30	male	No	Left lane	5	13.291	4292
410	18-30	male	No	Left lane	3	11.872	4297
411	18-30	male	No	Left lane	7	13.29	4314
412	18-30	male	No	Left lane	12	17.17	4331
413	18-30	male	No	Left lane	9	15.548	4359
414	18-30	male	No	Right lane	11	16.968	4361
415	18-30	male	No	Right lane	8	14.71	4392
416	18-30	male	No	Left lane	10	15.75	4402
417	18-30	male	No	Right lane	7	13.29	4435
418	18-30	male	No	Left lane	8	14.71	4440
419	18-30	male	No	Left lane	4	12.074	4449
420	18-30	male	No	Right lane	12	17.17	4514
421	18-30	male	No	Right lane	12	17.17	4529
422	18-30	male	No	Left lane	5	13.291	4535
423	18-30	male	No	Right lane	7	13.29	4561
424	18-30	male	No	Left lane	6	13.493	4592
425	18-30	male	No	Right lane	12	17.17	4598
426	18-30	male	No	Right lane	8	14.71	4610
427	18-30	male	No	Left lane	5	13.291	4682
428	18-30	male	No	Left lane	10	15.75	4683
429	18-30	male	No	Right lane	10	15.75	4708

430	18-30	male	No	Left lane	9	15.548	4713
431	18-30	male	No	Left lane	8	14.71	4757
432	18-30	male	No	Left lane	11	16.968	4765
433	18-30	male	No	Left lane	7	13.29	4766
434	18-30	male	No	Left lane	12	17.17	5029
435	18-30	male	No	Left lane	7	13.29	5048
436	18-30	male	No	Right lane	11	16.968	5068
437	18-30	male	No	Right lane	10	15.75	5125
438	18-30	female	No	Right lane	1	9.613	2464
439	18-30	female	No	Left lane	1	9.613	3260
440	18-30	female	No	Right lane	1	9.613	3347
441	18-30	female	No	Left lane	1	9.613	3397
442	18-30	female	No	Right lane	3	11.872	3416
443	18-30	female	No	Right lane	1	9.613	3434
444	18-30	female	No	Left lane	3	11.872	3504
445	18-30	female	No	Right lane	5	13.291	3518
446	18-30	female	No	Left lane	5	13.291	3539
447	18-30	female	No	Right lane	2	11.033	3580
448	18-30	female	No	Left lane	2	11.033	3653
449	18-30	female	No	Left lane	4	12.074	3672
450	18-30	female	No	Left lane	5	13.291	3680
451	18-30	female	No	Right lane	4	12.074	3680
452	18-30	female	No	Left lane	4	12.074	3682
453	18-30	female	No	Left lane	1	9.613	3736
454	18-30	female	No	Right lane	6	13.493	3781
455	18-30	female	No	Left lane	5	13.291	3800
456	18-30	female	No	Right lane	3	11.872	3814
457	18-30	female	No	Right lane	4	12.074	3879
458	18-30	female	No	Left lane	2	11.033	3950
459	18-30	female	No	Left lane	2	11.033	3952
460	18-30	female	No	Right lane	2	11.033	3999
461	18-30	female	No	Left lane	3	11.872	4002
462	18-30	female	No	Right lane	3	11.872	4013
463	18-30	female	No	Left lane	9	15.548	4014
464	18-30	female	No	Right lane	4	12.074	4030
465	18-30	female	No	Left lane	6	13.493	4047
466	18-30	female	No	Right lane	6	13.493	4060
467	18-30	female	No	Left lane	10	15.75	4079
468	18-30	female	No	Left lane	3	11.872	4088
469	18-30	female	No	Right lane	5	13.291	4148
470	18-30	female	No	Right lane	11	16.968	4173
471	18-30	female	No	Left lane	11	16.968	4192
472	18-30	female	No	Right lane	12	17.17	4216
473	18-30	female	No	Right lane	2	11.033	4240
474	18-30	female	No	Left lane	6	13.493	4251
475	18-30	female	No	Left lane	11	16.968	4258
476	18-30	female	No	Left lane	7	13.29	4258
477	18-30	female	No	Right lane	7	13.29	4285
478	18-30	female	No	Right lane	8	14.71	4288
479	18-30	female	No	Right lane	5	13.291	4296
480	18-30	female	No	Left lane	12	17.17	4319
481	18-30	female	No	Right lane	6	13.493	4324
482	18-30	female	No	Left lane	6	13.493	4369
483	18-30	female	No	Right lane	7	13.29	4369

484	18-30	female	No	Right lane	9	15.548	4371
485	18-30	female	No	Left lane	4	12.074	4386
486	18-30	female	No	Left lane	8	14.71	4386
487	18-30	female	No	Right lane	8	14.71	4403
488	18-30	female	No	Right lane	9	15.548	4403
489	18-30	female	No	Left lane	7	13.29	4419
490	18-30	female	No	Right lane	10	15.75	4426
491	18-30	female	No	Right lane	12	17.17	4429
492	18-30	female	No	Right lane	11	16.968	4449
493	18-30	female	No	Left lane	10	15.75	4458
494	18-30	female	No	Right lane	11	16.968	4492
495	18-30	female	No	Left lane	8	14.71	4547
496	18-30	female	No	Left lane	9	15.548	4548
497	18-30	female	No	Left lane	12	17.17	4579
498	18-30	female	No	Right lane	7	13.29	4602
499	18-30	female	No	Left lane	12	17.17	4648
500	18-30	female	No	Left lane	10	15.75	4663
501	18-30	female	No	Left lane	8	14.71	4692
502	18-30	female	No	Right lane	9	15.548	4782
503	18-30	female	No	Right lane	8	14.71	4831
504	18-30	female	No	Left lane	7	13.29	4853
505	18-30	female	No	Left lane	9	15.548	4881
506	18-30	female	No	Right lane	10	15.75	4920
507	18-30	female	No	Right lane	12	17.17	4985
508	40-50	male	Yes	Right lane	4	12.074	3519
509	40-50	male	Yes	Right lane	1	9.613	3602
510	40-50	male	Yes	Right lane	1	9.613	3684
511	40-50	male	Yes	Right lane	3	11.872	3704
512	40-50	male	Yes	Left lane	1	9.613	3717
513	40-50	male	Yes	Left lane	4	12.074	3891
514	40-50	male	Yes	Left lane	1	9.613	3931
515	40-50	male	Yes	Left lane	4	12.074	3972
516	40-50	male	Yes	Right lane	4	12.074	4014
517	40-50	male	Yes	Right lane	6	13.493	4037
518	40-50	male	Yes	Right lane	9	15.548	4049
519	40-50	male	Yes	Left lane	5	13.291	4050
520	40-50	male	Yes	Right lane	2	11.033	4098
521	40-50	male	Yes	Left lane	7	13.29	4214
522	40-50	male	Yes	Right lane	5	13.291	4250
523	40-50	male	Yes	Left lane	7	13.29	4255
524	40-50	male	Yes	Right lane	3	11.872	4289
525	40-50	male	Yes	Right lane	11	16.968	4347
526	40-50	male	Yes	Left lane	3	11.872	4388
527	40-50	male	Yes	Left lane	2	11.033	4401
528	40-50	male	Yes	Right lane	2	11.033	4438
529	40-50	male	Yes	Right lane	7	13.29	4453
530	40-50	male	Yes	Left lane	2	11.033	4461
531	40-50	male	Yes	Left lane	2	11.033	4471
532	40-50	male	Yes	Right lane	3	11.872	4475
533	40-50	male	Yes	Left lane	11	16.968	4529
534	40-50	male	Yes	Left lane	6	13.493	4536
535	40-50	male	Yes	Right lane	6	13.493	4558
536	40-50	male	Yes	Right lane	12	17.17	4591
537	40-50	male	Yes	Right lane	8	14.71	4595

538	40-50	male	Yes	Right lane	5	13.291	4606
539	40-50	male	Yes	Right lane	6	13.493	4616
540	40-50	male	Yes	Left lane	9	15.548	4647
541	40-50	male	Yes	Left lane	10	15.75	4649
542	40-50	male	Yes	Left lane	7	13.29	4650
543	40-50	male	Yes	Left lane	11	16.968	4652
544	40-50	male	Yes	Left lane	5	13.291	4684
545	40-50	male	Yes	Left lane	5	13.291	4712
546	40-50	male	Yes	Left lane	12	17.17	4758
547	40-50	male	Yes	Right lane	7	13.29	4762
548	40-50	male	Yes	Left lane	6	13.493	4778
549	40-50	male	Yes	Left lane	10	15.75	4810
550	40-50	male	Yes	Right lane	4	12.074	4813
551	40-50	male	Yes	Left lane	8	14.71	4816
552	40-50	male	Yes	Left lane	9	15.548	4824
553	40-50	male	Yes	Right lane	11	16.968	4854
554	40-50	male	Yes	Left lane	8	14.71	4888
555	40-50	male	Yes	Left lane	8	14.71	4900
556	40-50	male	Yes	Right lane	1	9.613	5012
557	40-50	male	Yes	Right lane	9	15.548	5072
558	40-50	male	Yes	Right lane	10	15.75	5102
559	40-50	male	Yes	Left lane	10	15.75	5113
560	40-50	male	Yes	Right lane	10	15.75	5113
561	40-50	male	Yes	Right lane	11	16.968	5146
562	40-50	male	Yes	Right lane	8	14.71	5149
563	40-50	male	Yes	Right lane	12	17.17	5194
564	40-50	male	Yes	Right lane	12	17.17	5236
565	40-50	male	Yes	Left lane	9	15.548	5314
566	30-40	female	Yes	Right lane	1	9.613	3126
567	30-40	female	Yes	Left lane	3	11.872	3351
568	30-40	female	Yes	Right lane	3	11.872	3391
569	30-40	female	Yes	Left lane	1	9.613	3396
570	30-40	female	Yes	Left lane	3	11.872	3501
571	30-40	female	Yes	Right lane	1	9.613	3548
572	30-40	female	Yes	Left lane	5	13.291	3843
573	30-40	female	Yes	Right lane	1	9.613	3854
574	30-40	female	Yes	Right lane	3	11.872	3904
575	30-40	female	Yes	Left lane	4	12.074	3951
576	30-40	female	Yes	Left lane	7	13.29	3969
577	30-40	female	Yes	Left lane	1	9.613	4025
578	30-40	female	Yes	Right lane	5	13.291	4054
579	30-40	female	Yes	Right lane	2	11.033	4064
580	30-40	female	Yes	Right lane	6	13.493	4136
581	30-40	female	Yes	Left lane	4	12.074	4144
582	30-40	female	Yes	Right lane	4	12.074	4146
583	30-40	female	Yes	Left lane	2	11.033	4158
584	30-40	female	Yes	Right lane	11	16.968	4166
585	30-40	female	Yes	Left lane	4	12.074	4167
586	30-40	female	Yes	Right lane	9	15.548	4186
587	30-40	female	Yes	Right lane	2	11.033	4204
588	30-40	female	Yes	Right lane	2	11.033	4232
589	30-40	female	Yes	Right lane	7	13.29	4247
590	30-40	female	Yes	Right lane	3	11.872	4299
591	30-40	female	Yes	Left lane	8	14.71	4316

592	30-40	female	Yes	Right lane	6	13.493	4348
593	30-40	female	Yes	Right lane	7	13.29	4351
594	30-40	female	Yes	Right lane	5	13.291	4353
595	30-40	female	Yes	Left lane	5	13.291	4394
596	30-40	female	Yes	Right lane	5	13.291	4395
597	30-40	female	Yes	Left lane	1	9.613	4427
598	30-40	female	Yes	Left lane	6	13.493	4450
599	30-40	female	Yes	Left lane	9	15.548	4459
600	30-40	female	Yes	Right lane	4	12.074	4473
601	30-40	female	Yes	Left lane	11	16.968	4495
602	30-40	female	Yes	Left lane	3	11.872	4513
603	30-40	female	Yes	Right lane	4	12.074	4514
604	30-40	female	Yes	Right lane	12	17.17	4528
605	30-40	female	Yes	Left lane	6	13.493	4562
606	30-40	female	Yes	Left lane	12	17.17	4604
607	30-40	female	Yes	Left lane	12	17.17	4658
608	30-40	female	Yes	Left lane	7	13.29	4667
609	30-40	female	Yes	Left lane	10	15.75	4672
610	30-40	female	Yes	Left lane	10	15.75	4682
611	30-40	female	Yes	Left lane	6	13.493	4731
612	30-40	female	Yes	Right lane	8	14.71	4735
613	30-40	female	Yes	Right lane	10	15.75	4739
614	30-40	female	Yes	Right lane	11	16.968	4752
615	30-40	female	Yes	Left lane	2	11.033	4766
616	30-40	female	Yes	Left lane	8	14.71	4777
617	30-40	female	Yes	Left lane	9	15.548	4781
618	30-40	female	Yes	Right lane	8	14.71	4800
619	30-40	female	Yes	Left lane	5	13.291	4856
620	30-40	female	Yes	Left lane	10	15.75	4870
621	30-40	female	Yes	Left lane	11	16.968	4898
622	30-40	female	Yes	Left lane	2	11.033	4919
623	30-40	female	Yes	Right lane	10	15.75	4981
624	30-40	female	Yes	Right lane	6	13.493	5027
625	30-40	female	Yes	Left lane	12	17.17	5034
626	30-40	female	Yes	Right lane	11	16.968	5040
627	30-40	female	Yes	Left lane	7	13.29	5120
628	30-40	female	Yes	Left lane	11	16.968	5125
629	30-40	female	Yes	Right lane	7	13.29	5136
630	30-40	female	Yes	Right lane	12	17.17	5138
631	30-40	female	Yes	Left lane	9	15.548	5145
632	30-40	female	Yes	Right lane	9	15.548	5176
633	30-40	female	Yes	Right lane	8	14.71	5181
634	30-40	female	Yes	Right lane	12	17.17	5185
635	30-40	female	Yes	Right lane	10	15.75	5192
636	30-40	female	Yes	Right lane	9	15.548	5248
637	50-60	male	Yes	Right lane	1	9.613	2547
638	50-60	male	Yes	Left lane	1	9.613	3258
639	50-60	male	Yes	Left lane	3	11.872	3413
640	50-60	male	Yes	Left lane	1	9.613	3449
641	50-60	male	Yes	Right lane	3	11.872	3495
642	50-60	male	Yes	Right lane	1	9.613	3517
643	50-60	male	Yes	Left lane	3	11.872	3648
644	50-60	male	Yes	Right lane	4	12.074	3880
645	50-60	male	Yes	Left lane	4	12.074	4003

646	50-60	male	Yes	Left lane	4	12.074	4012
647	50-60	male	Yes	Right lane	2	11.033	4173
648	50-60	male	Yes	Left lane	1	9.613	4183
649	50-60	male	Yes	Left lane	3	11.872	4191
650	50-60	male	Yes	Right lane	2	11.033	4207
651	50-60	male	Yes	Left lane	6	13.493	4216
652	50-60	male	Yes	Right lane	5	13.291	4246
653	50-60	male	Yes	Left lane	5	13.291	4255
654	50-60	male	Yes	Right lane	3	11.872	4258
655	50-60	male	Yes	Right lane	6	13.493	4303
656	50-60	male	Yes	Left lane	7	13.29	4377
657	50-60	male	Yes	Left lane	10	15.75	4414
658	50-60	male	Yes	Left lane	2	11.033	4414
659	50-60	male	Yes	Left lane	2	11.033	4420
660	50-60	male	Yes	Left lane	4	12.074	4450
661	50-60	male	Yes	Right lane	5	13.291	4489
662	50-60	male	Yes	Right lane	4	12.074	4491
663	50-60	male	Yes	Right lane	2	11.033	4498
664	50-60	male	Yes	Left lane	12	17.17	4507
665	50-60	male	Yes	Right lane	10	15.75	4547
666	50-60	male	Yes	Left lane	11	16.968	4562
667	50-60	male	Yes	Right lane	9	15.548	4564
668	50-60	male	Yes	Left lane	5	13.291	4570
669	50-60	male	Yes	Right lane	12	17.17	4578
670	50-60	male	Yes	Left lane	8	14.71	4586
671	50-60	male	Yes	Right lane	3	11.872	4607
672	50-60	male	Yes	Right lane	1	9.613	4617
673	50-60	male	Yes	Right lane	5	13.291	4626
674	50-60	male	Yes	Right lane	9	15.548	4652
675	50-60	male	Yes	Left lane	6	13.493	4684
676	50-60	male	Yes	Right lane	7	13.29	4693
677	50-60	male	Yes	Right lane	8	14.71	4719
678	50-60	male	Yes	Left lane	8	14.71	4735
679	50-60	male	Yes	Left lane	10	15.75	4735
680	50-60	male	Yes	Left lane	10	15.75	4750
681	50-60	male	Yes	Left lane	2	11.033	4765
682	50-60	male	Yes	Right lane	4	12.074	4783
683	50-60	male	Yes	Right lane	6	13.493	4796
684	50-60	male	Yes	Left lane	7	13.29	4800
685	50-60	male	Yes	Left lane	7	13.29	4800
686	50-60	male	Yes	Right lane	9	15.548	4821
687	50-60	male	Yes	Left lane	11	16.968	4828
688	50-60	male	Yes	Right lane	7	13.29	4836
689	50-60	male	Yes	Left lane	5	13.291	4889
690	50-60	male	Yes	Right lane	11	16.968	4893
691	50-60	male	Yes	Right lane	12	17.17	4914
692	50-60	male	Yes	Left lane	11	16.968	4950
693	50-60	male	Yes	Right lane	8	14.71	4962
694	50-60	male	Yes	Right lane	8	14.71	5021
695	50-60	male	Yes	Right lane	6	13.493	5029
696	50-60	male	Yes	Right lane	10	15.75	5062
697	50-60	male	Yes	Left lane	12	17.17	5077
698	50-60	male	Yes	Left lane	6	13.493	5099
699	50-60	male	Yes	Right lane	11	16.968	5104

700	50-60	male	Yes	Left lane	9	15.548	5136
701	50-60	male	Yes	Right lane	11	16.968	5150
702	50-60	male	Yes	Left lane	9	15.548	5230
703	50-60	male	Yes	Right lane	7	13.29	5249
704	50-60	male	Yes	Right lane	10	15.75	5261
705	50-60	male	Yes	Right lane	12	17.17	5318
706	50-60	male	Yes	Left lane	8	14.71	5318
707	50-60	male	Yes	Left lane	9	15.548	5328
708	30-40	female	Yes	Left lane	1	9.613	3989
709	30-40	female	Yes	Left lane	3	11.872	4317
710	30-40	female	Yes	Left lane	4	12.074	4459
711	30-40	female	Yes	Right lane	5	13.291	4638
712	30-40	female	Yes	Right lane	8	14.71	4712
713	30-40	female	Yes	Left lane	11	16.968	4746
714	30-40	female	Yes	Right lane	2	11.033	4747
715	30-40	female	Yes	Right lane	9	15.548	4810
716	30-40	female	Yes	Right lane	7	13.29	4881
717	30-40	female	Yes	Left lane	6	13.493	4907
718	30-40	female	Yes	Left lane	12	17.17	5066
719	30-40	female	Yes	Right lane	10	15.75	5271
720	18-30	female	No	Left lane	7	13.29	2739
721	18-30	female	No	Left lane	4	12.074	3141
722	18-30	female	No	Left lane	10	15.75	3282
723	18-30	female	No	Right lane	6	13.493	3339
724	18-30	female	No	Right lane	6	13.493	3448
725	18-30	female	No	Right lane	3	11.872	3546
726	18-30	female	No	Left lane	1	9.613	3665
727	18-30	female	No	Left lane	3	11.872	3759
728	18-30	female	No	Right lane	3	11.872	3773
729	18-30	female	No	Right lane	4	12.074	3780
730	18-30	female	No	Left lane	2	11.033	3814
731	18-30	female	No	Right lane	11	16.968	3839
732	18-30	female	No	Right lane	1	9.613	3859
733	18-30	female	No	Left lane	2	11.033	3869
734	18-30	female	No	Right lane	11	16.968	3872
735	18-30	female	No	Left lane	6	13.493	3919
736	18-30	female	No	Right lane	9	15.548	3974
737	18-30	female	No	Left lane	12	17.17	3981
738	18-30	female	No	Left lane	11	16.968	3992
739	18-30	female	No	Left lane	4	12.074	4099
740	18-30	female	No	Left lane	8	14.71	4099
741	18-30	female	No	Left lane	6	13.493	4121
742	18-30	female	No	Right lane	9	15.548	4181
743	18-30	female	No	Right lane	12	17.17	4186
744	18-30	female	No	Right lane	4	12.074	4193
745	18-30	female	No	Right lane	5	13.291	4195
746	18-30	female	No	Left lane	9	15.548	4200
747	18-30	female	No	Left lane	12	17.17	4233
748	18-30	female	No	Right lane	12	17.17	4246
749	18-30	female	No	Left lane	3	11.872	4250
750	18-30	female	No	Left lane	8	14.71	4271
751	18-30	female	No	Left lane	7	13.29	4292
752	18-30	female	No	Right lane	2	11.033	4296
753	18-30	female	No	Right lane	4	12.074	4313

754	18-30	female	No	Right lane	10	15.75	4328
755	18-30	female	No	Right lane	5	13.291	4336
756	18-30	female	No	Right lane	6	13.493	4349
757	18-30	female	No	Left lane	7	13.29	4358
758	18-30	female	No	Left lane	9	15.548	4359
759	18-30	female	No	Right lane	7	13.29	4372
760	18-30	female	No	Right lane	3	11.872	4382
761	18-30	female	No	Right lane	7	13.29	4386
762	18-30	female	No	Right lane	8	14.71	4396
763	18-30	female	No	Right lane	12	17.17	4397
764	18-30	female	No	Right lane	8	14.71	4417
765	18-30	female	No	Left lane	8	14.71	4419
766	18-30	female	No	Left lane	11	16.968	4433
767	18-30	female	No	Left lane	10	15.75	4455
768	18-30	female	No	Right lane	10	15.75	4499
769	18-30	female	No	Left lane	12	17.17	4503
770	18-30	female	No	Left lane	10	15.75	4504
771	18-30	female	No	Right lane	9	15.548	4510
772	18-30	female	No	Right lane	5	13.291	4524
773	18-30	female	No	Right lane	10	15.75	4529
774	18-30	female	No	Left lane	9	15.548	4540
775	18-30	female	No	Left lane	5	13.291	4577
776	18-30	female	No	Right lane	1	9.613	4591
777	18-30	female	No	Left lane	4	12.074	4593
778	18-30	female	No	Left lane	5	13.291	4599
779	18-30	female	No	Right lane	11	16.968	4610
780	18-30	female	No	Left lane	1	9.613	4632
781	18-30	female	No	Right lane	1	9.613	4638
782	18-30	female	No	Left lane	5	13.291	4650
783	18-30	female	No	Left lane	2	11.033	4727
784	18-30	female	No	Left lane	1	9.613	4794
785	18-30	female	No	Right lane	7	13.29	4846
786	18-30	female	No	Left lane	6	13.493	4884
787	18-30	female	No	Right lane	2	11.033	4884
788	18-30	female	No	Left lane	11	16.968	4952
789	18-30	female	No	Left lane	3	11.872	5058
790	18-30	female	No	Right lane	8	14.71	5182
791	18-30	female	No	Right lane	2	11.033	5230
792	30-40	male	No	Right lane	1	9.613	2740
793	30-40	male	No	Right lane	4	12.074	3565
794	30-40	male	No	Right lane	3	11.872	3684
795	30-40	male	No	Right lane	6	13.493	3749
796	30-40	male	No	Left lane	5	13.291	3894
797	30-40	male	No	Left lane	2	11.033	4371
798	30-40	male	No	Left lane	7	13.29	4663
799	30-40	male	No	Left lane	8	14.71	4712
800	30-40	male	No	Left lane	9	15.548	4812
801	30-40	male	No	Right lane	12	17.17	4882
802	30-40	male	No	Right lane	11	16.968	4979
803	30-40	male	No	Left lane	10	15.75	5094
804	30-40	male	Yes	Left lane	4	12.074	3586
805	30-40	male	Yes	Left lane	1	9.613	3762
806	30-40	male	Yes	Left lane	3	11.872	3861
807	30-40	male	Yes	Right lane	9	15.548	3916

808	30-40	male	Yes	Left lane	12	17.17	3983
809	30-40	male	Yes	Right lane	5	13.291	4298
810	30-40	male	Yes	Right lane	2	11.033	4313
811	30-40	male	Yes	Left lane	6	13.493	4670
812	30-40	male	Yes	Right lane	7	13.29	4786
813	30-40	male	Yes	Left lane	11	16.968	4795
814	30-40	male	Yes	Right lane	8	14.71	4863
815	30-40	male	Yes	Right lane	10	15.75	5001
816	40-50	male	Yes	Right lane	1	9.613	3447
817	40-50	male	Yes	Right lane	3	11.872	4185
818	40-50	male	Yes	Left lane	5	13.291	4259
819	40-50	male	Yes	Left lane	10	15.75	4506
820	40-50	male	Yes	Left lane	8	14.71	4547
821	40-50	male	Yes	Left lane	2	11.033	4598
822	40-50	male	Yes	Left lane	7	13.29	4673
823	40-50	male	Yes	Left lane	9	15.548	4705
824	40-50	male	Yes	Right lane	6	13.493	4743
825	40-50	male	Yes	Right lane	12	17.17	4819
826	40-50	male	Yes	Right lane	11	16.968	5019
827	18-30	female	Yes	Left lane	3	11.872	3824
828	18-30	female	Yes	Left lane	4	12.074	3891
829	18-30	female	Yes	Left lane	1	9.613	4306
830	18-30	female	Yes	Left lane	11	16.968	4396
831	18-30	female	Yes	Right lane	2	11.033	4515
832	18-30	female	Yes	Right lane	9	15.548	4573
833	18-30	female	Yes	Right lane	8	14.71	4624
834	18-30	female	Yes	Left lane	12	17.17	4682
835	18-30	female	Yes	Right lane	7	13.29	4772
836	18-30	female	Yes	Right lane	5	13.291	4825
837	18-30	female	Yes	Right lane	10	15.75	4925
838	30-40	female	Yes	Right lane	3	11.872	3819
839	30-40	female	Yes	Right lane	1	9.613	3980
840	30-40	female	Yes	Left lane	6	13.493	4261
841	30-40	female	Yes	Left lane	7	13.29	4400
842	30-40	female	Yes	Left lane	2	11.033	4401
843	30-40	female	Yes	Right lane	4	12.074	4422
844	30-40	female	Yes	Right lane	6	13.493	4704
845	30-40	female	Yes	Left lane	10	15.75	5014
846	30-40	female	Yes	Left lane	5	13.291	5026
847	30-40	female	Yes	Left lane	8	14.71	5081
848	30-40	female	Yes	Left lane	9	15.548	5147
849	30-40	female	Yes	Right lane	11	16.968	5160
850	30-40	female	Yes	Right lane	12	17.17	5202
851	40-50	male	Yes	Right lane	1	9.613	3365
852	40-50	male	Yes	Right lane	3	11.872	3658
853	40-50	male	Yes	Left lane	2	11.033	4147
854	40-50	male	Yes	Right lane	6	13.493	4185
855	40-50	male	Yes	Right lane	4	12.074	4253
856	40-50	male	Yes	Left lane	5	13.291	4261
857	40-50	male	Yes	Left lane	8	14.71	4744
858	40-50	male	Yes	Left lane	9	15.548	4855
859	40-50	male	Yes	Left lane	7	13.29	4867
860	40-50	male	Yes	Right lane	11	16.968	4895
861	40-50	male	Yes	Right lane	12	17.17	4927

862	40-50	male	Yes	Left lane	10	15.75	5016
863	50-60	male	Yes	Left lane	1	9.613	3681
864	50-60	male	Yes	Right lane	5	13.291	4113
865	50-60	male	Yes	Right lane	2	11.033	4166
866	50-60	male	Yes	Left lane	4	12.074	4266
867	50-60	male	Yes	Left lane	3	11.872	4300
868	50-60	male	Yes	Left lane	6	13.493	4324
869	50-60	male	Yes	Left lane	11	16.968	4709
870	50-60	male	Yes	Right lane	7	13.29	4783
871	50-60	male	Yes	Right lane	8	14.71	4813
872	50-60	male	Yes	Left lane	12	17.17	4836
873	50-60	male	Yes	Right lane	10	15.75	4992
874	50-60	male	Yes	Right lane	9	15.548	5087
875	30-40	male	Yes	Left lane	1	9.613	3715
876	30-40	male	Yes	Left lane	3	11.872	3772
877	30-40	male	Yes	Right lane	2	11.033	4124
878	30-40	male	Yes	Left lane	6	13.493	4166
879	30-40	male	Yes	Right lane	7	13.29	4316
880	30-40	male	Yes	Left lane	4	12.074	4684
881	30-40	male	Yes	Right lane	5	13.291	4868
882	30-40	male	Yes	Right lane	9	15.548	4887
883	30-40	male	Yes	Left lane	12	17.17	4932
884	30-40	male	Yes	Right lane	8	14.71	5054
885	30-40	male	Yes	Right lane	10	15.75	5098
886	30-40	male	Yes	Left lane	11	16.968	5153
887	50-60	male	Yes	Right lane	3	11.872	4271
888	50-60	male	Yes	Left lane	2	11.033	4396
889	50-60	male	Yes	Right lane	6	13.493	4493
890	50-60	male	Yes	Right lane	1	9.613	4552
891	50-60	male	Yes	Left lane	5	13.291	4591
892	50-60	male	Yes	Left lane	7	13.29	4621
893	50-60	male	Yes	Right lane	11	16.968	4820
894	50-60	male	Yes	Left lane	8	14.71	4826
895	50-60	male	Yes	Right lane	4	12.074	4834
896	50-60	male	Yes	Left lane	9	15.548	5002
897	50-60	male	Yes	Right lane	12	17.17	5258
898	50-60	female	Yes	Left lane	3	11.872	3592
899	50-60	female	Yes	Left lane	1	9.613	3637
900	50-60	female	Yes	Right lane	2	11.033	4047
901	50-60	female	Yes	Left lane	4	12.074	4515
902	50-60	female	Yes	Right lane	5	13.291	4592
903	50-60	female	Yes	Right lane	8	14.71	4600
904	50-60	female	Yes	Left lane	11	16.968	4788
905	50-60	female	Yes	Right lane	9	15.548	4883
906	50-60	female	Yes	Left lane	12	17.17	5066
907	50-60	female	Yes	Right lane	7	13.29	5073
908	50-60	female	Yes	Right lane	10	15.75	5296
909	18-30	female	Yes	Left lane	1	9.613	2913
910	18-30	female	Yes	Left lane	8	14.71	3391
911	18-30	female	Yes	Right lane	1	9.613	3404
912	18-30	female	Yes	Left lane	10	15.75	3545
913	18-30	female	Yes	Left lane	3	11.872	3547
914	18-30	female	Yes	Left lane	4	12.074	3580
915	18-30	female	Yes	Left lane	10	15.75	3582

916	18-30	female	Yes	Right lane	4	12.074	3592
917	18-30	female	Yes	Right lane	11	16.968	3626
918	18-30	female	Yes	Left lane	5	13.291	3646
919	18-30	female	Yes	Right lane	2	11.033	3696
920	18-30	female	Yes	Left lane	4	12.074	3697
921	18-30	female	Yes	Right lane	1	9.613	3699
922	18-30	female	Yes	Left lane	8	14.71	3716
923	18-30	female	Yes	Left lane	5	13.291	3725
924	18-30	female	Yes	Right lane	12	17.17	3739
925	18-30	female	Yes	Right lane	12	17.17	3750
926	18-30	female	Yes	Left lane	2	11.033	3779
927	18-30	female	Yes	Left lane	3	11.872	3817
928	18-30	female	Yes	Right lane	5	13.291	3831
929	18-30	female	Yes	Right lane	11	16.968	3843
930	18-30	female	Yes	Right lane	4	12.074	3845
931	18-30	female	Yes	Right lane	9	15.548	3851
932	18-30	female	Yes	Right lane	3	11.872	3862
933	18-30	female	Yes	Left lane	6	13.493	3871
934	18-30	female	Yes	Left lane	10	15.75	3879
935	18-30	female	Yes	Left lane	7	13.29	3884
936	18-30	female	Yes	Left lane	12	17.17	3894
937	18-30	female	Yes	Right lane	6	13.493	3895
938	18-30	female	Yes	Right lane	3	11.872	3924
939	18-30	female	Yes	Right lane	6	13.493	3935
940	18-30	female	Yes	Left lane	2	11.033	3940
941	18-30	female	Yes	Right lane	3	11.872	3954
942	18-30	female	Yes	Right lane	11	16.968	3964
943	18-30	female	Yes	Left lane	11	16.968	3973
944	18-30	female	Yes	Right lane	2	11.033	3983
945	18-30	female	Yes	Right lane	5	13.291	3984
946	18-30	female	Yes	Left lane	1	9.613	3987
947	18-30	female	Yes	Right lane	10	15.75	4005
948	18-30	female	Yes	Left lane	6	13.493	4012
949	18-30	female	Yes	Left lane	2	11.033	4014
950	18-30	female	Yes	Left lane	9	15.548	4017
951	18-30	female	Yes	Right lane	8	14.71	4034
952	18-30	female	Yes	Left lane	1	9.613	4051
953	18-30	female	Yes	Right lane	8	14.71	4059
954	18-30	female	Yes	Right lane	9	15.548	4063
955	18-30	female	Yes	Right lane	1	9.613	4072
956	18-30	female	Yes	Left lane	4	12.074	4079
957	18-30	female	Yes	Left lane	12	17.17	4079
958	18-30	female	Yes	Right lane	5	13.291	4105
959	18-30	female	Yes	Left lane	7	13.29	4125
960	18-30	female	Yes	Right lane	12	17.17	4135
961	18-30	female	Yes	Right lane	7	13.29	4138
962	18-30	female	Yes	Left lane	7	13.29	4146
963	18-30	female	Yes	Left lane	9	15.548	4161
964	18-30	female	Yes	Left lane	6	13.493	4182
965	18-30	female	Yes	Right lane	4	12.074	4182
966	18-30	female	Yes	Left lane	3	11.872	4184
967	18-30	female	Yes	Left lane	9	15.548	4186
968	18-30	female	Yes	Left lane	5	13.291	4217
969	18-30	female	Yes	Right lane	8	14.71	4225

970	18-30	female	Yes	Left lane	8	14.71	4227
971	18-30	female	Yes	Right lane	7	13.29	4231
972	18-30	female	Yes	Left lane	11	16.968	4246
973	18-30	female	Yes	Right lane	10	15.75	4248
974	18-30	female	Yes	Right lane	10	15.75	4306
975	18-30	female	Yes	Right lane	7	13.29	4336
976	18-30	female	Yes	Right lane	2	11.033	4394
977	18-30	female	Yes	Right lane	6	13.493	4434
978	18-30	female	Yes	Right lane	9	15.548	4517
979	18-30	female	Yes	Left lane	12	17.17	4517
980	18-30	female	Yes	Left lane	11	16.968	4551
981	40-50	male	Yes	Right lane	1	9.613	3864
982	40-50	male	Yes	Right lane	4	12.074	3906
983	40-50	male	Yes	Right lane	3	11.872	4091
984	40-50	male	Yes	Left lane	5	13.291	4102
985	40-50	male	Yes	Left lane	6	13.493	4288
986	40-50	male	Yes	Left lane	2	11.033	4419
987	40-50	male	Yes	Left lane	7	13.29	4477
988	40-50	male	Yes	Right lane	6	13.493	4479
989	40-50	male	Yes	Left lane	10	15.75	4525
990	40-50	male	Yes	Right lane	11	16.968	4568
991	40-50	male	Yes	Right lane	12	17.17	4776
992	40-50	male	Yes	Left lane	8	14.71	5055
993	40-50	male	Yes	Left lane	9	15.548	5198
994	30-40	male	Yes	Right lane	3	11.872	3181
995	30-40	male	Yes	Right lane	4	12.074	3516
996	30-40	male	Yes	Right lane	1	9.613	3967
997	30-40	male	Yes	Left lane	5	13.291	4039
998	30-40	male	Yes	Left lane	2	11.033	4235
999	30-40	male	Yes	Left lane	7	13.29	4584
1000	30-40	male	Yes	Left lane	10	15.75	4802
1001	30-40	male	Yes	Left lane	6	13.493	4834
1002	30-40	male	Yes	Right lane	6	13.493	4895
1003	30-40	male	Yes	Left lane	8	14.71	4940
1004	30-40	male	Yes	Left lane	9	15.548	5046
1005	30-40	male	Yes	Right lane	12	17.17	5264
1006	50-60	male	Yes	Left lane	3	11.872	3116
1007	50-60	male	Yes	Left lane	1	9.613	3538
1008	50-60	male	Yes	Left lane	4	12.074	4193
1009	50-60	male	Yes	Right lane	2	11.033	4197
1010	50-60	male	Yes	Right lane	5	13.291	4352
1011	50-60	male	Yes	Right lane	7	13.29	4496
1012	50-60	male	Yes	Right lane	8	14.71	4558
1013	50-60	male	Yes	Right lane	10	15.75	4670
1014	50-60	male	Yes	Left lane	11	16.968	4847
1015	50-60	male	Yes	Left lane	12	17.17	4902
1016	50-60	male	Yes	Right lane	9	15.548	4947
1017	40-50	male	Yes	Right lane	3	11.872	3773
1018	40-50	male	Yes	Right lane	1	9.613	4214
1019	40-50	male	Yes	Left lane	2	11.033	4258
1020	40-50	male	Yes	Left lane	10	15.75	4354
1021	40-50	male	Yes	Left lane	5	13.291	4538
1022	40-50	male	Yes	Right lane	4	12.074	4613
1023	40-50	male	Yes	Right lane	12	17.17	4714

1024	40-50	male	Yes	Right lane	6	13.493	4830
1025	40-50	male	Yes	Left lane	7	13.29	4938
1026	40-50	male	Yes	Left lane	9	15.548	5043
1027	40-50	male	Yes	Left lane	8	14.71	5100
1028	18-30	female	No	Left lane	1	9.613	3273
1029	18-30	female	No	Left lane	12	17.17	4258
1030	18-30	female	No	Left lane	6	13.493	4305
1031	18-30	female	No	Right lane	2	11.033	4389
1032	18-30	female	No	Left lane	3	11.872	4524
1033	18-30	female	No	Left lane	4	12.074	4543
1034	18-30	female	No	Right lane	5	13.291	4654
1035	18-30	female	No	Right lane	7	13.29	4691
1036	18-30	female	No	Right lane	9	15.548	4798
1037	18-30	female	No	Right lane	8	14.71	5214
1038	18-30	female	No	Right lane	10	15.75	5284
1039	40-50	male	Yes	Left lane	1	9.613	3494
1040	40-50	male	Yes	Left lane	3	11.872	3514
1041	40-50	male	Yes	Left lane	4	12.074	4339
1042	40-50	male	Yes	Right lane	5	13.291	4480
1043	40-50	male	Yes	Right lane	2	11.033	4496
1044	40-50	male	Yes	Right lane	9	15.548	4899
1045	40-50	male	Yes	Left lane	6	13.493	5043
1046	40-50	male	Yes	Right lane	7	13.29	5052
1047	40-50	male	Yes	Right lane	8	14.71	5255
1048	40-50	male	Yes	Left lane	12	17.17	5261
1049	40-50	male	Yes	Right lane	10	15.75	5312
1050	18-30	male	No	Left lane	4	12.074	3401
1051	18-30	male	No	Left lane	3	11.872	3430
1052	18-30	male	No	Left lane	1	9.613	3502
1053	18-30	male	No	Right lane	5	13.291	3858
1054	18-30	male	No	Right lane	2	11.033	4180
1055	18-30	male	No	Left lane	6	13.493	4662
1056	18-30	male	No	Right lane	8	14.71	4883
1057	18-30	male	No	Right lane	10	15.75	4883
1058	18-30	male	No	Left lane	11	16.968	5046
1059	18-30	male	No	Right lane	7	13.29	5053
1060	30-40	female	Yes	Left lane	3	11.872	2425
1061	30-40	female	Yes	Left lane	1	9.613	3232
1062	30-40	female	Yes	Left lane	4	12.074	3613
1063	30-40	female	Yes	Right lane	2	11.033	4196
1064	30-40	female	Yes	Right lane	5	13.291	4333
1065	30-40	female	Yes	Left lane	6	13.493	4351
1066	30-40	female	Yes	Left lane	11	16.968	4591
1067	30-40	female	Yes	Right lane	10	15.75	4914
1068	30-40	female	Yes	Right lane	7	13.29	4929
1069	30-40	female	Yes	Right lane	8	14.71	5005
1070	30-40	female	Yes	Left lane	12	17.17	5103
1071	30-40	female	Yes	Right lane	9	15.548	5117
1072	30-40	male	Yes	Left lane	1	9.613	3213
1073	30-40	male	Yes	Left lane	3	11.872	3698
1074	30-40	male	Yes	Right lane	5	13.291	4280
1075	30-40	male	Yes	Right lane	8	14.71	4577
1076	30-40	male	Yes	Right lane	2	11.033	4666
1077	30-40	male	Yes	Left lane	4	12.074	4673

1078	30-40	male	Yes	Right lane	9	15.548	4746
1079	30-40	male	Yes	Left lane	11	16.968	4815
1080	30-40	male	Yes	Right lane	10	15.75	4892
1081	30-40	male	Yes	Right lane	7	13.29	5091
1082	30-40	male	Yes	Left lane	12	17.17	5167
1083	18-30	male	Yes	Right lane	4	12.074	2724
1084	18-30	male	Yes	Right lane	3	11.872	2832
1085	18-30	male	Yes	Left lane	10	15.75	3181
1086	18-30	male	Yes	Left lane	7	13.29	3282
1087	18-30	male	Yes	Left lane	9	15.548	3304
1088	18-30	male	Yes	Left lane	9	15.548	3331
1089	18-30	male	Yes	Left lane	8	14.71	3385
1090	18-30	male	Yes	Left lane	8	14.71	3529
1091	18-30	male	Yes	Left lane	5	13.291	3552
1092	18-30	male	Yes	Right lane	6	13.493	3566
1093	18-30	male	Yes	Right lane	1	9.613	3597
1094	18-30	male	Yes	Right lane	1	9.613	3606
1095	18-30	male	Yes	Left lane	1	9.613	3640
1096	18-30	male	Yes	Right lane	3	11.872	3649
1097	18-30	male	Yes	Right lane	5	13.291	3692
1098	18-30	male	Yes	Right lane	11	16.968	3715
1099	18-30	male	Yes	Left lane	2	11.033	3746
1100	18-30	male	Yes	Left lane	2	11.033	3777
1101	18-30	male	Yes	Right lane	6	13.493	3847
1102	18-30	male	Yes	Right lane	3	11.872	3859
1103	18-30	male	Yes	Left lane	12	17.17	3884
1104	18-30	male	Yes	Right lane	10	15.75	3888
1105	18-30	male	Yes	Right lane	4	12.074	3924
1106	18-30	male	Yes	Right lane	12	17.17	3933
1107	18-30	male	Yes	Right lane	9	15.548	3948
1108	18-30	male	Yes	Left lane	11	16.968	3962
1109	18-30	male	Yes	Left lane	3	11.872	3966
1110	18-30	male	Yes	Right lane	10	15.75	3973
1111	18-30	male	Yes	Left lane	4	12.074	3986
1112	18-30	male	Yes	Left lane	6	13.493	3995
1113	18-30	male	Yes	Left lane	10	15.75	3999
1114	18-30	male	Yes	Right lane	1	9.613	4024
1115	18-30	male	Yes	Right lane	5	13.291	4026
1116	18-30	male	Yes	Left lane	12	17.17	4044
1117	18-30	male	Yes	Left lane	4	12.074	4053
1118	18-30	male	Yes	Left lane	5	13.291	4063
1119	18-30	male	Yes	Left lane	2	11.033	4081
1120	18-30	male	Yes	Right lane	7	13.29	4081
1121	18-30	male	Yes	Right lane	11	16.968	4092
1122	18-30	male	Yes	Left lane	5	13.291	4092
1123	18-30	male	Yes	Left lane	7	13.29	4122
1124	18-30	male	Yes	Left lane	7	13.29	4124
1125	18-30	male	Yes	Right lane	8	14.71	4138
1126	18-30	male	Yes	Left lane	10	15.75	4147
1127	18-30	male	Yes	Left lane	1	9.613	4153
1128	18-30	male	Yes	Left lane	6	13.493	4158
1129	18-30	male	Yes	Right lane	4	12.074	4167
1130	18-30	male	Yes	Left lane	6	13.493	4181
1131	18-30	male	Yes	Left lane	11	16.968	4192

1132	18-30	male	Yes	Left lane	1	9.613	4196
1133	18-30	male	Yes	Right lane	9	15.548	4219
1134	18-30	male	Yes	Right lane	12	17.17	4259
1135	18-30	male	Yes	Left lane	4	12.074	4259
1136	18-30	male	Yes	Right lane	10	15.75	4315
1137	18-30	male	Yes	Right lane	11	16.968	4336
1138	18-30	male	Yes	Left lane	11	16.968	4345
1139	18-30	male	Yes	Right lane	2	11.033	4417
1140	18-30	male	Yes	Left lane	3	11.872	4418
1141	18-30	male	Yes	Right lane	2	11.033	4419
1142	18-30	male	Yes	Left lane	3	11.872	4430
1143	18-30	male	Yes	Right lane	9	15.548	4466
1144	18-30	male	Yes	Left lane	9	15.548	4500
1145	18-30	male	Yes	Left lane	8	14.71	4514
1146	18-30	male	Yes	Right lane	2	11.033	4555
1147	18-30	male	Yes	Right lane	7	13.29	4561
1148	18-30	male	Yes	Right lane	8	14.71	4567
1149	18-30	male	Yes	Left lane	12	17.17	4613
1150	18-30	male	Yes	Right lane	6	13.493	4625
1151	18-30	male	Yes	Right lane	7	13.29	4650
1152	18-30	male	Yes	Right lane	12	17.17	4683
1153	18-30	male	Yes	Right lane	5	13.291	4745
1154	18-30	male	Yes	Right lane	8	14.71	4921
1155	18-30	male	No	Right lane	12	17.17	2839
1156	18-30	male	No	Right lane	4	12.074	3035
1157	18-30	male	No	Right lane	11	16.968	3095
1158	18-30	male	No	Left lane	8	14.71	3339
1159	18-30	male	No	Left lane	10	15.75	3517
1160	18-30	male	No	Right lane	6	13.493	3625
1161	18-30	male	No	Right lane	1	9.613	3632
1162	18-30	male	No	Left lane	9	15.548	3685
1163	18-30	male	No	Left lane	2	11.033	3717
1164	18-30	male	No	Right lane	1	9.613	3725
1165	18-30	male	No	Right lane	2	11.033	3750
1166	18-30	male	No	Left lane	1	9.613	3761
1167	18-30	male	No	Right lane	9	15.548	3785
1168	18-30	male	No	Left lane	1	9.613	3837
1169	18-30	male	No	Right lane	6	13.493	3858
1170	18-30	male	No	Left lane	4	12.074	3881
1171	18-30	male	No	Left lane	7	13.29	3938
1172	18-30	male	No	Left lane	2	11.033	3984
1173	18-30	male	No	Left lane	5	13.291	4014
1174	18-30	male	No	Right lane	1	9.613	4020
1175	18-30	male	No	Right lane	3	11.872	4031
1176	18-30	male	No	Right lane	6	13.493	4034
1177	18-30	male	No	Right lane	5	13.291	4051
1178	18-30	male	No	Left lane	6	13.493	4113
1179	18-30	male	No	Left lane	9	15.548	4126
1180	18-30	male	No	Left lane	1	9.613	4138
1181	18-30	male	No	Right lane	2	11.033	4158
1182	18-30	male	No	Left lane	7	13.29	4187
1183	18-30	male	No	Left lane	10	15.75	4203
1184	18-30	male	No	Right lane	7	13.29	4208
1185	18-30	male	No	Left lane	12	17.17	4215

1186	18-30	male	No	Right lane	8	14.71	4216
1187	18-30	male	No	Left lane	2	11.033	4221
1188	18-30	male	No	Right lane	11	16.968	4246
1189	18-30	male	No	Left lane	5	13.291	4250
1190	18-30	male	No	Right lane	8	14.71	4258
1191	18-30	male	No	Right lane	4	12.074	4260
1192	18-30	male	No	Left lane	6	13.493	4280
1193	18-30	male	No	Left lane	4	12.074	4281
1194	18-30	male	No	Right lane	2	11.033	4291
1195	18-30	male	No	Right lane	3	11.872	4291
1196	18-30	male	No	Left lane	3	11.872	4315
1197	18-30	male	No	Right lane	10	15.75	4349
1198	18-30	male	No	Right lane	12	17.17	4361
1199	18-30	male	No	Left lane	4	12.074	4387
1200	18-30	male	No	Left lane	8	14.71	4391
1201	18-30	male	No	Right lane	10	15.75	4413
1202	18-30	male	No	Right lane	4	12.074	4421
1203	18-30	male	No	Left lane	9	15.548	4429
1204	18-30	male	No	Left lane	5	13.291	4433
1205	18-30	male	No	Right lane	5	13.291	4451
1206	18-30	male	No	Right lane	5	13.291	4473
1207	18-30	male	No	Left lane	7	13.29	4479
1208	18-30	male	No	Left lane	8	14.71	4481
1209	18-30	male	No	Left lane	3	11.872	4499
1210	18-30	male	No	Right lane	8	14.71	4517
1211	18-30	male	No	Right lane	10	15.75	4522
1212	18-30	male	No	Right lane	9	15.548	4529
1213	18-30	male	No	Left lane	3	11.872	4538
1214	18-30	male	No	Left lane	12	17.17	4555
1215	18-30	male	No	Left lane	11	16.968	4559
1216	18-30	male	No	Left lane	12	17.17	4595
1217	18-30	male	No	Right lane	7	13.29	4606
1218	18-30	male	No	Right lane	3	11.872	4647
1219	18-30	male	No	Left lane	10	15.75	4681
1220	18-30	male	No	Left lane	11	16.968	4693
1221	18-30	male	No	Right lane	11	16.968	4700
1222	18-30	male	No	Left lane	11	16.968	4719
1223	18-30	male	No	Right lane	7	13.29	4733
1224	18-30	male	No	Right lane	12	17.17	4814
1225	18-30	male	No	Left lane	6	13.493	4966
1226	18-30	male	No	Right lane	9	15.548	5022
1227	18-30	female	No	Right lane	1	9.613	2792
1228	18-30	female	No	Left lane	3	11.872	2794
1229	18-30	female	No	Left lane	1	9.613	2960
1230	18-30	female	No	Left lane	3	11.872	3038
1231	18-30	female	No	Left lane	1	9.613	3059
1232	18-30	female	No	Right lane	1	9.613	3059
1233	18-30	female	No	Right lane	3	11.872	3063
1234	18-30	female	No	Left lane	4	12.074	3155
1235	18-30	female	No	Right lane	3	11.872	3295
1236	18-30	female	No	Right lane	4	12.074	3304
1237	18-30	female	No	Left lane	9	15.548	3399
1238	18-30	female	No	Left lane	1	9.613	3453
1239	18-30	female	No	Left lane	3	11.872	3519

1240	18-30	female	No	Left lane	2	11.033	3558
1241	18-30	female	No	Right lane	2	11.033	3577
1242	18-30	female	No	Right lane	4	12.074	3617
1243	18-30	female	No	Right lane	2	11.033	3781
1244	18-30	female	No	Left lane	2	11.033	3812
1245	18-30	female	No	Right lane	6	13.493	3866
1246	18-30	female	No	Left lane	11	16.968	3881
1247	18-30	female	No	Right lane	8	14.71	3897
1248	18-30	female	No	Left lane	9	15.548	3915
1249	18-30	female	No	Left lane	4	12.074	3926
1250	18-30	female	No	Right lane	7	13.29	3931
1251	18-30	female	No	Left lane	9	15.548	3948
1252	18-30	female	No	Left lane	7	13.29	4012
1253	18-30	female	No	Left lane	5	13.291	4018
1254	18-30	female	No	Right lane	6	13.493	4040
1255	18-30	female	No	Right lane	12	17.17	4071
1256	18-30	female	No	Right lane	10	15.75	4097
1257	18-30	female	No	Right lane	5	13.291	4132
1258	18-30	female	No	Right lane	11	16.968	4185
1259	18-30	female	No	Right lane	4	12.074	4187
1260	18-30	female	No	Left lane	2	11.033	4199
1261	18-30	female	No	Left lane	6	13.493	4215
1262	18-30	female	No	Left lane	11	16.968	4218
1263	18-30	female	No	Right lane	12	17.17	4227
1264	18-30	female	No	Left lane	8	14.71	4239
1265	18-30	female	No	Left lane	12	17.17	4244
1266	18-30	female	No	Right lane	9	15.548	4246
1267	18-30	female	No	Left lane	7	13.29	4249
1268	18-30	female	No	Right lane	5	13.291	4271
1269	18-30	female	No	Left lane	5	13.291	4291
1270	18-30	female	No	Left lane	4	12.074	4305
1271	18-30	female	No	Left lane	6	13.493	4314
1272	18-30	female	No	Left lane	8	14.71	4346
1273	18-30	female	No	Right lane	8	14.71	4380
1274	18-30	female	No	Left lane	10	15.75	4389
1275	18-30	female	No	Left lane	5	13.291	4414
1276	18-30	female	No	Left lane	12	17.17	4426
1277	18-30	female	No	Right lane	2	11.033	4450
1278	18-30	female	No	Right lane	9	15.548	4466
1279	18-30	female	No	Left lane	11	16.968	4479
1280	18-30	female	No	Right lane	10	15.75	4520
1281	18-30	female	No	Left lane	10	15.75	4548
1282	18-30	female	No	Right lane	1	9.613	4569
1283	18-30	female	No	Left lane	8	14.71	4599
1284	18-30	female	No	Right lane	11	16.968	4648
1285	18-30	female	No	Right lane	8	14.71	4683
1286	18-30	female	No	Right lane	3	11.872	4722
1287	18-30	female	No	Right lane	11	16.968	4740
1288	18-30	female	No	Left lane	12	17.17	4779
1289	18-30	female	No	Right lane	7	13.29	4786
1290	18-30	female	No	Right lane	5	13.291	4833
1291	18-30	female	No	Right lane	9	15.548	4894
1292	18-30	female	No	Right lane	10	15.75	4903
1293	18-30	female	No	Right lane	7	13.29	4937

1294	18-30	female	No	Left lane	7	13.29	4951
1295	18-30	female	No	Right lane	6	13.493	4960
1296	18-30	female	No	Left lane	6	13.493	4997
1297	18-30	female	No	Right lane	12	17.17	4999
1298	18-30	female	No	Left lane	10	15.75	5327
1299	18-30	male	No	Left lane	6	13.493	3494
1300	18-30	male	No	Left lane	10	15.75	3634
1301	18-30	male	No	Right lane	1	9.613	3681
1302	18-30	male	No	Right lane	3	11.872	3718
1303	18-30	male	No	Right lane	12	17.17	3747
1304	18-30	male	No	Right lane	8	14.71	3871
1305	18-30	male	No	Right lane	1	9.613	3902
1306	18-30	male	No	Right lane	8	14.71	3948
1307	18-30	male	No	Left lane	4	12.074	3961
1308	18-30	male	No	Left lane	1	9.613	3965
1309	18-30	male	No	Right lane	4	12.074	3983
1310	18-30	male	No	Left lane	8	14.71	3988
1311	18-30	male	No	Left lane	2	11.033	4006
1312	18-30	male	No	Right lane	12	17.17	4027
1313	18-30	male	No	Right lane	4	12.074	4044
1314	18-30	male	No	Right lane	12	17.17	4047
1315	18-30	male	No	Left lane	7	13.29	4058
1316	18-30	male	No	Left lane	9	15.548	4102
1317	18-30	male	No	Left lane	1	9.613	4103
1318	18-30	male	No	Left lane	8	14.71	4116
1319	18-30	male	No	Right lane	6	13.493	4118
1320	18-30	male	No	Left lane	2	11.033	4135
1321	18-30	male	No	Left lane	11	16.968	4139
1322	18-30	male	No	Right lane	6	13.493	4155
1323	18-30	male	No	Left lane	5	13.291	4158
1324	18-30	male	No	Left lane	1	9.613	4181
1325	18-30	male	No	Left lane	5	13.291	4203
1326	18-30	male	No	Left lane	2	11.033	4215
1327	18-30	male	No	Left lane	4	12.074	4225
1328	18-30	male	No	Left lane	7	13.29	4226
1329	18-30	male	No	Right lane	2	11.033	4252
1330	18-30	male	No	Left lane	10	15.75	4260
1331	18-30	male	No	Left lane	10	15.75	4277
1332	18-30	male	No	Right lane	11	16.968	4281
1333	18-30	male	No	Right lane	1	9.613	4302
1334	18-30	male	No	Right lane	10	15.75	4338
1335	18-30	male	No	Right lane	6	13.493	4344
1336	18-30	male	No	Left lane	6	13.493	4372
1337	18-30	male	No	Right lane	3	11.872	4376
1338	18-30	male	No	Left lane	11	16.968	4386
1339	18-30	male	No	Right lane	10	15.75	4391
1340	18-30	male	No	Right lane	11	16.968	4397
1341	18-30	male	No	Left lane	12	17.17	4451
1342	18-30	male	No	Left lane	6	13.493	4455
1343	18-30	male	No	Left lane	7	13.29	4465
1344	18-30	male	No	Right lane	2	11.033	4467
1345	18-30	male	No	Left lane	3	11.872	4504
1346	18-30	male	No	Right lane	5	13.291	4507
1347	18-30	male	No	Right lane	10	15.75	4518

1348	18-30	male	No	Right lane	9	15.548	4531
1349	18-30	male	No	Left lane	5	13.291	4535
1350	18-30	male	No	Left lane	11	16.968	4580
1351	18-30	male	No	Left lane	3	11.872	4581
1352	18-30	male	No	Left lane	12	17.17	4591
1353	18-30	male	No	Left lane	12	17.17	4602
1354	18-30	male	No	Left lane	4	12.074	4628
1355	18-30	male	No	Right lane	5	13.291	4632
1356	18-30	male	No	Right lane	7	13.29	4648
1357	18-30	male	No	Right lane	3	11.872	4681
1358	18-30	male	No	Right lane	2	11.033	4683
1359	18-30	male	No	Right lane	7	13.29	4693
1360	18-30	male	No	Right lane	4	12.074	4802
1361	18-30	male	No	Right lane	7	13.29	4804
1362	18-30	male	No	Left lane	9	15.548	4806
1363	18-30	male	No	Right lane	9	15.548	4818
1364	18-30	male	No	Right lane	5	13.291	4853
1365	18-30	male	No	Left lane	9	15.548	4871
1366	18-30	male	No	Left lane	3	11.872	4881
1367	18-30	male	No	Right lane	8	14.71	4893
1368	18-30	male	No	Left lane	8	14.71	4931
1369	18-30	male	No	Right lane	11	16.968	4946
1370	18-30	male	No	Right lane	9	15.548	5184
1371	18-30	male	Yes	Right lane	3	11.872	2870
1372	18-30	male	Yes	Left lane	10	15.75	2921
1373	18-30	male	Yes	Left lane	3	11.872	3036
1374	18-30	male	Yes	Right lane	10	15.75	3080
1375	18-30	male	Yes	Right lane	5	13.291	3230
1376	18-30	male	Yes	Left lane	1	9.613	3248
1377	18-30	male	Yes	Right lane	2	11.033	3251
1378	18-30	male	Yes	Right lane	10	15.75	3254
1379	18-30	male	Yes	Left lane	1	9.613	3259
1380	18-30	male	Yes	Right lane	1	9.613	3288
1381	18-30	male	Yes	Right lane	4	12.074	3297
1382	18-30	male	Yes	Left lane	12	17.17	3352
1383	18-30	male	Yes	Right lane	7	13.29	3413
1384	18-30	male	Yes	Right lane	5	13.291	3446
1385	18-30	male	Yes	Left lane	5	13.291	3446
1386	18-30	male	Yes	Left lane	4	12.074	3457
1387	18-30	male	Yes	Right lane	3	11.872	3464
1388	18-30	male	Yes	Left lane	6	13.493	3488
1389	18-30	male	Yes	Left lane	10	15.75	3494
1390	18-30	male	Yes	Right lane	12	17.17	3547
1391	18-30	male	Yes	Left lane	1	9.613	3547
1392	18-30	male	Yes	Right lane	12	17.17	3550
1393	18-30	male	Yes	Right lane	10	15.75	3577
1394	18-30	male	Yes	Left lane	5	13.291	3579
1395	18-30	male	Yes	Left lane	6	13.493	3591
1396	18-30	male	Yes	Right lane	5	13.291	3595
1397	18-30	male	Yes	Right lane	11	16.968	3610
1398	18-30	male	Yes	Left lane	3	11.872	3634
1399	18-30	male	Yes	Left lane	5	13.291	3647
1400	18-30	male	Yes	Left lane	10	15.75	3658
1401	18-30	male	Yes	Right lane	8	14.71	3672

1402	18-30	male	Yes	Left lane	3	11.872	3672
1403	18-30	male	Yes	Left lane	7	13.29	3673
1404	18-30	male	Yes	Right lane	11	16.968	3684
1405	18-30	male	Yes	Right lane	9	15.548	3686
1406	18-30	male	Yes	Left lane	2	11.033	3698
1407	18-30	male	Yes	Right lane	2	11.033	3717
1408	18-30	male	Yes	Right lane	8	14.71	3728
1409	18-30	male	Yes	Right lane	9	15.548	3729
1410	18-30	male	Yes	Right lane	4	12.074	3738
1411	18-30	male	Yes	Left lane	2	11.033	3752
1412	18-30	male	Yes	Left lane	4	12.074	3753
1413	18-30	male	Yes	Right lane	6	13.493	3789
1414	18-30	male	Yes	Left lane	8	14.71	3795
1415	18-30	male	Yes	Left lane	9	15.548	3838
1416	18-30	male	Yes	Left lane	9	15.548	3847
1417	18-30	male	Yes	Right lane	6	13.493	3849
1418	18-30	male	Yes	Left lane	8	14.71	3864
1419	18-30	male	Yes	Left lane	2	11.033	3866
1420	18-30	male	Yes	Right lane	3	11.872	3885
1421	18-30	male	Yes	Right lane	6	13.493	3919
1422	18-30	male	Yes	Right lane	2	11.033	3925
1423	18-30	male	Yes	Left lane	6	13.493	3927
1424	18-30	male	Yes	Right lane	1	9.613	3940
1425	18-30	male	Yes	Right lane	9	15.548	3992
1426	18-30	male	Yes	Left lane	12	17.17	4017
1427	18-30	male	Yes	Left lane	7	13.29	4033
1428	18-30	male	Yes	Right lane	8	14.71	4070
1429	18-30	male	Yes	Right lane	11	16.968	4091
1430	18-30	male	Yes	Left lane	9	15.548	4092
1431	18-30	male	Yes	Left lane	11	16.968	4095
1432	18-30	male	Yes	Right lane	12	17.17	4096
1433	18-30	male	Yes	Right lane	7	13.29	4101
1434	18-30	male	Yes	Left lane	11	16.968	4171
1435	18-30	male	Yes	Left lane	7	13.29	4230
1436	18-30	male	Yes	Left lane	8	14.71	4332
1437	18-30	male	Yes	Right lane	7	13.29	4437
1438	18-30	male	Yes	Left lane	11	16.968	4699
1439	18-30	male	Yes	Right lane	4	12.074	4769
