# Supplementary material for: Setting method of exit advance guide signs in mountainous expressway tunnel based on information quantization theory
Source: PLoS One. 2023 Feb 16;18(2):e0281842. doi: 10.1371/journal.pone.0281842 (PMC9934451; doi:10.1371/journal.pone.0281842)
Supplement: S2 Table — (PDF) [file pone.0281842.s002.pdf]

Summary of statistical data of three-character place names test

number	Age	Gender	experience	Lane number	combination	amount of information	reaction time
1	18-30	Male	Yes	Right lane	2	15.612	2800
2	18-30	Male	Yes	Left lane	1	14.192	2866
3	18-30	Male	Yes	Right lane	1	14.192	3038
4	18-30	Male	Yes	Right lane	3	16.45	3048
5	18-30	Male	Yes	Right lane	1	14.192	3125
6	18-30	Male	Yes	Right lane	1	14.192	3266
7	18-30	Male	Yes	Right lane	3	16.45	3294
8	18-30	Male	Yes	Right lane	3	16.45	3412
9	18-30	Male	Yes	Right lane	4	16.652	3426
10	18-30	Male	Yes	Left lane	2	15.612	3471
11	18-30	Male	Yes	Right lane	6	18.072	3471
12	18-30	Male	Yes	Right lane	3	16.45	3620
13	18-30	Male	Yes	Left lane	9	20.944	3660
14	18-30	Male	Yes	Right lane	2	15.612	3661
15	18-30	Male	Yes	Left lane	6	18.072	3664
16	18-30	Male	Yes	Left lane	4	16.652	3667
17	18-30	Male	Yes	Right lane	4	16.652	3706
18	18-30	Male	Yes	Right lane	8	20.105	3749
19	18-30	Male	Yes	Left lane	4	16.652	3771
20	18-30	Male	Yes	Left lane	7	18.685	3806
21	18-30	Male	Yes	Right lane	2	15.612	3861
22	18-30	Male	Yes	Left lane	3	16.45	3880
23	18-30	Male	Yes	Left lane	1	14.192	3948
24	18-30	Male	Yes	Right lane	11	22.363	3952
25	18-30	Male	Yes	Right lane	11	22.363	3960
26	18-30	Male	Yes	Right lane	9	20.944	3988
27	18-30	Male	Yes	Left lane	5	17.87	4018
28	18-30	Male	Yes	Left lane	7	18.685	4071
29	18-30	Male	Yes	Left lane	5	17.87	4076
30	18-30	Male	Yes	Right lane	4	16.652	4097
31	18-30	Male	Yes	Right lane	6	18.072	4125
32	18-30	Male	Yes	Right lane	7	18.685	4129
33	18-30	Male	Yes	Right lane	12	22.565	4134
34	18-30	Male	Yes	Left lane	2	15.612	4167
35	18-30	Male	Yes	Right lane	4	16.652	4172
36	18-30	Male	Yes	Right lane	7	18.685	4195
37	18-30	Male	Yes	Right lane	6	18.072	4212
38	18-30	Male	Yes	Left lane	5	17.87	4234
39	18-30	Male	Yes	Left lane	11	22.363	4262
40	18-30	Male	Yes	Right lane	12	22.565	4268
41	18-30	Male	Yes	Left lane	6	18.072	4277
42	18-30	Male	Yes	Left lane	8	20.105	4279
43	18-30	Male	Yes	Left lane	8	20.105	4306
44	18-30	Male	Yes	Right lane	10	21.146	4339
45	18-30	Male	Yes	Right lane	1	14.192	4345
46	18-30	Male	Yes	Left lane	3	16.45	4350
47	18-30	Male	Yes	Left lane	7	18.685	4354
48	18-30	Male	Yes	Left lane	11	22.363	4355
49	18-30	Male	Yes	Left lane	10	21.146	4363
50	18-30	Male	Yes	Right lane	5	17.87	4402
51	18-30	Male	Yes	Left lane	10	21.146	4437

52	18-30	Male	Yes	Right lane	5	17.87	4459
53	18-30	Male	Yes	Left lane	12	22.565	4519
54	18-30	Male	Yes	Left lane	10	21.146	4529
55	18-30	Male	Yes	Right lane	9	20.944	4552
56	18-30	Male	Yes	Left lane	2	15.612	4580
57	18-30	Male	Yes	Left lane	3	16.45	4587
58	18-30	Male	Yes	Right lane	9	20.944	4598
59	18-30	Male	Yes	Left lane	1	14.192	4603
60	18-30	Male	Yes	Left lane	9	20.944	4638
61	18-30	Male	Yes	Left lane	10	21.146	4653
62	18-30	Male	Yes	Left lane	5	17.87	4664
63	18-30	Male	Yes	Left lane	12	22.565	4666
64	18-30	Male	Yes	Right lane	12	22.565	4680
65	18-30	Male	Yes	Right lane	11	22.363	4754
66	18-30	Male	Yes	Right lane	5	17.87	4784
67	18-30	Male	Yes	Left lane	8	20.105	4802
68	18-30	Male	Yes	Right lane	6	18.072	4805
69	18-30	Male	Yes	Left lane	7	18.685	4813
70	18-30	Male	Yes	Left lane	2	15.612	4824
71	18-30	Male	Yes	Right lane	8	20.105	4836
72	18-30	Male	Yes	Right lane	10	21.146	4881
73	18-30	Male	Yes	Left lane	4	16.652	4884
74	18-30	Male	Yes	Right lane	8	20.105	4926
75	18-30	Male	Yes	Left lane	9	20.944	4965
76	18-30	Male	Yes	Left lane	9	20.944	5061
77	18-30	Male	Yes	Left lane	6	18.072	5070
78	18-30	Male	Yes	Left lane	8	20.105	5087
79	18-30	Male	Yes	Right lane	11	22.363	5117
80	18-30	Male	Yes	Right lane	7	18.685	5215
81	18-30	Male	Yes	Left lane	11	22.363	5248
82	18-30	Male	Yes	Right lane	10	21.146	5263
83	18-30	Male	Yes	Right lane	12	22.565	5305
84	18-30	Female	NO	Right lane	3	16.45	2584
85	18-30	Female	NO	Left lane	8	20.105	3015
86	18-30	Female	NO	Right lane	3	16.45	3054
87	18-30	Female	NO	Right lane	1	14.192	3213
88	18-30	Female	NO	Right lane	4	16.652	3270
89	18-30	Female	NO	Right lane	3	16.45	3404
90	18-30	Female	NO	Right lane	9	20.944	3451
91	18-30	Female	NO	Left lane	4	16.652	3505
92	18-30	Female	NO	Left lane	3	16.45	3526
93	18-30	Female	NO	Right lane	10	21.146	3546
94	18-30	Female	NO	Left lane	12	22.565	3555
95	18-30	Female	NO	Right lane	6	18.072	3580
96	18-30	Female	NO	Left lane	3	16.45	3612
97	18-30	Female	NO	Right lane	4	16.652	3616
98	18-30	Female	NO	Left lane	1	14.192	3618
99	18-30	Female	NO	Left lane	7	18.685	3619
100	18-30	Female	NO	Right lane	4	16.652	3632
101	18-30	Female	NO	Left lane	4	16.652	3671
102	18-30	Female	NO	Left lane	2	15.612	3673
103	18-30	Female	NO	Left lane	7	18.685	3680
104	18-30	Female	NO	Right lane	6	18.072	3700
105	18-30	Female	NO	Right lane	12	22.565	3780

106	18-30	Female	NO	Left lane	5	17.87	3781
107	18-30	Female	NO	Left lane	12	22.565	3786
108	18-30	Female	NO	Right lane	1	14.192	3787
109	18-30	Female	NO	Right lane	12	22.565	3788
110	18-30	Female	NO	Left lane	3	16.45	3833
111	18-30	Female	NO	Left lane	1	14.192	3836
112	18-30	Female	NO	Left lane	1	14.192	3844
113	18-30	Female	NO	Right lane	7	18.685	3855
114	18-30	Female	NO	Left lane	5	17.87	3884
115	18-30	Female	NO	Right lane	11	22.363	3915
116	18-30	Female	NO	Left lane	11	22.363	3922
117	18-30	Female	NO	Right lane	7	18.685	3929
118	18-30	Female	NO	Left lane	6	18.072	3930
119	18-30	Female	NO	Left lane	11	22.363	3970
120	18-30	Female	NO	Right lane	12	22.565	3983
121	18-30	Female	NO	Left lane	7	18.685	3993
122	18-30	Female	NO	Left lane	5	17.87	4021
123	18-30	Female	NO	Left lane	12	22.565	4028
124	18-30	Female	NO	Left lane	9	20.944	4034
125	18-30	Female	NO	Left lane	10	21.146	4046
126	18-30	Female	NO	Right lane	2	15.612	4047
127	18-30	Female	NO	Right lane	6	18.072	4049
128	18-30	Female	NO	Right lane	2	15.612	4052
129	18-30	Female	NO	Right lane	11	22.363	4058
130	18-30	Female	NO	Right lane	1	14.192	4059
131	18-30	Female	NO	Left lane	10	21.146	4091
132	18-30	Female	NO	Left lane	8	20.105	4093
133	18-30	Female	NO	Right lane	5	17.87	4097
134	18-30	Female	NO	Right lane	10	21.146	4098
135	18-30	Female	NO	Left lane	2	15.612	4106
136	18-30	Female	NO	Right lane	5	17.87	4106
137	18-30	Female	NO	Left lane	6	18.072	4114
138	18-30	Female	NO	Left lane	10	21.146	4117
139	18-30	Female	NO	Left lane	9	20.944	4124
140	18-30	Female	NO	Right lane	11	22.363	4146
141	18-30	Female	NO	Left lane	2	15.612	4147
142	18-30	Female	NO	Left lane	9	20.944	4157
143	18-30	Female	NO	Left lane	4	16.652	4162
144	18-30	Female	NO	Right lane	10	21.146	4186
145	18-30	Female	NO	Right lane	7	18.685	4216
146	18-30	Female	NO	Left lane	8	20.105	4220
147	18-30	Female	NO	Right lane	8	20.105	4263
148	18-30	Female	NO	Right lane	5	17.87	4274
149	18-30	Female	NO	Right lane	2	15.612	4282
150	18-30	Female	NO	Right lane	8	20.105	4334
151	18-30	Female	NO	Right lane	9	20.944	4335
152	18-30	Female	NO	Right lane	8	20.105	4335
153	18-30	Female	NO	Left lane	6	18.072	4449
154	18-30	Female	NO	Left lane	11	22.363	4487
155	18-30	Female	NO	Right lane	9	20.944	4512
156	18-30	Male	NO	Right lane	10	21.146	3014
157	18-30	Male	NO	Left lane	12	22.565	3172
158	18-30	Male	NO	Right lane	4	16.652	3248
159	18-30	Male	NO	Left lane	1	14.192	3391

160	18-30	Male	NO	Right lane	1	14.192	3402
161	18-30	Male	NO	Left lane	6	18.072	3522
162	18-30	Male	NO	Right lane	7	18.685	3527
163	18-30	Male	NO	Left lane	3	16.45	3538
164	18-30	Male	NO	Left lane	4	16.652	3581
165	18-30	Male	NO	Left lane	4	16.652	3597
166	18-30	Male	NO	Right lane	3	16.45	3647
167	18-30	Male	NO	Left lane	11	22.363	3666
168	18-30	Male	NO	Left lane	12	22.565	3696
169	18-30	Male	NO	Left lane	1	14.192	3715
170	18-30	Male	NO	Left lane	3	16.45	3732
171	18-30	Male	NO	Left lane	8	20.105	3761
172	18-30	Male	NO	Right lane	5	17.87	3768
173	18-30	Male	NO	Right lane	9	20.944	3785
174	18-30	Male	NO	Left lane	6	18.072	3802
175	18-30	Male	NO	Left lane	10	21.146	3814
176	18-30	Male	NO	Right lane	2	15.612	3825
177	18-30	Male	NO	Right lane	5	17.87	3885
178	18-30	Male	NO	Left lane	3	16.45	3954
179	18-30	Male	NO	Right lane	2	15.612	3987
180	18-30	Male	NO	Right lane	8	20.105	3994
181	18-30	Male	NO	Left lane	11	22.363	4024
182	18-30	Male	NO	Left lane	8	20.105	4040
183	18-30	Male	NO	Right lane	1	14.192	4050
184	18-30	Male	NO	Left lane	5	17.87	4055
185	18-30	Male	NO	Right lane	12	22.565	4080
186	18-30	Male	NO	Left lane	4	16.652	4147
187	18-30	Male	NO	Right lane	5	17.87	4159
188	18-30	Male	NO	Left lane	5	17.87	4181
189	18-30	Male	NO	Right lane	11	22.363	4186
190	18-30	Male	NO	Right lane	3	16.45	4186
191	18-30	Male	NO	Right lane	7	18.685	4216
192	18-30	Male	NO	Right lane	6	18.072	4228
193	18-30	Male	NO	Right lane	6	18.072	4239
194	18-30	Male	NO	Left lane	2	15.612	4286
195	18-30	Male	NO	Right lane	9	20.944	4297
196	18-30	Male	NO	Left lane	2	15.612	4299
197	18-30	Male	NO	Left lane	5	17.87	4336
198	18-30	Male	NO	Right lane	8	20.105	4367
199	18-30	Male	NO	Left lane	11	22.363	4379
200	18-30	Male	NO	Left lane	9	20.944	4427
201	18-30	Male	NO	Right lane	9	20.944	4434
202	18-30	Male	NO	Left lane	1	14.192	4438
203	18-30	Male	NO	Right lane	12	22.565	4452
204	18-30	Male	NO	Right lane	10	21.146	4469
205	18-30	Male	NO	Right lane	6	18.072	4479
206	18-30	Male	NO	Left lane	8	20.105	4483
207	18-30	Male	NO	Left lane	2	15.612	4486
208	18-30	Male	NO	Right lane	12	22.565	4487
209	18-30	Male	NO	Right lane	11	22.363	4501
210	18-30	Male	NO	Right lane	3	16.45	4514
211	18-30	Male	NO	Right lane	4	16.652	4519
212	18-30	Male	NO	Left lane	7	18.685	4546
213	18-30	Male	NO	Left lane	10	21.146	4585

214	18-30	Male	NO	Right lane	4	16.652	4594
215	18-30	Male	NO	Left lane	6	18.072	4637
216	18-30	Male	NO	Left lane	9	20.944	4640
217	18-30	Male	NO	Left lane	10	21.146	4649
218	18-30	Male	NO	Left lane	7	18.685	4663
219	18-30	Male	NO	Left lane	12	22.565	4682
220	18-30	Male	NO	Right lane	10	21.146	4720
221	18-30	Male	NO	Left lane	7	18.685	4725
222	18-30	Male	NO	Right lane	2	15.612	4746
223	18-30	Male	NO	Left lane	9	20.944	4749
224	18-30	Male	NO	Right lane	7	18.685	4881
225	18-30	Male	NO	Right lane	11	22.363	4896
226	18-30	Male	NO	Right lane	8	20.105	5025
227	18-30	Male	NO	Right lane	1	14.192	5155
228	18-30	Male	NO	Right lane	4	16.652	3482
229	18-30	Male	NO	Right lane	4	16.652	3615
230	18-30	Male	NO	Left lane	9	20.944	3625
231	18-30	Male	NO	Right lane	3	16.45	3635
232	18-30	Male	NO	Left lane	5	17.87	3672
233	18-30	Male	NO	Left lane	10	21.146	3699
234	18-30	Male	NO	Left lane	9	20.944	3763
235	18-30	Male	NO	Right lane	12	22.565	3812
236	18-30	Male	NO	Left lane	11	22.363	3826
237	18-30	Male	NO	Left lane	12	22.565	3840
238	18-30	Male	NO	Left lane	7	18.685	3892
239	18-30	Male	NO	Right lane	3	16.45	3915
240	18-30	Male	NO	Right lane	9	20.944	3920
241	18-30	Male	NO	Right lane	2	15.612	3936
242	18-30	Male	NO	Right lane	11	22.363	3938
243	18-30	Male	NO	Left lane	10	21.146	3967
244	18-30	Male	NO	Right lane	5	17.87	3983
245	18-30	Male	NO	Right lane	3	16.45	3994
246	18-30	Male	NO	Left lane	6	18.072	3996
247	18-30	Male	NO	Right lane	6	18.072	3998
248	18-30	Male	NO	Left lane	6	18.072	4017
249	18-30	Male	NO	Left lane	7	18.685	4031
250	18-30	Male	NO	Right lane	12	22.565	4044
251	18-30	Male	NO	Right lane	4	16.652	4046
252	18-30	Male	NO	Left lane	1	14.192	4047
253	18-30	Male	NO	Right lane	1	14.192	4066
254	18-30	Male	NO	Left lane	11	22.363	4071
255	18-30	Male	NO	Right lane	6	18.072	4073
256	18-30	Male	NO	Left lane	8	20.105	4086
257	18-30	Male	NO	Left lane	4	16.652	4099
258	18-30	Male	NO	Left lane	8	20.105	4102
259	18-30	Male	NO	Right lane	6	18.072	4136
260	18-30	Male	NO	Left lane	12	22.565	4146
261	18-30	Male	NO	Left lane	7	18.685	4147
262	18-30	Male	NO	Left lane	5	17.87	4149
263	18-30	Male	NO	Right lane	1	14.192	4153
264	18-30	Male	NO	Right lane	1	14.192	4155
265	18-30	Male	NO	Left lane	5	17.87	4159
266	18-30	Male	NO	Left lane	12	22.565	4162
267	18-30	Male	NO	Left lane	6	18.072	4181

268	18-30	Male	NO	Right lane	11	22.363	4184
269	18-30	Male	NO	Left lane	3	16.45	4195
270	18-30	Male	NO	Left lane	4	16.652	4198
271	18-30	Male	NO	Right lane	8	20.105	4199
272	18-30	Male	NO	Left lane	1	14.192	4225
273	18-30	Male	NO	Left lane	3	16.45	4247
274	18-30	Male	NO	Left lane	4	16.652	4251
275	18-30	Male	NO	Right lane	12	22.565	4268
276	18-30	Male	NO	Right lane	11	22.363	4284
277	18-30	Male	NO	Right lane	5	17.87	4302
278	18-30	Male	NO	Left lane	11	22.363	4325
279	18-30	Male	NO	Left lane	9	20.944	4326
280	18-30	Male	NO	Left lane	1	14.192	4331
281	18-30	Male	NO	Right lane	5	17.87	4351
282	18-30	Male	NO	Left lane	2	15.612	4363
283	18-30	Male	NO	Left lane	2	15.612	4427
284	18-30	Male	NO	Right lane	10	21.146	4436
285	18-30	Male	NO	Right lane	8	20.105	4440
286	18-30	Male	NO	Right lane	7	18.685	4457
287	18-30	Male	NO	Left lane	2	15.612	4464
288	18-30	Male	NO	Left lane	3	16.45	4486
289	18-30	Male	NO	Left lane	10	21.146	4515
290	18-30	Male	NO	Right lane	7	18.685	4529
291	18-30	Male	NO	Right lane	2	15.612	4558
292	18-30	Male	NO	Right lane	9	20.944	4576
293	18-30	Male	NO	Right lane	2	15.612	4610
294	18-30	Male	NO	Right lane	8	20.105	4614
295	18-30	Male	NO	Right lane	10	21.146	4637
296	18-30	Male	NO	Right lane	7	18.685	4666
297	18-30	Male	NO	Right lane	9	20.944	4670
298	18-30	Male	NO	Left lane	8	20.105	4745
299	18-30	Male	NO	Right lane	10	21.146	4758
300	18-30	Female	Yes	Left lane	3	16.45	2462
301	18-30	Female	Yes	Left lane	2	15.612	3049
302	18-30	Female	Yes	Left lane	4	16.652	3094
303	18-30	Female	Yes	Left lane	1	14.192	3146
304	18-30	Female	Yes	Left lane	1	14.192	3233
305	18-30	Female	Yes	Left lane	2	15.612	3285
306	18-30	Female	Yes	Right lane	1	14.192	3325
307	18-30	Female	Yes	Left lane	5	17.87	3366
308	18-30	Female	Yes	Left lane	3	16.45	3391
309	18-30	Female	Yes	Right lane	6	18.072	3427
310	18-30	Female	Yes	Right lane	2	15.612	3427
311	18-30	Female	Yes	Left lane	6	18.072	3438
312	18-30	Female	Yes	Left lane	4	16.652	3613
313	18-30	Female	Yes	Right lane	4	16.652	3655
314	18-30	Female	Yes	Left lane	6	18.072	3715
315	18-30	Female	Yes	Left lane	1	14.192	3726
316	18-30	Female	Yes	Right lane	2	15.612	3730
317	18-30	Female	Yes	Right lane	8	20.105	3829
318	18-30	Female	Yes	Right lane	4	16.652	3897
319	18-30	Female	Yes	Left lane	8	20.105	3901
320	18-30	Female	Yes	Right lane	9	20.944	3913
321	18-30	Female	Yes	Right lane	6	18.072	3948

322	18-30	Female	Yes	Right lane	10	21.146	3983
323	18-30	Female	Yes	Right lane	3	16.45	3985
324	18-30	Female	Yes	Right lane	3	16.45	4012
325	18-30	Female	Yes	Left lane	11	22.363	4024
326	18-30	Female	Yes	Left lane	10	21.146	4033
327	18-30	Female	Yes	Right lane	7	18.685	4067
328	18-30	Female	Yes	Right lane	6	18.072	4079
329	18-30	Female	Yes	Right lane	12	22.565	4080
330	18-30	Female	Yes	Left lane	8	20.105	4084
331	18-30	Female	Yes	Left lane	12	22.565	4122
332	18-30	Female	Yes	Left lane	5	17.87	4132
333	18-30	Female	Yes	Right lane	9	20.944	4188
334	18-30	Female	Yes	Left lane	11	22.363	4206
335	18-30	Female	Yes	Left lane	12	22.565	4226
336	18-30	Female	Yes	Left lane	4	16.652	4231
337	18-30	Female	Yes	Right lane	2	15.612	4281
338	18-30	Female	Yes	Right lane	1	14.192	4296
339	18-30	Female	Yes	Left lane	6	18.072	4340
340	18-30	Female	Yes	Right lane	1	14.192	4358
341	18-30	Female	Yes	Left lane	8	20.105	4358
342	18-30	Female	Yes	Right lane	5	17.87	4368
343	18-30	Female	Yes	Left lane	3	16.45	4371
344	18-30	Female	Yes	Right lane	8	20.105	4381
345	18-30	Female	Yes	Right lane	5	17.87	4449
346	18-30	Female	Yes	Left lane	10	21.146	4460
347	18-30	Female	Yes	Left lane	9	20.944	4479
348	18-30	Female	Yes	Right lane	9	20.944	4540
349	18-30	Female	Yes	Right lane	3	16.45	4557
350	18-30	Female	Yes	Left lane	12	22.565	4592
351	18-30	Female	Yes	Left lane	7	18.685	4599
352	18-30	Female	Yes	Left lane	10	21.146	4626
353	18-30	Female	Yes	Left lane	5	17.87	4632
354	18-30	Female	Yes	Right lane	11	22.363	4649
355	18-30	Female	Yes	Right lane	10	21.146	4651
356	18-30	Female	Yes	Left lane	7	18.685	4651
357	18-30	Female	Yes	Right lane	7	18.685	4663
358	18-30	Female	Yes	Left lane	2	15.612	4671
359	18-30	Female	Yes	Right lane	7	18.685	4683
360	18-30	Female	Yes	Right lane	11	22.363	4725
361	18-30	Female	Yes	Left lane	9	20.944	4814
362	18-30	Female	Yes	Right lane	12	22.565	4815
363	18-30	Female	Yes	Right lane	5	17.87	4864
364	18-30	Female	Yes	Right lane	8	20.105	4866
365	18-30	Female	Yes	Left lane	11	22.363	4947
366	18-30	Female	Yes	Right lane	10	21.146	4963
367	18-30	Female	Yes	Right lane	11	22.363	5002
368	18-30	Female	Yes	Right lane	12	22.565	5024
369	18-30	Female	Yes	Left lane	9	20.944	5088
370	18-30	Male	NO	Right lane	3	16.45	2338
371	18-30	Male	NO	Right lane	4	16.652	2653
372	18-30	Male	NO	Left lane	1	14.192	2853
373	18-30	Male	NO	Right lane	2	15.612	3294
374	18-30	Male	NO	Right lane	1	14.192	3368
375	18-30	Male	NO	Right lane	1	14.192	3404

376	18-30	Male	NO	Left lane	1	14.192	3443
377	18-30	Male	NO	Right lane	4	16.652	3535
378	18-30	Male	NO	Right lane	1	14.192	3556
379	18-30	Male	NO	Left lane	3	16.45	3572
380	18-30	Male	NO	Right lane	4	16.652	3591
381	18-30	Male	NO	Right lane	10	21.146	3647
382	18-30	Male	NO	Left lane	6	18.072	3683
383	18-30	Male	NO	Left lane	4	16.652	3717
384	18-30	Male	NO	Right lane	3	16.45	3717
385	18-30	Male	NO	Left lane	1	14.192	3781
386	18-30	Male	NO	Right lane	2	15.612	3843
387	18-30	Male	NO	Left lane	6	18.072	3851
388	18-30	Male	NO	Left lane	4	16.652	3851
389	18-30	Male	NO	Left lane	6	18.072	3877
390	18-30	Male	NO	Right lane	5	17.87	3891
391	18-30	Male	NO	Left lane	2	15.612	3918
392	18-30	Male	NO	Left lane	5	17.87	3921
393	18-30	Male	NO	Right lane	6	18.072	3924
394	18-30	Male	NO	Right lane	5	17.87	3933
395	18-30	Male	NO	Left lane	5	17.87	3936
396	18-30	Male	NO	Right lane	3	16.45	3939
397	18-30	Male	NO	Left lane	5	17.87	3951
398	18-30	Male	NO	Left lane	9	20.944	3964
399	18-30	Male	NO	Right lane	8	20.105	3965
400	18-30	Male	NO	Right lane	11	22.363	3981
401	18-30	Male	NO	Left lane	10	21.146	3984
402	18-30	Male	NO	Left lane	4	16.652	3984
403	18-30	Male	NO	Right lane	12	22.565	4025
404	18-30	Male	NO	Left lane	3	16.45	4026
405	18-30	Male	NO	Right lane	9	20.944	4047
406	18-30	Male	NO	Left lane	8	20.105	4106
407	18-30	Male	NO	Left lane	12	22.565	4112
408	18-30	Male	NO	Right lane	7	18.685	4146
409	18-30	Male	NO	Right lane	6	18.072	4148
410	18-30	Male	NO	Left lane	12	22.565	4192
411	18-30	Male	NO	Left lane	12	22.565	4216
412	18-30	Male	NO	Right lane	12	22.565	4247
413	18-30	Male	NO	Left lane	2	15.612	4282
414	18-30	Male	NO	Left lane	8	20.105	4295
415	18-30	Male	NO	Left lane	11	22.363	4325
416	18-30	Male	NO	Left lane	10	21.146	4332
417	18-30	Male	NO	Left lane	11	22.363	4350
418	18-30	Male	NO	Left lane	9	20.944	4373
419	18-30	Male	NO	Left lane	8	20.105	4388
420	18-30	Male	NO	Left lane	3	16.45	4404
421	18-30	Male	NO	Left lane	2	15.612	4416
422	18-30	Male	NO	Right lane	8	20.105	4455
423	18-30	Male	NO	Right lane	8	20.105	4463
424	18-30	Male	NO	Right lane	9	20.944	4466
425	18-30	Male	NO	Right lane	9	20.944	4535
426	18-30	Male	NO	Left lane	7	18.685	4540
427	18-30	Male	NO	Right lane	7	18.685	4547
428	18-30	Male	NO	Left lane	11	22.363	4579
429	18-30	Male	NO	Right lane	7	18.685	4579

430	18-30	Male	NO	Right lane	5	17.87	4591
431	18-30	Male	NO	Right lane	6	18.072	4617
432	18-30	Male	NO	Right lane	2	15.612	4624
433	18-30	Male	NO	Left lane	7	18.685	4662
434	18-30	Male	NO	Left lane	7	18.685	4663
435	18-30	Male	NO	Right lane	11	22.363	4692
436	18-30	Male	NO	Left lane	9	20.944	4712
437	18-30	Male	NO	Left lane	10	21.146	4712
438	18-30	Male	NO	Right lane	11	22.363	4713
439	18-30	Male	NO	Right lane	10	21.146	4760
440	18-30	Male	NO	Right lane	12	22.565	4897
441	18-30	Male	NO	Right lane	10	21.146	4981
442	18-30	Female	NO	Right lane	1	14.192	3017
443	18-30	Female	NO	Right lane	2	15.612	3025
444	18-30	Female	NO	Left lane	4	16.652	3080
445	18-30	Female	NO	Left lane	3	16.45	3129
446	18-30	Female	NO	Right lane	3	16.45	3226
447	18-30	Female	NO	Right lane	3	16.45	3301
448	18-30	Female	NO	Right lane	1	14.192	3403
449	18-30	Female	NO	Left lane	1	14.192	3437
450	18-30	Female	NO	Right lane	1	14.192	3455
451	18-30	Female	NO	Right lane	4	16.652	3468
452	18-30	Female	NO	Left lane	2	15.612	3489
453	18-30	Female	NO	Left lane	3	16.45	3505
454	18-30	Female	NO	Right lane	4	16.652	3537
455	18-30	Female	NO	Left lane	1	14.192	3546
456	18-30	Female	NO	Left lane	4	16.652	3563
457	18-30	Female	NO	Left lane	4	16.652	3595
458	18-30	Female	NO	Left lane	5	17.87	3666
459	18-30	Female	NO	Right lane	2	15.612	3667
460	18-30	Female	NO	Left lane	3	16.45	3694
461	18-30	Female	NO	Left lane	1	14.192	3700
462	18-30	Female	NO	Left lane	6	18.072	3720
463	18-30	Female	NO	Right lane	3	16.45	3728
464	18-30	Female	NO	Right lane	7	18.685	3758
465	18-30	Female	NO	Right lane	6	18.072	3765
466	18-30	Female	NO	Left lane	2	15.612	3802
467	18-30	Female	NO	Left lane	2	15.612	3877
468	18-30	Female	NO	Right lane	2	15.612	3891
469	18-30	Female	NO	Right lane	10	21.146	3903
470	18-30	Female	NO	Right lane	6	18.072	3929
471	18-30	Female	NO	Left lane	12	22.565	3959
472	18-30	Female	NO	Left lane	7	18.685	4047
473	18-30	Female	NO	Left lane	6	18.072	4070
474	18-30	Female	NO	Right lane	5	17.87	4154
475	18-30	Female	NO	Right lane	5	17.87	4183
476	18-30	Female	NO	Left lane	6	18.072	4229
477	18-30	Female	NO	Right lane	4	16.652	4260
478	18-30	Female	NO	Left lane	7	18.685	4292
479	18-30	Female	NO	Right lane	8	20.105	4336
480	18-30	Female	NO	Left lane	5	17.87	4338
481	18-30	Female	NO	Left lane	10	21.146	4367
482	18-30	Female	NO	Right lane	10	21.146	4416
483	18-30	Female	NO	Right lane	6	18.072	4417

484	18-30	Female	NO	Right lane	10	21.146	4492
485	18-30	Female	NO	Left lane	12	22.565	4519
486	18-30	Female	NO	Left lane	5	17.87	4547
487	18-30	Female	NO	Left lane	9	20.944	4555
488	18-30	Female	NO	Right lane	5	17.87	4558
489	18-30	Female	NO	Left lane	11	22.363	4564
490	18-30	Female	NO	Left lane	10	21.146	4605
491	18-30	Female	NO	Right lane	9	20.944	4614
492	18-30	Female	NO	Left lane	8	20.105	4619
493	18-30	Female	NO	Left lane	8	20.105	4664
494	18-30	Female	NO	Right lane	9	20.944	4683
495	18-30	Female	NO	Right lane	11	22.363	4687
496	18-30	Female	NO	Right lane	9	20.944	4688
497	18-30	Female	NO	Right lane	8	20.105	4712
498	18-30	Female	NO	Right lane	12	22.565	4729
499	18-30	Female	NO	Right lane	8	20.105	4731
500	18-30	Female	NO	Left lane	9	20.944	4765
501	18-30	Female	NO	Right lane	7	18.685	4792
502	18-30	Female	NO	Left lane	10	21.146	4816
503	18-30	Female	NO	Right lane	11	22.363	4847
504	18-30	Female	NO	Left lane	7	18.685	4862
505	18-30	Female	NO	Right lane	7	18.685	4881
506	18-30	Female	NO	Left lane	9	20.944	4884
507	18-30	Female	NO	Left lane	8	20.105	4919
508	18-30	Female	NO	Right lane	12	22.565	4946
509	18-30	Female	NO	Right lane	12	22.565	4954
510	18-30	Female	NO	Left lane	11	22.363	5136
511	18-30	Female	NO	Left lane	12	22.565	5151
512	18-30	Female	NO	Right lane	11	22.363	5154
513	18-30	Female	NO	Left lane	11	22.363	5212
514	18-30	Female	NO	Left lane	3	16.45	3395
515	18-30	Female	NO	Right lane	9	20.944	3545
516	18-30	Female	NO	Left lane	4	16.652	3752
517	18-30	Female	NO	Right lane	5	17.87	3764
518	18-30	Female	NO	Right lane	8	20.105	3882
519	18-30	Female	NO	Left lane	4	16.652	3885
520	18-30	Female	NO	Left lane	2	15.612	3892
521	18-30	Female	NO	Left lane	3	16.45	3913
522	18-30	Female	NO	Right lane	7	18.685	3959
523	18-30	Female	NO	Left lane	12	22.565	3962
524	18-30	Female	NO	Left lane	1	14.192	3965
525	18-30	Female	NO	Right lane	12	22.565	3999
526	18-30	Female	NO	Right lane	9	20.944	3999
527	18-30	Female	NO	Right lane	1	14.192	4004
528	18-30	Female	NO	Right lane	10	21.146	4012
529	18-30	Female	NO	Left lane	12	22.565	4012
530	18-30	Female	NO	Left lane	6	18.072	4012
531	18-30	Female	NO	Right lane	3	16.45	4020
532	18-30	Female	NO	Left lane	9	20.944	4020
533	18-30	Female	NO	Left lane	7	18.685	4066
534	18-30	Female	NO	Left lane	3	16.45	4073
535	18-30	Female	NO	Left lane	11	22.363	4081
536	18-30	Female	NO	Left lane	1	14.192	4083
537	18-30	Female	NO	Left lane	6	18.072	4101

538	18-30	Female	NO	Right lane	3	16.45	4104
539	18-30	Female	NO	Left lane	1	14.192	4113
540	18-30	Female	NO	Right lane	4	16.652	4149
541	18-30	Female	NO	Left lane	11	22.363	4150
542	18-30	Female	NO	Right lane	5	17.87	4184
543	18-30	Female	NO	Left lane	6	18.072	4184
544	18-30	Female	NO	Left lane	5	17.87	4206
545	18-30	Female	NO	Right lane	5	17.87	4214
546	18-30	Female	NO	Right lane	1	14.192	4215
547	18-30	Female	NO	Right lane	2	15.612	4264
548	18-30	Female	NO	Right lane	10	21.146	4279
549	18-30	Female	NO	Left lane	7	18.685	4284
550	18-30	Female	NO	Right lane	2	15.612	4295
551	18-30	Female	NO	Right lane	8	20.105	4312
552	18-30	Female	NO	Left lane	8	20.105	4329
553	18-30	Female	NO	Left lane	8	20.105	4334
554	18-30	Female	NO	Right lane	11	22.363	4335
555	18-30	Female	NO	Right lane	10	21.146	4371
556	18-30	Female	NO	Right lane	6	18.072	4379
557	18-30	Female	NO	Right lane	4	16.652	4381
558	18-30	Female	NO	Right lane	12	22.565	4386
559	18-30	Female	NO	Left lane	5	17.87	4387
560	18-30	Female	NO	Right lane	2	15.612	4396
561	18-30	Female	NO	Right lane	7	18.685	4403
562	18-30	Female	NO	Left lane	12	22.565	4426
563	18-30	Female	NO	Right lane	8	20.105	4448
564	18-30	Female	NO	Left lane	8	20.105	4461
565	18-30	Female	NO	Left lane	2	15.612	4528
566	18-30	Female	NO	Right lane	7	18.685	4543
567	18-30	Female	NO	Right lane	9	20.944	4547
568	18-30	Female	NO	Left lane	7	18.685	4553
569	18-30	Female	NO	Left lane	11	22.363	4585
570	18-30	Female	NO	Left lane	10	21.146	4606
571	18-30	Female	NO	Right lane	3	16.45	4612
572	18-30	Female	NO	Left lane	2	15.612	4615
573	18-30	Female	NO	Right lane	6	18.072	4634
574	18-30	Female	NO	Right lane	11	22.363	4664
575	18-30	Female	NO	Left lane	9	20.944	4672
576	18-30	Female	NO	Left lane	10	21.146	4697
577	18-30	Female	NO	Right lane	12	22.565	4714
578	18-30	Female	NO	Left lane	5	17.87	4727
579	18-30	Female	NO	Left lane	10	21.146	4788
580	18-30	Female	NO	Left lane	4	16.652	4846
581	18-30	Female	NO	Right lane	6	18.072	4872
582	18-30	Female	NO	Right lane	4	16.652	4935
583	18-30	Female	NO	Right lane	11	22.363	4953
584	18-30	Female	NO	Right lane	1	14.192	4984
585	18-30	Female	NO	Left lane	9	20.944	5002
586	18-30	Female	Yes	Left lane	7	18.685	3854
587	18-30	Female	Yes	Right lane	1	14.192	3907
588	18-30	Female	Yes	Left lane	2	15.612	4130
589	18-30	Female	Yes	Right lane	4	16.652	4436
590	18-30	Female	Yes	Right lane	3	16.45	4458
591	18-30	Female	Yes	Right lane	12	22.565	4555

592	18-30	Female	Yes	Left lane	9	20.944	4604
593	18-30	Female	Yes	Left lane	5	17.87	4614
594	18-30	Female	Yes	Right lane	6	18.072	4654
595	18-30	Female	Yes	Left lane	10	21.146	4739
596	18-30	Female	Yes	Right lane	11	22.363	4863
597	18-30	Female	Yes	Left lane	8	20.105	4934
598	18-30	Female	Yes	Right lane	7	18.685	3157
599	18-30	Female	Yes	Right lane	3	16.45	3418
600	18-30	Female	Yes	Left lane	9	20.944	3458
601	18-30	Female	Yes	Right lane	8	20.105	3465
602	18-30	Female	Yes	Right lane	3	16.45	3484
603	18-30	Female	Yes	Left lane	4	16.652	3533
604	18-30	Female	Yes	Left lane	7	18.685	3557
605	18-30	Female	Yes	Right lane	1	14.192	3601
606	18-30	Female	Yes	Right lane	4	16.652	3620
607	18-30	Female	Yes	Left lane	12	22.565	3652
608	18-30	Female	Yes	Right lane	12	22.565	3665
609	18-30	Female	Yes	Right lane	7	18.685	3671
610	18-30	Female	Yes	Right lane	3	16.45	3671
611	18-30	Female	Yes	Left lane	1	14.192	3674
612	18-30	Female	Yes	Right lane	6	18.072	3684
613	18-30	Female	Yes	Left lane	12	22.565	3814
614	18-30	Female	Yes	Right lane	6	18.072	3827
615	18-30	Female	Yes	Right lane	2	15.612	3833
616	18-30	Female	Yes	Left lane	2	15.612	3838
617	18-30	Female	Yes	Left lane	4	16.652	3848
618	18-30	Female	Yes	Left lane	9	20.944	3857
619	18-30	Female	Yes	Left lane	3	16.45	3881
620	18-30	Female	Yes	Left lane	4	16.652	3885
621	18-30	Female	Yes	Right lane	10	21.146	3899
622	18-30	Female	Yes	Right lane	10	21.146	3901
623	18-30	Female	Yes	Left lane	5	17.87	3905
624	18-30	Female	Yes	Right lane	7	18.685	3913
625	18-30	Female	Yes	Left lane	3	16.45	3916
626	18-30	Female	Yes	Left lane	5	17.87	3917
627	18-30	Female	Yes	Left lane	1	14.192	3919
628	18-30	Female	Yes	Right lane	11	22.363	3948
629	18-30	Female	Yes	Left lane	1	14.192	3953
630	18-30	Female	Yes	Right lane	12	22.565	3962
631	18-30	Female	Yes	Right lane	9	20.944	3972
632	18-30	Female	Yes	Right lane	11	22.363	3982
633	18-30	Female	Yes	Left lane	10	21.146	3982
634	18-30	Female	Yes	Right lane	8	20.105	3985
635	18-30	Female	Yes	Right lane	6	18.072	3999
636	18-30	Female	Yes	Left lane	7	18.685	4002
637	18-30	Female	Yes	Left lane	3	16.45	4013
638	18-30	Female	Yes	Right lane	9	20.944	4013
639	18-30	Female	Yes	Right lane	5	17.87	4026
640	18-30	Female	Yes	Right lane	2	15.612	4026
641	18-30	Female	Yes	Left lane	11	22.363	4033
642	18-30	Female	Yes	Left lane	10	21.146	4048
643	18-30	Female	Yes	Right lane	1	14.192	4064
644	18-30	Female	Yes	Left lane	6	18.072	4069
645	18-30	Female	Yes	Right lane	10	21.146	4079

646	18-30	Female	Yes	Right lane	12	22.565	4079
647	18-30	Female	Yes	Right lane	11	22.363	4100
648	18-30	Female	Yes	Left lane	5	17.87	4112
649	18-30	Female	Yes	Right lane	5	17.87	4125
650	18-30	Female	Yes	Right lane	5	17.87	4150
651	18-30	Female	Yes	Left lane	2	15.612	4154
652	18-30	Female	Yes	Right lane	9	20.944	4160
653	18-30	Female	Yes	Right lane	4	16.652	4206
654	18-30	Female	Yes	Right lane	1	14.192	4223
655	18-30	Female	Yes	Left lane	8	20.105	4226
656	18-30	Female	Yes	Left lane	6	18.072	4239
657	18-30	Female	Yes	Right lane	8	20.105	4248
658	18-30	Female	Yes	Left lane	11	22.363	4271
659	18-30	Female	Yes	Left lane	8	20.105	4304
660	18-30	Female	Yes	Right lane	4	16.652	4334
661	18-30	Female	Yes	Right lane	2	15.612	4343
662	18-30	Female	Yes	Left lane	6	18.072	4373
663	18-30	Female	Yes	Left lane	8	20.105	4415
664	18-30	Female	Yes	Left lane	11	22.363	4426
665	18-30	Female	Yes	Left lane	10	21.146	4459
666	18-30	Female	Yes	Left lane	2	15.612	4524
667	18-30	Female	Yes	Left lane	9	20.944	4533
668	18-30	Female	Yes	Left lane	12	22.565	4551
669	18-30	Female	Yes	Left lane	7	18.685	4725
670	18-30	Female	NO	Right lane	1	14.192	3813
671	18-30	Female	NO	Right lane	6	18.072	4261
672	18-30	Female	NO	Right lane	3	16.45	4343
673	18-30	Female	NO	Left lane	2	15.612	4439
674	18-30	Female	NO	Right lane	4	16.652	4491
675	18-30	Female	NO	Left lane	8	20.105	4693
676	18-30	Female	NO	Right lane	11	22.363	4819
677	18-30	Female	NO	Left lane	7	18.685	4901
678	18-30	Female	NO	Right lane	12	22.565	4916
679	18-30	Female	NO	Left lane	5	17.87	4919
680	18-30	Female	NO	Left lane	10	21.146	4958
681	18-30	Female	NO	Left lane	9	20.944	5013
682	18-30	Male	NO	Left lane	2	15.612	3463
683	18-30	Male	NO	Right lane	3	16.45	3645
684	18-30	Male	NO	Right lane	1	14.192	3869
685	18-30	Male	NO	Right lane	6	18.072	3898
686	18-30	Male	NO	Left lane	9	20.944	4082
687	18-30	Male	NO	Right lane	4	16.652	4312
688	18-30	Male	NO	Left lane	5	17.87	4857
689	18-30	Male	NO	Left lane	8	20.105	5013
690	18-30	Male	NO	Left lane	10	21.146	5087
691	18-30	Male	NO	Right lane	11	22.363	5179
692	18-30	Male	NO	Left lane	7	18.685	5180
693	18-30	Male	NO	Right lane	12	22.565	5324
694	18-30	Male	Yes	Right lane	8	20.105	3216
695	18-30	Male	Yes	Left lane	4	16.652	3335
696	18-30	Male	Yes	Right lane	10	21.146	3338
697	18-30	Male	Yes	Right lane	9	20.944	3365
698	18-30	Male	Yes	Left lane	3	16.45	3393
699	18-30	Male	Yes	Left lane	3	16.45	3398

700	18-30	Male	Yes	Right lane	2	15.612	3489
701	18-30	Male	Yes	Left lane	3	16.45	3586
702	18-30	Male	Yes	Left lane	4	16.652	3587
703	18-30	Male	Yes	Left lane	1	14.192	3624
704	18-30	Male	Yes	Right lane	10	21.146	3625
705	18-30	Male	Yes	Left lane	4	16.652	3647
706	18-30	Male	Yes	Left lane	11	22.363	3717
707	18-30	Male	Yes	Right lane	6	18.072	3721
708	18-30	Male	Yes	Right lane	5	17.87	3726
709	18-30	Male	Yes	Left lane	1	14.192	3732
710	18-30	Male	Yes	Right lane	4	16.652	3780
711	18-30	Male	Yes	Right lane	9	20.944	3782
712	18-30	Male	Yes	Right lane	4	16.652	3796
713	18-30	Male	Yes	Right lane	1	14.192	3819
714	18-30	Male	Yes	Right lane	9	20.944	3852
715	18-30	Male	Yes	Right lane	5	17.87	3939
716	18-30	Male	Yes	Right lane	3	16.45	3940
717	18-30	Male	Yes	Right lane	12	22.565	4001
718	18-30	Male	Yes	Left lane	11	22.363	4024
719	18-30	Male	Yes	Right lane	2	15.612	4035
720	18-30	Male	Yes	Left lane	12	22.565	4053
721	18-30	Male	Yes	Left lane	2	15.612	4061
722	18-30	Male	Yes	Right lane	6	18.072	4079
723	18-30	Male	Yes	Left lane	6	18.072	4084
724	18-30	Male	Yes	Left lane	10	21.146	4149
725	18-30	Male	Yes	Right lane	6	18.072	4151
726	18-30	Male	Yes	Left lane	7	18.685	4166
727	18-30	Male	Yes	Right lane	3	16.45	4173
728	18-30	Male	Yes	Right lane	11	22.363	4216
729	18-30	Male	Yes	Right lane	8	20.105	4228
730	18-30	Male	Yes	Left lane	5	17.87	4258
731	18-30	Male	Yes	Right lane	4	16.652	4261
732	18-30	Male	Yes	Left lane	12	22.565	4262
733	18-30	Male	Yes	Left lane	1	14.192	4272
734	18-30	Male	Yes	Left lane	10	21.146	4283
735	18-30	Male	Yes	Left lane	6	18.072	4287
736	18-30	Male	Yes	Left lane	8	20.105	4294
737	18-30	Male	Yes	Left lane	2	15.612	4314
738	18-30	Male	Yes	Right lane	11	22.363	4333
739	18-30	Male	Yes	Left lane	2	15.612	4358
740	18-30	Male	Yes	Right lane	12	22.565	4359
741	18-30	Male	Yes	Right lane	10	21.146	4370
742	18-30	Male	Yes	Right lane	2	15.612	4380
743	18-30	Male	Yes	Right lane	12	22.565	4391
744	18-30	Male	Yes	Left lane	6	18.072	4394
745	18-30	Male	Yes	Left lane	5	17.87	4401
746	18-30	Male	Yes	Right lane	1	14.192	4435
747	18-30	Male	Yes	Left lane	9	20.944	4450
748	18-30	Male	Yes	Right lane	7	18.685	4451
749	18-30	Male	Yes	Left lane	12	22.565	4468
750	18-30	Male	Yes	Left lane	8	20.105	4494
751	18-30	Male	Yes	Left lane	5	17.87	4529
752	18-30	Male	Yes	Left lane	9	20.944	4533
753	18-30	Male	Yes	Left lane	7	18.685	4538

754	18-30	Male	Yes	Right lane	11	22.363	4580
755	18-30	Male	Yes	Left lane	10	21.146	4585
756	18-30	Male	Yes	Left lane	11	22.363	4587
757	18-30	Male	Yes	Left lane	8	20.105	4614
758	18-30	Male	Yes	Right lane	7	18.685	4622
759	18-30	Male	Yes	Left lane	7	18.685	4630
760	18-30	Male	Yes	Right lane	1	14.192	4646
761	18-30	Male	Yes	Right lane	8	20.105	4652
762	18-30	Male	Yes	Right lane	3	16.45	4758
763	18-30	Male	Yes	Left lane	9	20.944	4773
764	18-30	Male	Yes	Right lane	5	17.87	4960
765	18-30	Male	Yes	Right lane	7	18.685	5115
766	18-30	Male	NO	Right lane	2	15.612	3094
767	18-30	Male	NO	Left lane	12	22.565	3422
768	18-30	Male	NO	Right lane	10	21.146	3547
769	18-30	Male	NO	Right lane	9	20.944	3626
770	18-30	Male	NO	Left lane	1	14.192	3683
771	18-30	Male	NO	Right lane	8	20.105	3693
772	18-30	Male	NO	Left lane	4	16.652	3736
773	18-30	Male	NO	Right lane	3	16.45	3847
774	18-30	Male	NO	Left lane	11	22.363	3863
775	18-30	Male	NO	Left lane	1	14.192	3865
776	18-30	Male	NO	Left lane	3	16.45	3924
777	18-30	Male	NO	Right lane	1	14.192	3963
778	18-30	Male	NO	Left lane	6	18.072	3983
779	18-30	Male	NO	Right lane	7	18.685	3992
780	18-30	Male	NO	Right lane	6	18.072	4031
781	18-30	Male	NO	Left lane	5	17.87	4047
782	18-30	Male	NO	Left lane	12	22.565	4081
783	18-30	Male	NO	Right lane	12	22.565	4119
784	18-30	Male	NO	Right lane	4	16.652	4154
785	18-30	Male	NO	Right lane	1	14.192	4193
786	18-30	Male	NO	Left lane	10	21.146	4201
787	18-30	Male	NO	Right lane	5	17.87	4218
788	18-30	Male	NO	Left lane	7	18.685	4223
789	18-30	Male	NO	Right lane	2	15.612	4233
790	18-30	Male	NO	Left lane	6	18.072	4240
791	18-30	Male	NO	Left lane	2	15.612	4249
792	18-30	Male	NO	Left lane	10	21.146	4254
793	18-30	Male	NO	Right lane	1	14.192	4255
794	18-30	Male	NO	Right lane	5	17.87	4284
795	18-30	Male	NO	Right lane	6	18.072	4284
796	18-30	Male	NO	Right lane	4	16.652	4292
797	18-30	Male	NO	Right lane	2	15.612	4303
798	18-30	Male	NO	Left lane	7	18.685	4316
799	18-30	Male	NO	Left lane	8	20.105	4322
800	18-30	Male	NO	Left lane	8	20.105	4339
801	18-30	Male	NO	Left lane	1	14.192	4360
802	18-30	Male	NO	Left lane	5	17.87	4364
803	18-30	Male	NO	Right lane	8	20.105	4367
804	18-30	Male	NO	Left lane	12	22.565	4367
805	18-30	Male	NO	Left lane	9	20.944	4380
806	18-30	Male	NO	Right lane	11	22.363	4414
807	18-30	Male	NO	Right lane	5	17.87	4415

808	18-30	Male	NO	Left lane	4	16.652	4417
809	18-30	Male	NO	Right lane	12	22.565	4429
810	18-30	Male	NO	Right lane	10	21.146	4448
811	18-30	Male	NO	Left lane	2	15.612	4467
812	18-30	Male	NO	Right lane	11	22.363	4468
813	18-30	Male	NO	Left lane	3	16.45	4471
814	18-30	Male	NO	Right lane	3	16.45	4481
815	18-30	Male	NO	Right lane	7	18.685	4481
816	18-30	Male	NO	Left lane	7	18.685	4482
817	18-30	Male	NO	Left lane	4	16.652	4492
818	18-30	Male	NO	Left lane	9	20.944	4513
819	18-30	Male	NO	Left lane	6	18.072	4532
820	18-30	Male	NO	Right lane	3	16.45	4538
821	18-30	Male	NO	Left lane	2	15.612	4550
822	18-30	Male	NO	Right lane	6	18.072	4574
823	18-30	Male	NO	Right lane	7	18.685	4583
824	18-30	Male	NO	Left lane	3	16.45	4586
825	18-30	Male	NO	Left lane	5	17.87	4589
826	18-30	Male	NO	Right lane	12	22.565	4595
827	18-30	Male	NO	Left lane	9	20.944	4614
828	18-30	Male	NO	Left lane	11	22.363	4617
829	18-30	Male	NO	Right lane	4	16.652	4630
830	18-30	Male	NO	Right lane	9	20.944	4630
831	18-30	Male	NO	Right lane	9	20.944	4635
832	18-30	Male	NO	Right lane	10	21.146	4681
833	18-30	Male	NO	Right lane	8	20.105	4701
834	18-30	Male	NO	Right lane	11	22.363	4777
835	18-30	Male	NO	Left lane	8	20.105	4852
836	18-30	Male	NO	Left lane	11	22.363	4870
837	18-30	Male	NO	Left lane	10	21.146	4980
838	18-30	Female	NO	Left lane	1	14.192	2797
839	18-30	Female	NO	Right lane	1	14.192	3034
840	18-30	Female	NO	Right lane	1	14.192	3321
841	18-30	Female	NO	Right lane	3	16.45	3350
842	18-30	Female	NO	Left lane	6	18.072	3370
843	18-30	Female	NO	Left lane	2	15.612	3487
844	18-30	Female	NO	Left lane	3	16.45	3515
845	18-30	Female	NO	Right lane	1	14.192	3549
846	18-30	Female	NO	Left lane	3	16.45	3588
847	18-30	Female	NO	Left lane	12	22.565	3648
848	18-30	Female	NO	Right lane	4	16.652	3681
849	18-30	Female	NO	Left lane	8	20.105	3730
850	18-30	Female	NO	Left lane	6	18.072	3828
851	18-30	Female	NO	Right lane	6	18.072	3854
852	18-30	Female	NO	Left lane	3	16.45	3859
853	18-30	Female	NO	Left lane	4	16.652	3859
854	18-30	Female	NO	Left lane	2	15.612	3886
855	18-30	Female	NO	Left lane	4	16.652	3899
856	18-30	Female	NO	Right lane	2	15.612	3914
857	18-30	Female	NO	Right lane	3	16.45	3928
858	18-30	Female	NO	Right lane	3	16.45	4014
859	18-30	Female	NO	Left lane	7	18.685	4031
860	18-30	Female	NO	Right lane	4	16.652	4047
861	18-30	Female	NO	Left lane	5	17.87	4048

862	18-30	Female	NO	Left lane	1	14.192	4089
863	18-30	Female	NO	Left lane	8	20.105	4095
864	18-30	Female	NO	Right lane	10	21.146	4126
865	18-30	Female	NO	Right lane	6	18.072	4126
866	18-30	Female	NO	Left lane	10	21.146	4134
867	18-30	Female	NO	Left lane	1	14.192	4140
868	18-30	Female	NO	Right lane	4	16.652	4146
869	18-30	Female	NO	Right lane	5	17.87	4152
870	18-30	Female	NO	Right lane	2	15.612	4203
871	18-30	Female	NO	Left lane	12	22.565	4216
872	18-30	Female	NO	Left lane	5	17.87	4219
873	18-30	Female	NO	Left lane	4	16.652	4303
874	18-30	Female	NO	Right lane	9	20.944	4328
875	18-30	Female	NO	Left lane	12	22.565	4379
876	18-30	Female	NO	Left lane	5	17.87	4387
877	18-30	Female	NO	Left lane	11	22.363	4405
878	18-30	Female	NO	Right lane	5	17.87	4422
879	18-30	Female	NO	Right lane	2	15.612	4426
880	18-30	Female	NO	Right lane	12	22.565	4435
881	18-30	Female	NO	Right lane	8	20.105	4449
882	18-30	Female	NO	Left lane	7	18.685	4451
883	18-30	Female	NO	Right lane	7	18.685	4462
884	18-30	Female	NO	Right lane	7	18.685	4512
885	18-30	Female	NO	Right lane	11	22.363	4529
886	18-30	Female	NO	Right lane	11	22.363	4546
887	18-30	Female	NO	Right lane	12	22.565	4551
888	18-30	Female	NO	Left lane	10	21.146	4569
889	18-30	Female	NO	Right lane	9	20.944	4596
890	18-30	Female	NO	Right lane	8	20.105	4609
891	18-30	Female	NO	Left lane	2	15.612	4650
892	18-30	Female	NO	Left lane	10	21.146	4703
893	18-30	Female	NO	Left lane	6	18.072	4713
894	18-30	Female	NO	Right lane	12	22.565	4747
895	18-30	Female	NO	Left lane	11	22.363	4770
896	18-30	Female	NO	Right lane	5	17.87	4815
897	18-30	Female	NO	Left lane	11	22.363	4839
898	18-30	Female	NO	Left lane	9	20.944	4898
899	18-30	Female	NO	Right lane	10	21.146	4902
900	18-30	Female	NO	Right lane	6	18.072	4906
901	18-30	Female	NO	Left lane	8	20.105	4938
902	18-30	Female	NO	Right lane	9	20.944	5026
903	18-30	Female	NO	Left lane	9	20.944	5034
904	18-30	Female	NO	Right lane	7	18.685	5115
905	18-30	Female	NO	Left lane	7	18.685	5122
906	18-30	Female	NO	Right lane	11	22.363	5148
907	18-30	Female	NO	Right lane	8	20.105	5152
908	18-30	Female	NO	Left lane	9	20.944	5214
909	18-30	Male	NO	Left lane	12	22.565	3262
910	18-30	Male	NO	Right lane	1	14.192	3685
911	18-30	Male	NO	Left lane	4	16.652	3687
912	18-30	Male	NO	Right lane	2	15.612	3717
913	18-30	Male	NO	Right lane	9	20.944	3772
914	18-30	Male	NO	Right lane	3	16.45	3813
915	18-30	Male	NO	Left lane	1	14.192	3951

916	18-30	Male	NO	Right lane	5	17.87	3955
917	18-30	Male	NO	Left lane	3	16.45	3962
918	18-30	Male	NO	Left lane	1	14.192	3965
919	18-30	Male	NO	Right lane	10	21.146	3998
920	18-30	Male	NO	Left lane	4	16.652	4030
921	18-30	Male	NO	Left lane	6	18.072	4045
922	18-30	Male	NO	Right lane	4	16.652	4079
923	18-30	Male	NO	Right lane	8	20.105	4130
924	18-30	Male	NO	Left lane	5	17.87	4149
925	18-30	Male	NO	Left lane	8	20.105	4156
926	18-30	Male	NO	Right lane	10	21.146	4164
927	18-30	Male	NO	Left lane	11	22.363	4181
928	18-30	Male	NO	Right lane	4	16.652	4182
929	18-30	Male	NO	Right lane	12	22.565	4188
930	18-30	Male	NO	Left lane	4	16.652	4193
931	18-30	Male	NO	Left lane	11	22.363	4193
932	18-30	Male	NO	Right lane	7	18.685	4226
933	18-30	Male	NO	Left lane	2	15.612	4248
934	18-30	Male	NO	Right lane	7	18.685	4249
935	18-30	Male	NO	Left lane	1	14.192	4250
936	18-30	Male	NO	Left lane	10	21.146	4288
937	18-30	Male	NO	Right lane	11	22.363	4357
938	18-30	Male	NO	Right lane	3	16.45	4366
939	18-30	Male	NO	Right lane	2	15.612	4366
940	18-30	Male	NO	Right lane	5	17.87	4380
941	18-30	Male	NO	Left lane	8	20.105	4380
942	18-30	Male	NO	Left lane	5	17.87	4380
943	18-30	Male	NO	Left lane	3	16.45	4426
944	18-30	Male	NO	Left lane	5	17.87	4427
945	18-30	Male	NO	Right lane	4	16.652	4440
946	18-30	Male	NO	Left lane	3	16.45	4451
947	18-30	Male	NO	Left lane	2	15.612	4455
948	18-30	Male	NO	Left lane	10	21.146	4459
949	18-30	Male	NO	Left lane	12	22.565	4459
950	18-30	Male	NO	Left lane	7	18.685	4465
951	18-30	Male	NO	Right lane	1	14.192	4467
952	18-30	Male	NO	Right lane	10	21.146	4472
953	18-30	Male	NO	Left lane	10	21.146	4479
954	18-30	Male	NO	Right lane	9	20.944	4492
955	18-30	Male	NO	Right lane	5	17.87	4498
956	18-30	Male	NO	Left lane	9	20.944	4499
957	18-30	Male	NO	Left lane	8	20.105	4514
958	18-30	Male	NO	Left lane	6	18.072	4517
959	18-30	Male	NO	Left lane	2	15.612	4520
960	18-30	Male	NO	Right lane	6	18.072	4567
961	18-30	Male	NO	Right lane	6	18.072	4581
962	18-30	Male	NO	Right lane	3	16.45	4586
963	18-30	Male	NO	Right lane	11	22.363	4606
964	18-30	Male	NO	Left lane	7	18.685	4624
965	18-30	Male	NO	Right lane	6	18.072	4648
966	18-30	Male	NO	Left lane	9	20.944	4660
967	18-30	Male	NO	Left lane	9	20.944	4684
968	18-30	Male	NO	Left lane	12	22.565	4712
969	18-30	Male	NO	Right lane	11	22.363	4740

970	18-30	Male	NO	Right lane	12	22.565	4748
971	18-30	Male	NO	Right lane	2	15.612	4821
972	18-30	Male	NO	Right lane	8	20.105	4831
973	18-30	Male	NO	Left lane	7	18.685	4839
974	18-30	Male	NO	Left lane	11	22.363	4861
975	18-30	Male	NO	Right lane	1	14.192	4903
976	18-30	Male	NO	Left lane	6	18.072	4923
977	18-30	Male	NO	Right lane	9	20.944	4968
978	18-30	Male	NO	Right lane	12	22.565	4998
979	18-30	Male	NO	Right lane	7	18.685	5101
980	18-30	Male	NO	Right lane	8	20.105	5126
981	18-30	Male	Yes	Left lane	10	21.146	2387
982	18-30	Male	Yes	Left lane	3	16.45	2784
983	18-30	Male	Yes	Right lane	4	16.652	2847
984	18-30	Male	Yes	Left lane	4	16.652	2859
985	18-30	Male	Yes	Left lane	8	20.105	3162
986	18-30	Male	Yes	Right lane	1	14.192	3191
987	18-30	Male	Yes	Right lane	4	16.652	3202
988	18-30	Male	Yes	Left lane	4	16.652	3248
989	18-30	Male	Yes	Right lane	3	16.45	3252
990	18-30	Male	Yes	Right lane	8	20.105	3273
991	18-30	Male	Yes	Left lane	2	15.612	3312
992	18-30	Male	Yes	Left lane	6	18.072	3397
993	18-30	Male	Yes	Right lane	8	20.105	3410
994	18-30	Male	Yes	Left lane	5	17.87	3433
995	18-30	Male	Yes	Left lane	12	22.565	3434
996	18-30	Male	Yes	Right lane	4	16.652	3447
997	18-30	Male	Yes	Left lane	2	15.612	3460
998	18-30	Male	Yes	Right lane	3	16.45	3461
999	18-30	Male	Yes	Right lane	12	22.565	3482
1000	18-30	Male	Yes	Right lane	7	18.685	3502
1001	18-30	Male	Yes	Right lane	5	17.87	3515
1002	18-30	Male	Yes	Left lane	4	16.652	3515
1003	18-30	Male	Yes	Right lane	12	22.565	3516
1004	18-30	Male	Yes	Left lane	7	18.685	3535
1005	18-30	Male	Yes	Right lane	2	15.612	3535
1006	18-30	Male	Yes	Left lane	5	17.87	3536
1007	18-30	Male	Yes	Right lane	9	20.944	3536
1008	18-30	Male	Yes	Right lane	6	18.072	3580
1009	18-30	Male	Yes	Right lane	12	22.565	3591
1010	18-30	Male	Yes	Left lane	11	22.363	3596
1011	18-30	Male	Yes	Right lane	8	20.105	3600
1012	18-30	Male	Yes	Right lane	5	17.87	3622
1013	18-30	Male	Yes	Left lane	7	18.685	3627
1014	18-30	Male	Yes	Right lane	7	18.685	3639
1015	18-30	Male	Yes	Right lane	9	20.944	3668
1016	18-30	Male	Yes	Left lane	3	16.45	3672
1017	18-30	Male	Yes	Right lane	3	16.45	3683
1018	18-30	Male	Yes	Right lane	1	14.192	3717
1019	18-30	Male	Yes	Left lane	9	20.944	3735
1020	18-30	Male	Yes	Right lane	10	21.146	3751
1021	18-30	Male	Yes	Right lane	6	18.072	3767
1022	18-30	Male	Yes	Right lane	1	14.192	3783
1023	18-30	Male	Yes	Left lane	1	14.192	3799

1024	18-30	Male	Yes	Left lane	2	15.612	3810
1025	18-30	Male	Yes	Left lane	11	22.363	3813
1026	18-30	Male	Yes	Left lane	9	20.944	3813
1027	18-30	Male	Yes	Right lane	2	15.612	3827
1028	18-30	Male	Yes	Left lane	7	18.685	3848
1029	18-30	Male	Yes	Right lane	10	21.146	3858
1030	18-30	Male	Yes	Right lane	6	18.072	3871
1031	18-30	Male	Yes	Right lane	5	17.87	3872
1032	18-30	Male	Yes	Right lane	2	15.612	3948
1033	18-30	Male	Yes	Right lane	11	22.363	3961
1034	18-30	Male	Yes	Right lane	10	21.146	3990
1035	18-30	Male	Yes	Left lane	6	18.072	3994
1036	18-30	Male	Yes	Right lane	11	22.363	4046
1037	18-30	Male	Yes	Left lane	10	21.146	4048
1038	18-30	Male	Yes	Left lane	5	17.87	4059
1039	18-30	Male	Yes	Left lane	8	20.105	4112
1040	18-30	Male	Yes	Left lane	1	14.192	4119
1041	18-30	Male	Yes	Left lane	11	22.363	4154
1042	18-30	Male	Yes	Right lane	9	20.944	4184
1043	18-30	Male	Yes	Left lane	10	21.146	4217
1044	18-30	Male	Yes	Left lane	9	20.944	4220
1045	18-30	Male	Yes	Left lane	1	14.192	4222
1046	18-30	Male	Yes	Left lane	6	18.072	4251
1047	18-30	Male	Yes	Left lane	3	16.45	4295
1048	18-30	Male	Yes	Left lane	12	22.565	4303
1049	18-30	Male	Yes	Right lane	7	18.685	4329
1050	18-30	Male	Yes	Right lane	11	22.363	4448
1051	18-30	Male	Yes	Left lane	12	22.565	4571
