# Supplementary material for: Setting method of exit advance guide signs in mountainous expressway tunnel based on information quantization theory
Source: PLoS One. 2023 Feb 16;18(2):e0281842. doi: 10.1371/journal.pone.0281842 (PMC9934451; doi:10.1371/journal.pone.0281842)
Supplement: S3 Table — (PDF) [file pone.0281842.s003.pdf]

Reaction time of Exit advance guide signs of two-character place names

Combination1	2	3	4	5	6	7	8	9	10	11	Combination 12
3547	3251	3036	3457	3230	3849	/	3728	3729	3494	/	4017
3940	3698	3464	3738	3446	3919	3673	3795	3838	3577	/	4096
3161	3528	2716	3017	3473	3760	3738	3407	3759	3056	4192	3877
3639	3613	3579	3427	3914	4017	3831	4088	3320	3916	4072	3283
3326	3728	3464	3368	3720	3873	3882	3833	3588	4259	3754	4120
3987	3696	3817	3592	3646	4012	3884	3716	3851	4005	3964	3894
3780	3581	3152	2796	3680	3698	3902	/	4248	4126	4113	3468
3059	3558	3038	3304	4018	4314	3931	4239	3915	4548	4139	4426
3837	3717	4291	3881	4014	4113	3938	4216	3785	4413	3881	4555
3854	4064	3391	3951	3843	4450	3969	4316	4186	4739	4752	4604
3305	3597	3106	3183	3736	3912	3984	4277	4194	3614	4660	4273
3865	3869	3482	2958	4000	4061	3995	3793	4417	4428	4514	4024
3959	3914	3482	3650	4001	4350	4003	3871	4492	4584	4720	4229
3453	3577	3063	3617	4132	4960	4012	4346	3948	4903	4281	4779
3717	3591	3582	3525	3695	3829	4029	3821	3871	3347	4181	3929
/	3717	3634	3753	3446	3927	4033	3864	3847	3658	/	/
4103	4006	4376	3983	4158	4344	4058	3948	4531	4338	3610	4451
3727	3647	3659	3612	3792	3988	4072	4003	4125	3601	4246	3950
4153	3777	3859	3986	3692	4181	4081	4138	3948	4147	4246	4613
/	3752	3672	/	3579	/	4101	4070	3992	/	/	/
4196	4081	3966	4053	4026	4625	4122	4514	4219	4315	4559	4683
3632	4417	4418	4167	4063	3625	4124	4567	4466	3517	4693	/
4051	3779	3862	3697	3725	4182	4125	4034	4017	4248	3973	4079
3812	3829	3692	3895	3806	4055	4131	3791	3948	3751	4247	3839
4072	3940	3924	3845	3831	4434	4138	4059	4063	4306	4246	4135
3864	3983	3954	4079	3984	4288	4146	4225	4161	4525	4551	4517
4020	3750	4315	4260	4051	4280	4187	4258	4126	4522	4185	4595
4138	3984	4499	4281	4250	4966	4208	4391	4429	4681	4218	4814
3931	4098	3704	3519	4050	4558	4214	4595	4049	5102	4652	4591
4181	4135	4504	4044	4203	4372	4226	3988	4806	4391	3684	4591
/	3866	3885	/	3595	/	4230	/	4092	/	/	/
3967	4014	4184	4182	4105	4479	4231	4227	4186	4802	4568	4776
4025	4158	3501	4144	4054	4562	4247	4735	4459	4870	4898	4658
4569	3781	3295	3926	4271	4997	4249	4380	4246	5327	4386	4999
/	4401	4289	3891	4250	4616	4255	4816	4647	5113	4854	4758
3791	3860	3714	3726	4184	4055	4257	4070	4255	3725	4449	4112
3397	3580	3416	3672	3518	4060	4258	4288	4014	4426	4192	4216
3434	3653	3504	3680	3539	4251	4285	4386	4371	4458	4258	4319
4638	3869	3773	3780	4336	4121	4292	4396	4181	4499	4433	4246
/	3928	3516	3795	4015	4416	4313	4057	4552	4705	4731	4358
3848	3740	3585	4046	3987	4114	4314	4222	3922	3929	4220	3924
3661	3758	3532	3560	3839	4216	4314	4133	3726	4402	3938	4331
2913	4124	4271	4834	4868	3871	4316	4826	4887	3545	4788	5066
3538	4394	4091	3906	4217	4834	4336	5055	4517	4670	4847	5264
3813	3925	4032	3729	4226	4226	4351	4155	4780	3783	4694	4287
4427	4204	3904	4146	4353	4731	4351	4777	4781	4981	5040	5034
3557	3935	4660	4497	4391	3960	4355	4692	4859	3871	3929	4346
/	4296	4250	4099	4524	4349	4358	4417	4200	4504	4610	4397
3632	3907	3157	3412	4000	4024	4358	4393	4448	3650	4681	4424
3736	3950	3814	3682	3680	4324	4369	4403	4403	4663	4449	4429
2740	4727	4382	4193	4577	4884	4372	4419	4359	4529	4952	4503

3517	4173	3495	3880	4246	4684	4377	4719	4564	4735	4893	4578
3762	4884	5058	4313	4599	3749	4386	5182	4510	5094	4979	4882
4266	3892	4145	4064	4125	4124	4397	4635	3954	4196	4513	3928
3715	4401	3658	4253	5026	4324	4400	4744	5147	4992	4709	4836
3602	3952	4002	3879	3800	4369	4419	4547	4548	4920	4492	4579
3753	3780	3665	3693	4008	4269	4435	4392	3771	4683	4361	4514
/	3925	/	/	3647	/	4437	/	/	/	/	/
3126	4438	4388	3972	4606	4778	4453	4888	4824	5113	5146	5194
3847	3986	3784	3965	3960	4087	4460	4104	4332	4361	4406	4038
4302	4215	4581	4225	4507	4455	4465	4116	4818	4518	4091	4602
4214	4419	3181	3516	4102	4895	4477	4940	5198	4354	5046	4902
2792	4158	4538	4387	4433	3866	4479	4481	4529	4097	4479	4071
3961	4153	3854	4147	4204	4339	4479	4262	4403	4535	4502	4125
3494	4197	3773	4613	4352	4305	4496	5100	4947	5312	4815	4258
2865	4339	4036	4082	4547	3679	4500	4313	4650	5192	5126	4753
3725	4419	4430	4259	4092	3858	4561	4921	4500	4203	4700	4215
/	3887	3780	3773	4292	4592	4561	4440	4217	4708	4765	4529
4010	4236	3857	4185	4258	4544	4577	4364	4606	4716	4510	4258
3273	4235	3116	4193	4039	4830	4584	4558	5046	5284	4591	4714
3684	3999	4013	4030	4148	4037	4602	4692	4782	4649	4347	4648
2960	4221	4647	4421	4451	4040	4606	4517	5022	4389	4648	4227
4660	4246	4272	4198	4439	4699	4606	4510	4649	4827	4570	4494
/	3939	4350	4417	4185	4333	4616	4669	4282	4329	4648	4394
3404	4396	3592	4515	4591	3895	4621	4600	5002	3582	3626	3739
3248	4252	4681	4628	4535	3488	4648	4893	4871	2921	4095	3352
3761	4555	4031	3035	4745	4034	4650	3339	3685	4349	4719	4361
3396	4461	4475	4014	4684	4136	4650	4900	5072	4672	4166	5236
3881	4035	4225	3614	4067	4069	4660	4479	4772	4182	4713	4464
4306	4371	3861	3565	3894	4743	4663	4863	4812	4506	5019	4819
/	4232	4299	4167	4394	5027	4667	4800	5145	5192	5125	5138
3365	4598	3824	3891	4259	4704	4673	4624	4705	5014	5160	5202
3232	4389	3514	4339	4654	4662	4691	5255	4798	4914	3962	5103
3259	4467	4881	4802	4632	3591	4693	4931	5184	3080	4171	3547
4183	4207	3648	4003	4255	4796	4693	4735	4652	4750	4950	4914
3049	4462	4452	4394	4806	3828	4704	4758	4800	4018	3736	5168
3907	4164	4374	3787	4195	4694	4705	4612	4783	4467	4805	4493
3059	4291	2794	3155	4473	4215	4733	3897	3399	4520	4740	4244
3548	4471	3351	4813	4712	4348	4762	5149	5314	4682	4495	4528
3260	3914	3926	3964	4535	3781	4766	4610	4359	5125	5068	4598
3681	4515	3819	4422	4825	4185	4772	5081	4573	5016	4895	4927
3637	4166	3772	4684	4113	4493	4783	5054	5087	5296	4820	5258
3681	3812	3519	4187	4291	3494	4786	4599	4466	3634	4397	3747
3980	4313	4185	3586	4298	4261	4786	4547	3916	4925	4396	4682
4617	4414	4191	4012	4489	5029	4800	4962	4821	5062	5104	5077
3989	4420	4258	4450	4570	5099	4800	5021	5136	5261	5150	5318
4058	4306	4465	4462	4839	5079	4802	4987	4809	4621	4812	4883
3288	4683	2870	3297	4853	3789	4804	3672	3686	3254	4699	3550
3585	4637	5329	4565	4882	3718	4819	5024	4983	5047	3400	5191
3665	4498	4607	4491	4626	4907	4836	5318	5230	5271	4746	5066
3447	/	3684	4593	4650	4670	4846	4712	4540	5001	4795	3983
3717	4240	4088	4386	4296	4536	4853	4831	4881	4810	4529	4985
4552	4147	4300	4266	4261	4166	4867	4813	4855	5098	5153	4932
4591	4747	3546	4459	4638	3448	4881	4099	4810	4328	3872	4186
/	4171	4421	4440	4622	4785	4885	4746	4379	4667	4759	4441

3685	4368	4525	4591	4648	4022	4905	4767	4814	3292	3986	4871
3606	4196	3698	4673	4333	3847	4929	4577	4746	3888	4336	3933
3902	4199	4722	4305	4414	4118	4937	4683	4894	4260	4580	4027
3502	4258	4524	4543	4538	5043	4938	5214	5043	4883	3715	5261
3965	4450	3718	3961	4833	4155	4951	3871	4102	4277	4946	4047
3347	4003	4297	4449	4682	4047	5048	4757	4713	4079	4173	5029
3213	4496	3430	3401	4480	4351	5052	4883	4899	4892	4092	5167
3597	4180	2425	3613	3858	3566	5053	5005	5117	3181	4192	3884
3588	4405	4459	4232	4655	3652	5054	4791	4715	3874	3879	4635
3699	4047	3547	3580	4592	3935	5073	3391	4883	3879	3843	3750
3640	4666	2832	2724	4280	3995	5091	3385	3304	3973	4345	4044
3258	4766	4513	4473	4395	4216	5120	5181	5176	4414	4562	5185
3449	4919	3413	4514	4856	4303	5136	4586	5248	4547	4828	4507
3859	4765	4317	4783	4889	3339	5249	4712	5328	3282	3839	3981
3704	3562	3454	3397	3424	3786	/	3762	3793	2846	3983	3753
4632	3814	3759	3141	4195	3919	/	4271	3974	4455	3992	4233
4024	3746	3649	3924	3552	4158	/	3529	3331	3999	/	4259
