# Supplementary material for: Setting method of exit advance guide signs in mountainous expressway tunnel based on information quantization theory
Source: PLoS One. 2023 Feb 16;18(2):e0281842. doi: 10.1371/journal.pone.0281842 (PMC9934451; doi:10.1371/journal.pone.0281842)
Supplement: S4 Table — (PDF) [file pone.0281842.s004.pdf]

Reaction time of Exit advance guide signs of three-character place names

Combination 1	2	3	4	5	6	7	8	9	10	Combination 11
/	3535	3461	3248	3622	3767	3848	/	3813	4217	4154
3951	3833	3418	3620	3917	3684	3557	/	3458	3899	4033
/	3810	3672	3447	3872	3871	4329	3600	4184	/	4448
/	3094	3847	4154	4218	3983	4316	3693	4380	4254	4468
/	3487	3350	3859	4152	3370	4462	3730	4596	4569	4546
2866	/	3048	3426	4018	3471	3806	3749	3660	4339	3952
3391	3825	3538	3248	3768	3522	3527	3761	3785	3014	3666
3146	3049	/	3094	/	3427	4067	3829	3913	3983	4024
4272	3892	3395	3885	3764	4012	4066	3882	3545	4012	4335
3233	3285	3391	3613	4132	3438	4599	3901	4188	4033	4206
2853	3294	/	3535	3891	3683	4540	3965	3964	3647	3981
3965	3838	3484	3848	4026	3827	3671	3985	3857	3901	4100
3402	3987	3647	3581	3885	3802	4216	3994	4297	3814	4024
3715	4286	3732	3597	4055	4228	4546	4040	4427	4469	4186
3325	3427	3985	3655	4368	3715	4651	4084	4479	4460	4649
4047	3936	3635	3482	3672	3996	3892	4086	3625	3699	3826
3618	4047	3054	3505	3884	3700	3680	4093	4034	4046	3922
/	3886	3515	3899	4219	3828	4512	4095	4898	4703	4770
4066	4363	3915	3615	3983	3998	4031	4102	3763	3967	3938
3368	3843	3572	3591	3921	3851	4547	4106	4047	3984	4325
/	3827	3683	3515	4059	3994	/	4112	4220	/	/
/	3717	3813	4030	4149	4045	4465	4130	4492	4459	4357
/	4248	3962	4079	4380	4517	4624	4156	4499	4472	4606
4153	4427	3994	4046	4149	4017	4147	4199	3920	4436	4071
3787	4052	3404	3616	4021	3930	3855	4220	4124	4091	3970
4250	4026	3671	3885	4112	3999	3913	4226	3972	3982	4271
/	4035	3398	3647	4258	4079	4622	4228	3852	4283	4333
4467	4154	3881	4206	4125	4069	4002	4248	4013	4048	4426
3836	4106	3526	3632	4097	4049	3929	4263	4157	4098	4058
3038	3471	3294	3667	4076	3664	4071	4279	3988	4363	3960
3321	4083	3870	4846	4183	4959	4874	4282	4734	4381	4918
/	4061	3586	3780	4401	4084	4630	4294	4450	4370	4580
3404	3918	3717	3717	3933	3877	4579	4295	4373	4332	4350
4903	4343	3916	4334	4150	4239	4725	4304	4160	4079	4976
3125	3661	3412	3706	4234	4125	4129	4306	4552	4437	4262
4435	4264	3913	4149	4184	4101	4284	4312	3999	4279	4585
/	4233	3924	4292	4284	4031	4481	4322	4513	4448	4617
4646	4295	4020	4381	4206	4184	4403	4329	4020	4371	4664
4089	4650	3525	4580	4640	4939	5025	4333	4814	4945	4906
3844	4147	3612	3671	4106	4114	3993	4334	4335	4117	4146
3683	4396	4073	4846	4214	4379	4543	4334	4547	4606	4953
4059	4282	3833	4162	4274	4449	4216	4335	4512	4186	4487
3017	3025	3129	3468	3666	3720	4047	4336	4555	3903	4564
/	4249	4471	4417	4364	4240	4482	4339	4614	4681	4777
4255	4580	4368	4077	4463	4588	4905	4349	4705	5291	4863
3726	3730	4012	3897	4449	3948	4663	4358	4540	4626	4725
4050	4299	3954	4147	4159	4239	4663	4367	4434	4585	4379
/	4303	4481	4492	4415	4284	4583	4367	4630	4980	4870
4119	4813	3664	4491	4919	4631	4901	4369	4978	4958	5107
/	4366	4366	4182	4380	4567	4839	4380	4660	4479	4740
4296	4281	4371	4231	4632	4079	4683	4381	4814	4651	4947

4064	3770	3771	3937	4014	4347	4769	4383	4513	4806	4762
3443	4282	3939	3851	3936	3924	4662	4388	4466	4712	4579
3191	4524	4013	4726	4580	4373	4947	4415	4533	4459	4827
3965	4180	3735	4184	3995	4096	4617	4435	4494	4746	4970
4155	4464	4195	4099	4159	4073	4457	4440	4326	4515	4184
3865	4528	4104	4935	4387	4634	4553	4448	4672	4697	4467
/	3914	3588	4047	4387	3854	5115	4449	5026	4902	4839
3556	4416	4026	3984	3951	4148	4663	4455	4535	4760	4692
3963	4615	4612	4226	4727	4872	4531	4461	5002	4788	4947
3781	4624	4404	3080	4591	4617	3758	4463	4712	4981	4713
4438	4486	4186	4519	4181	4479	4725	4483	4640	4649	4501
/	4314	3940	3796	4529	4151	5115	4494	4533	4585	4587
/	4455	4426	4193	4427	4581	5101	4514	4684	/	4861
/	4739	3514	3621	3982	4497	4166	4570	4953	3338	3717
4984	4181	3684	3735	4050	4197	4657	4582	4450	4097	4680
4360	4719	4181	4436	4614	4530	3854	4597	4276	5132	4888
/	4203	3859	4146	4422	4126	5122	4609	5034	3998	5148
4004	4610	3962	4238	4180	4567	4831	4612	4663	4807	4985
/	4358	4173	4261	4960	4287	3992	4614	4773	3547	3863
4225	4558	4247	4198	4302	4136	4529	4614	4576	4637	4284
4193	4558	3947	4450	4762	4584	5019	4616	4495	4739	5160
/	4684	3114	3335	3726	4450	4451	4618	3365	3625	4024
3403	3489	3226	3537	4154	3765	4292	4619	4614	4367	4687
/	4380	4758	3736	4047	4394	4223	4652	3626	4201	4414
3437	3667	3301	3563	4183	3929	4792	4664	4683	4416	4847
4222	4439	4343	4379	4295	4261	5180	4693	5013	5087	5179
/	4467	4538	4630	4589	4532	4031	4701	4635	4126	4405
3455	3802	3505	3595	4338	4070	4862	4712	4688	4492	5136
3907	4325	3763	3973	4366	4204	4784	4713	4926	4535	4834
3546	3877	3694	4260	4547	4229	4881	4731	4765	4605	5154
4331	4610	4486	4251	4351	4181	4666	4745	4670	4758	4325
3717	4318	3887	3547	4694	4777	5094	4754	4871	5199	4692
3034	4694	4576	4057	4248	4786	4581	4785	4754	5319	4946
3549	4507	4246	4150	4251	4597	4737	4795	4716	4837	4948
4223	4491	4031	4058	4086	4617	4829	4797	4614	4985	4880
3266	3861	3620	3771	4402	4212	4195	4802	4598	4529	4355
3813	4603	4198	4172	4279	4621	4972	4812	4893	5031	4925
3799	4479	3853	4693	4742	4917	4925	4814	4776	4835	4819
4083	4613	4158	4570	4206	4736	5119	4829	4847	5017	5122
/	4520	4451	4440	4498	4648	3502	4831	4968	3751	3596
3948	4167	3880	4097	4459	4277	4354	4836	4638	4653	4754
/	4550	4586	3681	4048	4574	4451	4852	4328	4134	4529
4358	4671	4557	2653	4864	4340	4146	4866	5088	4963	5002
4113	4693	4389	4969	4403	4786	5172	4874	5016	5022	5213
3700	3891	3728	3337	4558	4417	3610	4919	4884	4816	5212
4345	4580	4350	4172	4664	4805	4813	4926	4965	4881	5117
2797	4130	4458	4071	4810	4654	5187	4934	4604	4739	4885
/	4426	3928	4303	4815	4713	4226	4938	5214	4164	4181
3601	4436	3824	4536	4463	4513	4825	4946	5014	4903	4847
3674	4883	4376	4870	4811	4513	4883	4955	5018	5114	5097
3869	4625	4358	4276	4482	4635	4972	4961	5034	5076	5224
3685	4302	4019	3533	3905	4728	3157	4994	4997	4870	3982
3783	4592	4099	3959	4360	4363	5225	5001	4447	4897	5024
/	3463	3645	4479	4714	3898	4570	5013	4082	5046	4999

/	4746	4514	4594	4336	4637	4881	5025	4749	4720	4896
3624	4872	4491	4747	5002	4926	5258	5037	5078	5197	4841
3732	5038	4661	4595	5064	5215	5092	5038	5143	5206	4081
3819	4349	4133	3752	4566	4713	3959	5040	5248	4813	4150
4215	4777	4850	3372	4487	5177	4181	5048	5121	5298	/
4140	4632	4514	4271	4810	4699	4714	5080	4916	4905	3948
4603	4824	4587	4884	4784	5070	5215	5087	5061	5263	5248
/	4821	4586	2847	3433	4923	3535	5126	3536	3858	3813
/	5062	4597	4312	4857	3776	5194	5147	5227	4920	4506
/	4650	4014	3687	3955	4906	4249	5152	3772	4288	4193
3919	4906	4395	5145	4896	4817	4937	5226	5118	5244	4625
3953	4946	4559	3825	4921	4970	4615	5237	5328	5310	4754
3213	3673	2584	3270	3781	3580	3619	/	3451	3546	3915
/	3312	2784	2859	3515	3397	3627	/	3668	3990	3961
/	3460	3252	3202	3536	3580	3639	/	3735	4048	4046
/	3489	3393	3587	3939	3721	4538	/	3782	4149	4216
/	3948	/	/	/	4251	/	/	/	/	/
