# Supplementary material for: Setting method of exit advance guide signs in mountainous expressway tunnel based on information quantization theory
Source: PLoS One. 2023 Feb 16;18(2):e0281842. doi: 10.1371/journal.pone.0281842 (PMC9934451; doi:10.1371/journal.pone.0281842)
Supplement: S5 Table — (PDF) [file pone.0281842.s005.pdf]

参加实验研究人员的知情同意书

尊敬的实验者：

您好！您将作为__________________实验研究的一名被试者，您左眼和右眼的视力/修正视力分别是______/______。本项实验将有人参加。为了确保本次实验的顺利进行并充分保障您的权益，在您同意参加之前，您需要清楚知道以下相关信息：

一、本实验的题目

基于信息论的隧道毗邻互通路段出口标志设置方法研究

二、主要研究者

尚婷；重庆交通大学交通运输学院教授，硕士生导师

吴逸飞；重庆奉建高速公路有限公司

吴鹏；重庆交通大学交通运输学院硕士

何虎成；重庆奉建高速公路有限公司

游宝；重庆交通大学交通运输学院硕士

三、您需要做的

如果您决定自愿参加这次实验，需保证自己的左眼和右眼的视力/修正视力是正常的。您需要识认视频中交通标志中的所有信息，识读完所有信息后快速点击键盘中相应的按钮。

四、自愿参加或退出

实验前请您对本次实验做详细的了解，研究者有义务向您提供与该实验有关的信息资料，为您解释您所关心的问题，然后有您自愿决定是否参加实验。

五、保密责任

本次实验所取得的结果与资料归本次试验的实施者无偿使用，但

您的合法权益不会因为本项目研究而受到侵犯，您的个人资料由本次试验的实施者保密。研究结果仅用于因科学目的的论文发表，不能用于商业盈利。

本知情同意书一式两份，本次试验的实施者和被试者各一份。

主要研究者：　　　　　　　　　　联系电话：

如果您已充分理解并同意上述内容，请在本知情同意书下方签字确认。

被试者签名：　　　　　　　　　　　　　　联系电话：

时间：　　年　　月　　日
