# Supplementary material for: Setting method of exit advance guide signs in mountainous expressway tunnel based on information quantization theory
Source: PLoS One. 2023 Feb 16;18(2):e0281842. doi: 10.1371/journal.pone.0281842 (PMC9934451; doi:10.1371/journal.pone.0281842)
Supplement: S6 Table — (PDF) [file pone.0281842.s006.pdf]

Informed Consent Form of the Researchers Participating in the Experiment

Dear experimenter,

Hello!

You will be a participant in the___________________________experimental study. Your left eye and right eye vision/corrected vision are_____/______respectively. There will be___________participants in this experiment. In order to ensure the smooth progress of this experiment and fully protect your rights, before you agree to participate, you need to know the following relevant information:

1. The topic of this experiment

   Setting Method of Exit Advance Guide Signs in Mountainous Expressway Tunnel Based on Information Quantization Theory

2. Main researcher

   Ting Shang Professor, School of Transportation, Chongqing Jiaotong University, Master Instructor
   Yifei Wu, Department of Engineering Management, Chong Qing Feng Jian Expressway Co., Ltd
   Peng Wu, School of Transportation, Chongqing Jiaotong University
   Hucheng He, Department of Engineering Management, Chong Qing Feng Jian Expressway Co.,
   Bao You, School of Transportation, Chongqing Jiaotong University

3. What you need to do

   If you decide to voluntarily participate in this experiment, you need to ensure that your left and right eyesight/corrected eyesight is normal. You need to recognize all the information in the traffic signs in the video. After reading all the information, quickly click the corresponding button on the keyboard.

4. Voluntary participation or withdrawal

   Before the experiment, please have a detailed understanding of this experiment. The researcher is obliged to provide you with information related to the experiment, explain your concerns, and then you voluntarily decide whether to participate in the experiment.

5. Duty of confidentiality

   The results and information obtained in this experiment are used by the implementer of this experiment free of charge, but your legal rights and interests will not be infringed by the research of this project, and your personal information will be kept confidential by the implementer of this experiment. Research results

are only used for the publication of papers for scientific purposes and cannot be used for commercial profit.

This informed consent form is in duplicate, one for the implementer and one for the subject.

Main researcher: Contact number:

If you have fully understood and agreed to the above content, please sign at the bottom of this informed consent form for confirmation.

Participant's signature: Contact number:

Date
