# Supplementary material for: Setting method of exit advance guide signs in mountainous expressway tunnel based on information quantization theory
Source: PLoS One. 2023 Feb 16;18(2):e0281842. doi: 10.1371/journal.pone.0281842 (PMC9934451; doi:10.1371/journal.pone.0281842)
Supplement: S1 File — (DOCX) [file pone.0281842.s007.docx]

**Annex 1**

**This form should be on page 19 at line number 368**

**Table 8. Layout form and information content of exit warning signs with different combinations of elements**

| Number | Combination | Signs contain "two characters "place name | | | Signs contain "three characters "place name | | |
| --- | --- | --- | --- | --- | --- | --- | --- |
|  |  | layout | Layout size(*L*×*H*)/cm | amount of information/bits | layout | Layout size(*L*×*H*)/cm | amount of information/bits |
| 1 | road name + arrow | 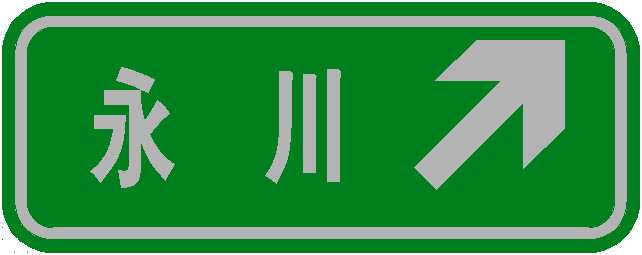 | 236.5×110 | 9.613 | 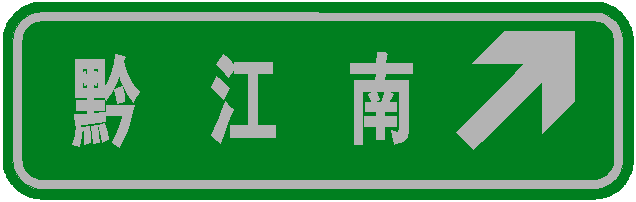 | 297×110 | 14.192 |
| 2 | Road name + arrow + exit number | 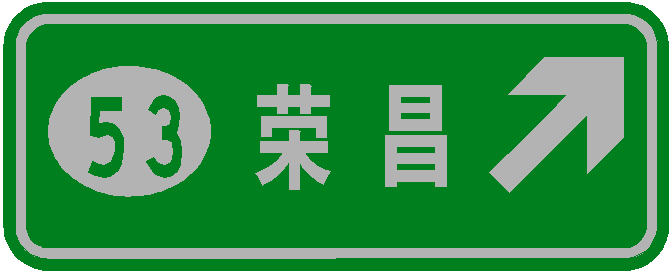 | 320×110 | 11.033 | 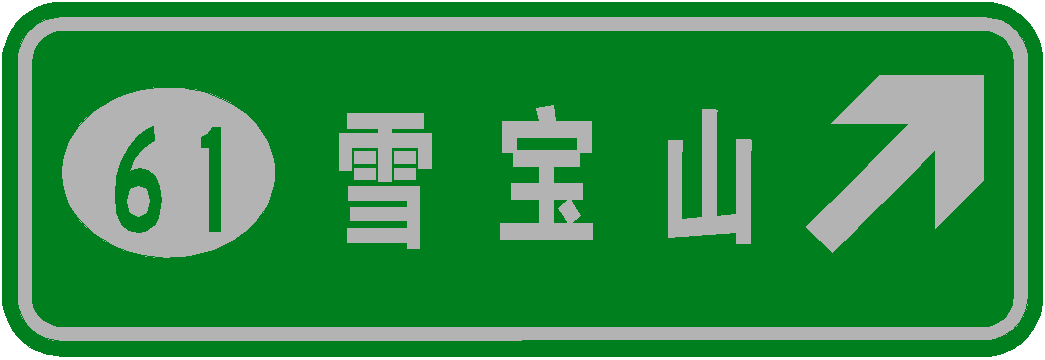 | 380.5×110 | 15.612 |
| 3 | road name + distance | 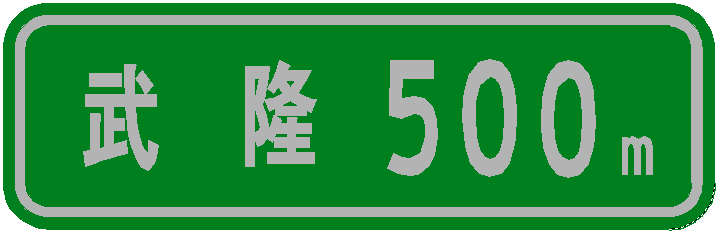 | 308×110 | 11.872 | 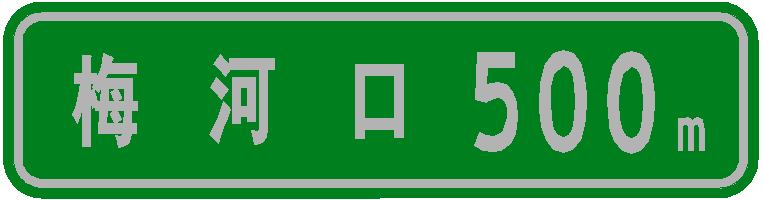 | 368.5×110 | 16.450 |
| 4 | road name + distance + arrow | 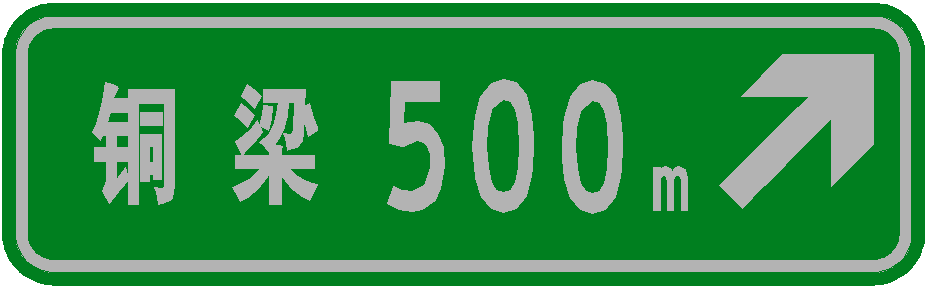 | 385×110 | 12.074 | 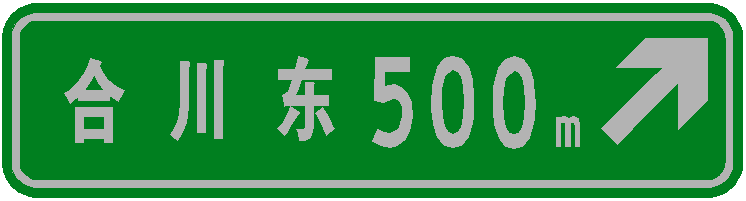 | 445.5×110 | 16.652 |
| 5 | Road name + distance + exit number | 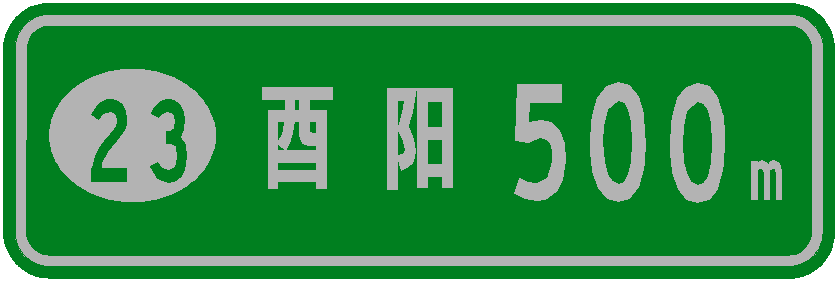 | 391.5×110 | 13.291 | 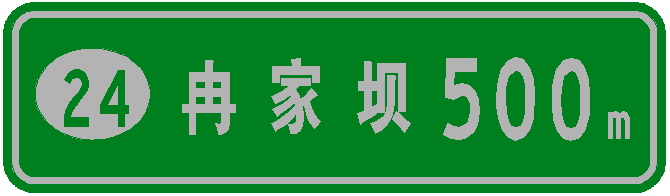 | 452×110 | 17.870 |
| 6 | Road name + distance + arrow + exit number | 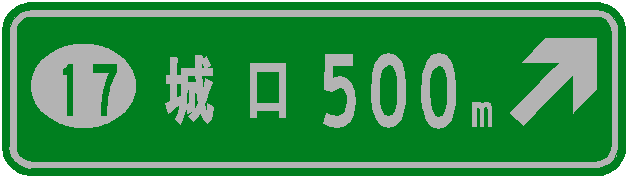 | 468.5×110 | 13.493 | 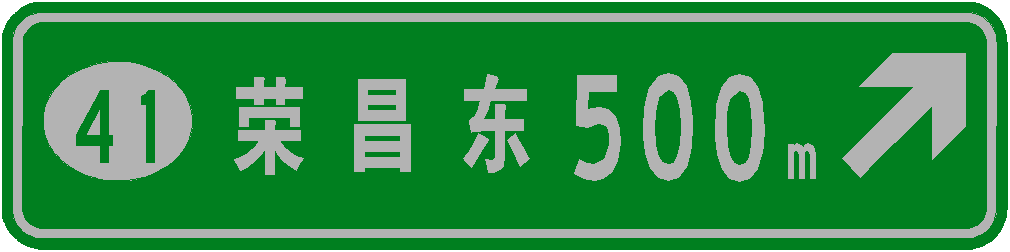 | 529×110 | 18.072 |
| 7 | Road name + arrow + English | 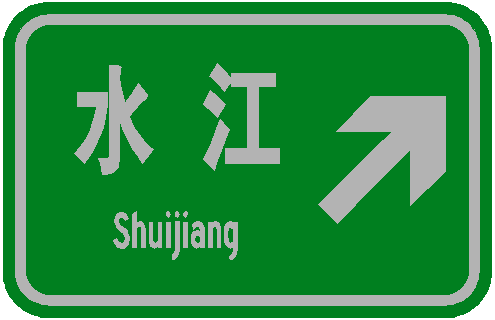 | 236.5×148.5 | 13.290 | 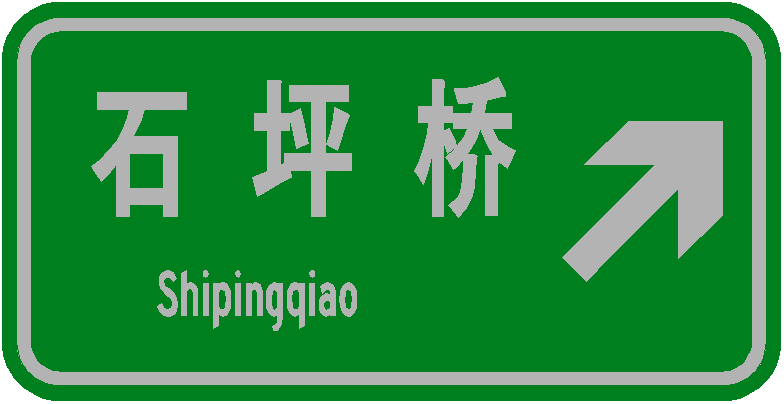 | 297×148.5 | 18.685 |
| 8 | Road name+arrow+English+exit number | 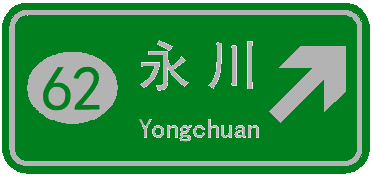 | 320×148.5 | 14.710 | 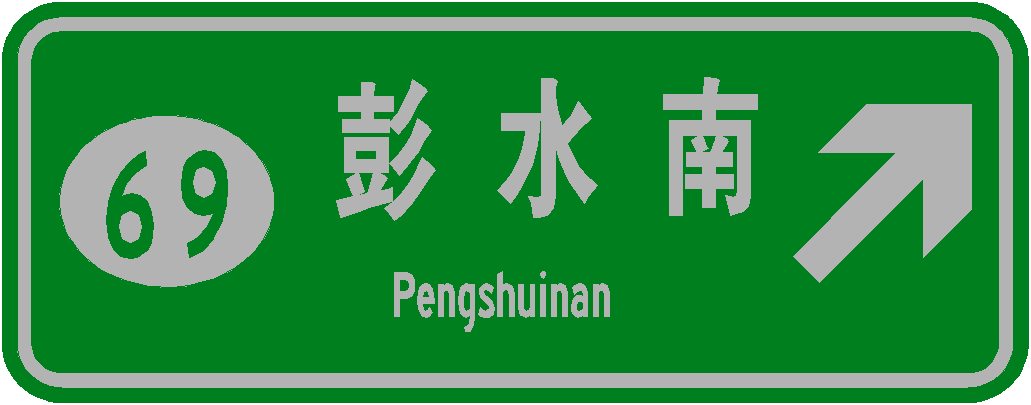 | 380.5×148.5 | 20.105 |
| 9 | Road name + distance + English | 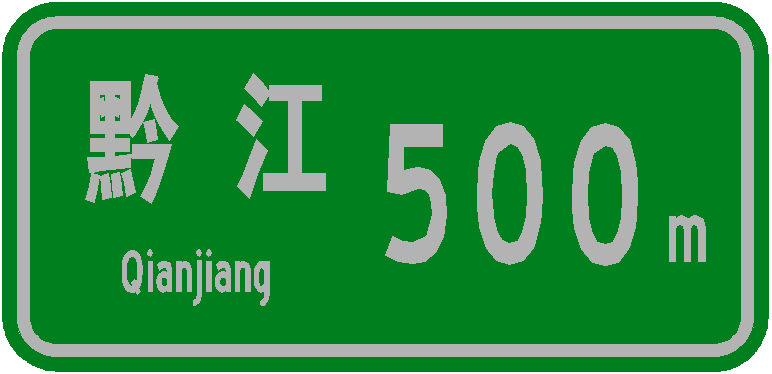 | 308×148.5 | 15.548 | 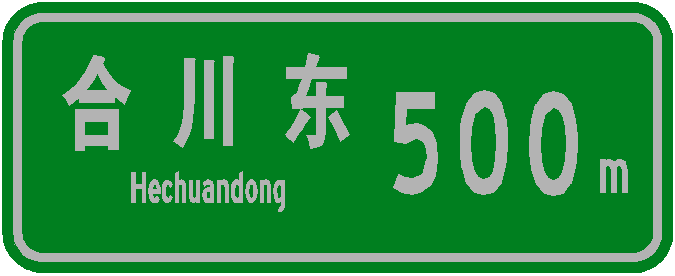 | 368.5×148.5 | 20.944 |
| 10 | Road name + distance + arrow + English | 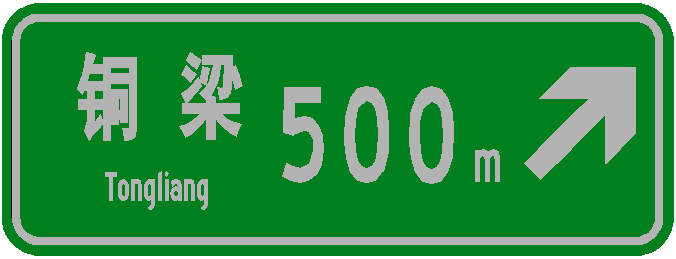 | 385×148.5 | 15.750 | 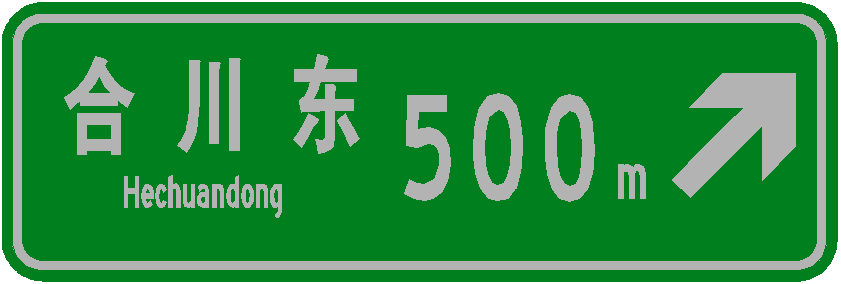 | 445.5×148.5 | 21.146 |
| 11 | Road name + distance + English + exit number | 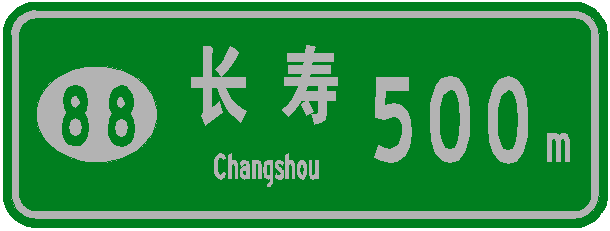 | 391.5×148.5 | 16.968 | 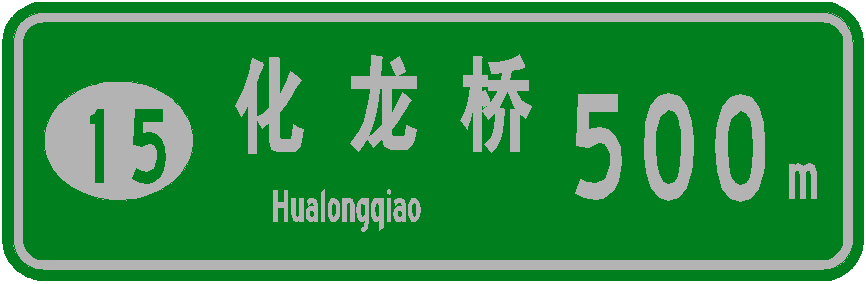 | 452×148.5 | 22.363 |
| 12 | Road name + distance + arrow + English + exit number | 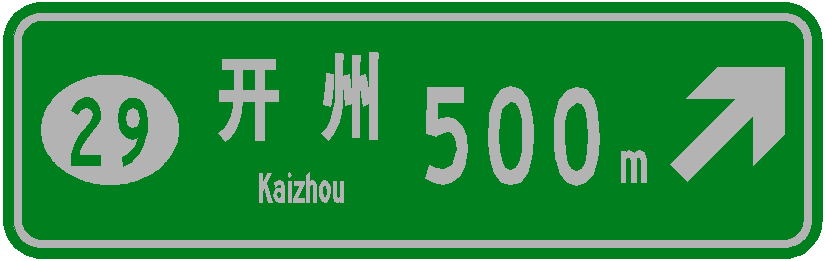 | 468.5×148.5 | 17.170 | 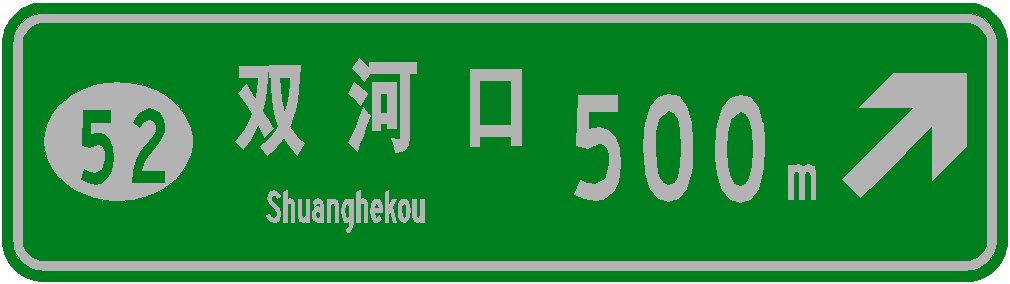 | 529×148.5 | 22.565 |
